# Use of Cyclodextrins in Anticancer Photodynamic Therapy Treatment

**DOI:** 10.3390/molecules23081936

**Published:** 2018-08-02

**Authors:** Amina Ben Mihoub, Ludivine Larue, Albert Moussaron, Zahraa Youssef, Ludovic Colombeau, Francis Baros, Céline Frochot, Régis Vanderesse, Samir Acherar

**Affiliations:** 1 Laboratoire de Chimie Phusique Macromoléculaire, Université de Lorraine, CNRS, LCPM, F-54000 Nancy, France; amina.ben-mihoub@univ-lorraine.fr (A.B.M.); ludivine.larue@univ-lorraine.fr (L.L.); albert.moussaron@univ-lorraine.fr (A.M.); ludovic.colombeau@univ-lorraine.fr (L.C.); regis.vanderesse@univ-lorraine.fr (R.V.); 2 Laboratoire Réactions et Génie des Procédés, Université de Lorraine, CNRS, LRGP, F-54000 Nancy, France; zahraa.youssef@univ-lorraine.fr (Z.Y.); francis.baros@univ-lorraine.fr (F.B.); celine.frochot@univ-lorraine.fr (C.F.)

**Keywords:** cylodextrin, photosensitizer, photodynamic therapy, cancer, inclusion complex, conjugate, nanoassembly, nanoparticle, porphyrinoid, fullerene

## Abstract

Photodynamic therapy (PDT) is mainly used to destroy cancerous cells; it combines the action of three components: a photoactivatable molecule or photosensitizer (PS), the light of an appropriate wavelength, and naturally occurring molecular oxygen. After light excitation of the PS, the excited PS then reacts with molecular oxygen to produce reactive oxygen species (ROS), leading to cellular damage. One of the drawbacks of PSs is their lack of solubility in water and body tissue fluids, thereby causing low bioavailability, drug-delivery efficiency, therapeutic efficacy, and ROS production. To improve the water-solubility and/or drug delivery of PSs, using cyclodextrins (CDs) is an interesting strategy. This review describes the in vitro or/and in vivo use of natural and derived CDs to improve antitumoral PDT efficiency in aqueous media. To achieve these goals, three types of binding modes of PSs with CDs are developed: non-covalent CD–PS inclusion complexes, covalent CD–PS conjugates, and CD–PS nanoassemblies. This review is divided into three parts: (1) non-covalent CD-PS inclusion complexes, covalent CD–PS conjugates, and CD–PS nanoassemblies, (2) incorporating CD–PS systems into hybrid nanoparticles (NPs) using up-converting or other types of NPs, and (3) CDs with fullerenes as PSs.

## 1. Introduction

### 1.1. Cancer and Treatments

Cancer has more than 277 different types and is the second leading cause of global death after cardiovascular diseases [1]. Through its GLOBOCAN project, the International Agency for Research on Cancer (IARC), a semi-autonomous unit of the World Health Organization (WHO), has estimated in 2012 approximately 14.1 million new global cases of cancer and 8.2 million global deaths, with slightly more incidence cases and death effects on men than women (men:women ratios of 53:47 and 57:43 for cases and deaths, respectively) [2]. The top five killer cancers for both sexes combined are reported in Table 1, and these data revealed that lung (19.4% of the total), liver (9.1%), stomach (8.8%), colorectal (8.5%) and breast (6.4%) are the five most common causes of cancer death. Based on these estimates and on the prediction that the number of new global cancer cases is expected to increase by 70% over the next two decades, the *Global action plan for the prevention and control of noncommunicable diseases 2013‒2020* was endorsed in 2013 by the World Health Assembly [3]. This global action plan has nine goals, including a 25% reduction in global premature mortality by 2025 from the four types of noncommunicable diseases, i.e., cardiovascular and chronic respiratory diseases, cancer, and diabetes, relying in particular on greater coordinated and coherent actions at all levels (local, national, and international). Cancer as a whole is responsible of nearly one-sixth of global deaths and the latest available estimates of cancer mortality from the Institute for Health Metrics and Evaluation (IHME) indicate 8.9 million global deaths in 2016, whose most common causes of death are the cancers of lung (19.2% of the total), stomach (9.4%), colorectal (9.3%), liver (9.3%), and breast (6.1%) (Table 1) [4]. As a general point, it is noted that around 70% of global cancer deaths occur in low and middle-income countries, and one-third of global deaths are due to the five-leading behavioral and dietary risk factors, which are obesity, low intakes of fruit and vegetables, physical inactivity, tobacco use, and alcohol consumption.

There are many types of cancer treatment [5,6], and the health care team is going to establish a treatment plan for cancer depending on various parameters, such as the type, stage, and spread of cancer, and the patient’s age and general health. The aim of the treatment planning for cancer, with the use of a single or combination therapy, is to treat and cure cancer but in other cases to control the cancer’s proliferation or minimize symptoms for as long as possible. The most common cancer treatments are surgery [7,8], radiation therapy [9,10], and chemotherapy [11]. Surgery can be used to diagnose and treat many types of cancer, and consists in removing all or part of a tumor, especially when the cancer has not spread to other parts of the body. Radiation therapy is intended to deprive the cancer cells of their cell division and multiplication processes with the aim of damaging or destroying cancer cells using radiation, such as X-rays, gamma rays, electron beams, or protons. Chemotherapy, for its part, can be implemented for killing or controlling the growth of cancer cells, and is based on the use of one drug or a combination of drugs. These three cancer treatments have side effects, and are not always effective to treat and cure cancer. In addition, the duration, frequency, and the number of chemotherapy or radiation therapy cycles produce negative side effects that tend to gradually get worse over time. Based on these findings, researchers have developed further cancer therapies to improve the effectiveness of treatments while reducing undesired side effects, such as immunotherapy [12,13], hormone therapy [14], gene therapy [15,16], cryotherapy [17], targeted cancer therapies [18,19], stem cell transplant [20,21], thermal therapy [22,23], and photodynamic therapy [24,25,26].

### 1.2. Photodynamic Therapy for Cancer Treatment

**Figure 1:**
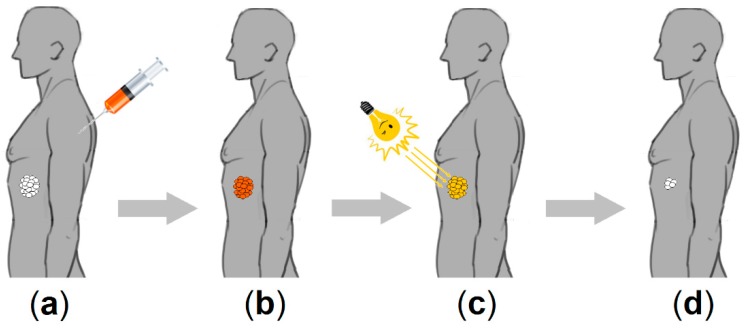
Two-step photodynamic therapy (PDT) cancer treatment process involving: (**a**) photosensitizer (PS) injection; (**b**) PS accumulation in the tumor; (**c**) PS activation by light; (**d**) reactive oxygen species (ROS) production and tumor damage response.

The basic principle of photodynamic therapy (PDT) is to combine the action of three components: (1) a photoactivatable molecule or photosensitizer (PS), (2) a certain kind of light typically in the visible spectrum, and (3) naturally occurring molecular oxygen (triplet oxygen, ^3^O_2_) [27]. The PDT as cancer treatment (Figure 1) involves the administration of a PS to patients, followed by visible light excitation. The excited PS then reacts with molecular oxygen to produce reactive oxygen species (ROS) [28,29], including singlet oxygen (^1^O_2_) [30], leading to cellular damage [31,32].

**Figure 2:**
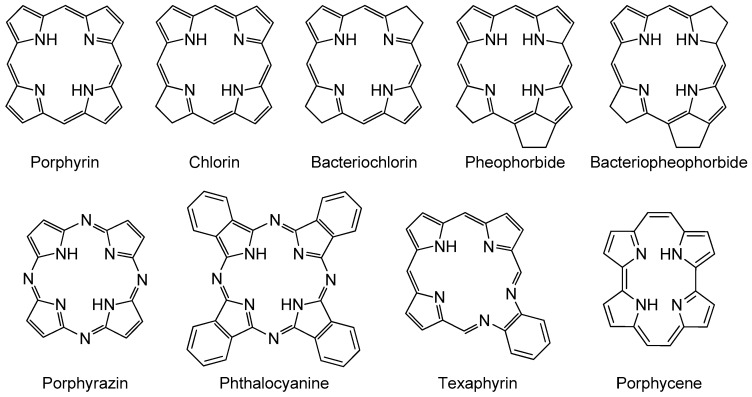
Examples of molecular skeletons of porphyrinoid-based PSs.

The PDT story is well-documented [33,34], and the light is known for its therapeutic effect for millennia with the first uses of light in Chinese, Egyptian, or Indian civilizations for the treatment of numerous diseases such psoriasis, vitiligo, and rickets dating back to over 3000 years ago [35,36]. PS employed in PDT are divided into four categories, namely, first, second, third, and fourth-generation PS, according to the chronological development and the evolution of conceptual approaches to reduce their disadvantages for a PDT treatment. Although there are various types of PSs, most of them belong to the porphyrinoid groups (Figure 2).

Historically and from a clinical point of view, the use of PDT for cancer in humans began in the 1970s with the study of the effects of hematoporphyrin derivatives (HpD) and light on five patients with bladder cancer [37] and on 25 patients with a large series of malignant tumors [38]. The findings of these studies have paved the way in 1993 for the first clinically approved PS (Photofrin^®^, a purified fraction of HpD) to treat bladder cancer in Canada. However, the use of Photofrin^®^ (a first-generation PS) for PDT cancer treatments is confronted by several limitations, such as a fairly low absorption band close to 630 nm driving the need for high PS injection to produce enough ROS to result in tumor destruction. Ideally, a good PS (second-generation PS) should have a strong absorption band in the 650 nm to 800 nm range with a high extinction coefficient (ε_max_ above 50,000 M^−1^·cm^−1^) allowing light penetration deeper into tissue up to 1 cm, and thus offering the possibility of producing ROS to kill tumor cells under centimeter-thick tissue. In addition, an ideal PS (third-generation PS, i.e., second-generation PS conjugated with a targeting agent) should also be able to specifically accumulate in cancer tissues compared with in healthy tissues. Finally, concerning the theranostic approach, an optimal PS (fourth-generation PS, i.e., third-generation PS conjugated with an imaging agent) should also be able to combine tumor-targeted PDT treatment and precise tumor diagnosis. To date, hundreds clinical trials concerning PDT (alone or in combination with other therapies) were investigated, and there are several reviews in the literature relating to the use of PDT in clinical trials or preclinical studies [26,39,40]. An overview of the few clinically approved PSs for PDT as a cancer treatment are listed in Table 2.

Another problem faced by many PSs is their lack of solubility in water and the body’s tissue fluids, thereby causing amongst other things the low bioavailability, drug-delivery efficiency, and therapeutic efficacy of PSs in vivo, which restricts their clinical applications.

Among various strategies to improve the water solubility and/or drug delivery of PSs [25], e.g., by using hydrophilic substituents (cationic or anionic groups [41,42,43], amino acids [44,45], peptides [46,47], sugars [48,49,50], polyethylene glycol derivatives [51], liposomes [52,53], or organic and inorganic nanoparticles [54,55,56,57,58,59,60], cyclodextrins (CDs) represent interesting hydrophilic substituents to resolve these challenges and help in the development of drugs with enhanced water solubility [61] as well as enhanced drug delivery [62,63,64,65].

### 1.3. Cyclodextrins for Enhancing Drug Solubility and Delivery

**Figure 3:**
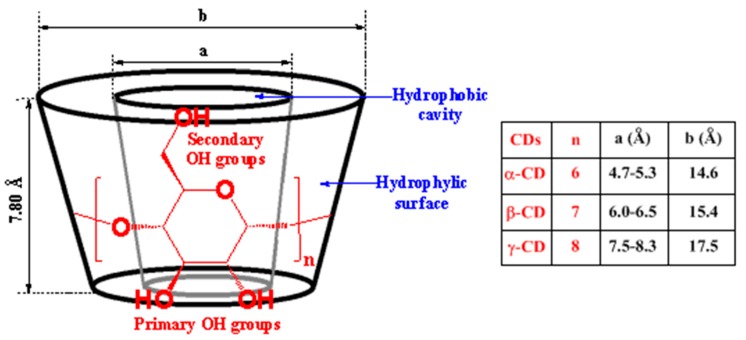
Chemical structure of natural α, β, and γ-cyclodextrins.

CDs are a class of natural cyclic oligosaccharides produced during the bacterial digestion of cellulose that consist of (α-1,4)-linked α-D-glucopyranose units. There are three types of natural CDs, named α-CD, β-CD, and γ-CD (Figure 3), depending on the number of glucopyranose units (six to eight units) linked together. CDs are water-soluble, biocompatible, crystalline, and non-hygroscopic substances. From a structural point of view and due to the chair conformation of glucopyranose units, CDs are toroidal (truncated cone), with a nonpolar tapered cavity and a hydrophilic tapered surface (primary hydroxyl groups at the narrow edge and secondary hydroxyl groups at the wider edge).

The discovery of CD by Villiers was made in 1891 from the fermentation of potato starch by the butyric ferment *Bacillus amylobacter*, and the story of CDs is well-documented [66,67,68]. It was not until the 1980s that the first CD applications in the pharmaceutical and food industries would appear thanks to the possible industrial-scale production of the three natural CDs, making CDs commercially available at a reasonable price. From the 1980s to date, CDs (especially β-CD) are found applications in various industrial sectors [69,70,71,72,73] such as medicine [74], pharmacy [62,75,76,77], food [78,79,80,81], cosmetics [82], chromatography [83,84,85,86,87,88], catalysis [89,90,91], biotechnology [92,93], textiles [94,95,96], and the environment [97,98].

The use of CDs in the medicinal and pharmaceutical applications comes from, among other things, their ability to (1) encapsulate hydrophobic drugs, i.e., the formation of CD-drug inclusion complexes, and (2) formulate orodispersible tablets of poorly-soluble drugs, i.e., CDs as vehicles in tabletting. These two important factors make the drug available at the surface of the biological barrier, and thereby lead to an improvement of the water solubility, stability, bioavailability, release, and under certain conditions, permeability of the drug. In addition to these natural CDs, researchers also developed derived CDs by the modification and polymerization of natural CDs with the aim of improving biopharmaceutical and physicochemical drug properties, and the complexation efficiency of natural CDs. An overview of natural and derived CDs and their characteristics (water solubility and molecular weight) is listed in Table 3.

**Figure 4:**
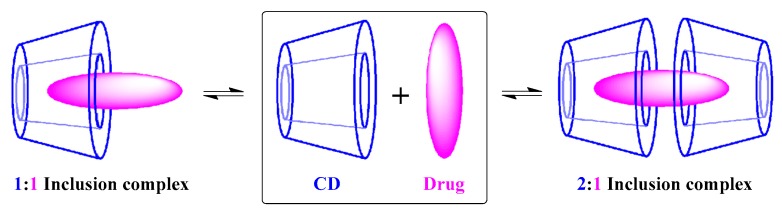
Two examples of stoichiometry adopted by CD–drug inclusion complexes.

As stated above, one main interest of CDs both in research and applications stem from CDs (hosts) being able to selectively and reversibility form host–guest inclusion complexes with a large variety of guest molecules in aqueous solutions [99,100,101,102,103,104]. The host–guest inclusion complex formation is based on the insertion (encapsulation) of the guest molecule into the internal cavity of CD, which is driven by the release of enthalpy-rich water molecules from the CD’s cavity, the release of conformational strain, and the establishment of several interactions between the guest molecule and CD via hydrogen bonds, van der Waals forces, electrostatic interactions, hydrophobic interactions, and charge-transfer interactions. The ability of CDs to form reversible host–guest inclusion complexes was first described by Cramer in the early 1950s [105], and is governed by two key factors: the steric effect, and the thermodynamic interactions between the size/shape of the CD’s cavity and the guest molecule. Depending on the structure and properties of the drug (guest molecule), different host–guest inclusion complexes and stoichiometry can be obtained (Figure 4). The host–guest complexes provide refuge and protection for guest molecules in the biological environment, leading to an enhancement in the water solubility, stability, and bioavailability of the guest molecules.

## 2. Cyclodextrins for Anticancer Photodynamic Therapy

### 2.1. General

CDs made their appearance in the field of cancer treatment in the 1990s. The use of natural and derived CDs as drug delivery carriers for various poorly water-soluble chemotherapeutic cytotoxic anticancer drugs was recently discussed in an exhaustive review [106]. 

Concerning CDs in anticancer PDT treatment, the first results of these researches were introduced in 1992 by Morgan et al. during the 6th International Cyclodextrin Symposium held in Chicago [107], and aimed to use γ-CD and HP-γ-CD as solubilizing agents instead of Chremophore to improve in vivo the solubility and concentration efficiency of the synthetic PS, tin etiopurpurin, in the tumor cells.

This review focuses only on the use of CDs to improve the in vitro or/and in vivo biological effect of PSs in anticancer PDT treatment without including the addition of active targeting ligands.

### 2.2. Cyclodextrin–Photosensitizer Systems

CD–PS systems can be formed by three types of binding modes of PSs with CDs. This paragraph will focus on the CD–PS system formation by non-covalent binding (CD–PS inclusion complexes, Section 2.2.1.), covalent binding (CD–PS conjugates, Section 2.2.2.), and non-specific external binding (CD–PS nanoassemblies, Section 2.2.3.)

#### 2.2.1. Non-Covalent Cyclodextrin–Photosensitizer Inclusion Complexes

CDs are known as an attractive option for the PDT of cancers due to their ability to interact with a large variety of PSs, leading to the formation of non-covalent inclusion complexes. This part of the review focuses on the in vitro or/and in vitro biological effect of CD-PS inclusion complexes in anticancer PDT. However, with respect to articles involving the potential use of CD-PS inclusion complexes in anticancer PDT (without in vitro and/or in vivo biological studies), we can emphasize that several studies have estimated benefit of CD–PS inclusion complexes on the physicochemical properties of PSs. For porphyrinoid PSs, various studies showed that CD–PS inclusion complexes could (1) increase some of the photophysical properties of PSs (fluorescence intensity [108,109], tripling the lifetime in neutral aqueous solutions [110], ^1^O_2_ production [111], and quantum yield [112] in aqueous solutions), (2) reduce some drawbacks of PSs (such as poor water solubility [113], self-aggregation [111,114], protonation of pyrrole nitrogens [110], metalation [110], thermal degradation [115]), and (3) enhance drug delivery [110,115] and lipid membrane penetration [116]. Similar results were also obtained for non-porphyrinoids PSs, i.e., the improvement of photophysical properties (fluorescence lifetime, emission, and quantum yield) [117], drug delivery [117,118], and photostability [119]. The studies that have presented an in vitro or/and in vivo biological anticancer PDT evaluation of CD–PS inclusion complexes are summarized below.

- Porphyrinoid Photosensitizers

**Figure 5:**
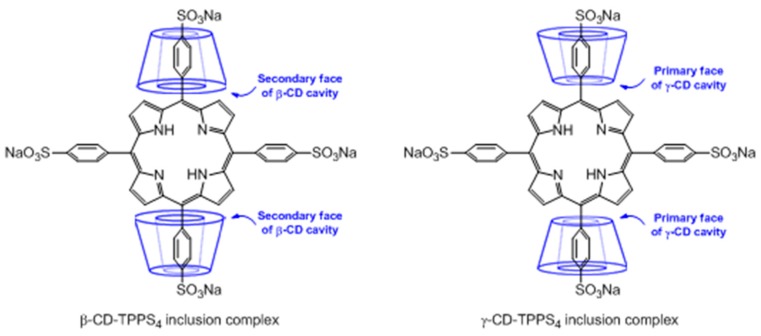
Two examples of typical geometry adopted by CD–PS inclusion complexes between β-CD and γ-CD with a porphyrinoid PS (*meso*-tetrakis(4-sulfonatophenyl)porphyrin, TPPS_4_). Adapted from Ribó; Farrera; Valero; Virgili [114].

Generally, host–guest inclusion complexes between CDs and porphyrinoid PSs are formed by encapsulating the “aryl” substituent portion of the PS into the fairly large cavity of CD. Across all three natural types of CD, α-CD does not allow the formation of inclusion complexes due to its small cavity, while β-CD and γ-CD can generate inclusion complexes typically through the primary face of the β-CD cavity and the secondary face of the γ-CD (Figure 5). Different CD–PS inclusion complexes’ stoichiometry can be obtained, but 1:1 and 2:1 stoichiometries are the most common. 

In 2003, Kolàrovà et al. [120] studied the in vitro phototoxicity of two PSs models: *meso*-tetrakis(4-sulphonatophenyl)porphyrin (TPPS_4_) and its zinc metallocomplex (ZnTPPS_4_) on G361 human melanoma cells in the presence and absence of 2-hydroxypropyl-β-cyclodextrin (HP-β-CD). Based on the biological studies performed on G361 human melanoma cells, the inclusion complexes of each PS with HP-β-CD showed a lower cytotoxicity and higher phototoxicity compared to the PSs alone. They already found that the most effective system was the HP-β-CD-ZnTPPS_4_ inclusion complex, since the IC_50_ value was 12.5 mg/mL at a light radiation dose of of 10 J/cm^2^.

In 2005, the same team [121] investigated the influence of the HP-β-CD-ZnTPPS_4_ inclusion complex on the in vitro phototoxic properties of ZnTPPS_4_ using the same G361 human melanoma cells. According to the cellular uptake studies, the prepared HP-β-CD-ZnTPPS_4_ inclusion complex showed a good penetration through the cell membrane. Indeed, the inclusion complex accumulation was dependent of PS concentration and incubation time, and the best uptake was found at 3 µM of the inclusion complex after 48 h of incubation. The in vitro phototoxicity efficiency after 24 h of incubation of G361 cells with ZnTPPS_4_ (10 µM) and HP-β-CD (1 mM) and under light irradiation (12.5 J/cm^2^, 24 V/250 W) showed DNA breaks in G361 cells. The authors concluded that binding ZnTPPS_4_ to HP-β-CD may improve its PDT efficiency against malign melanoma.

**Figure 6:**
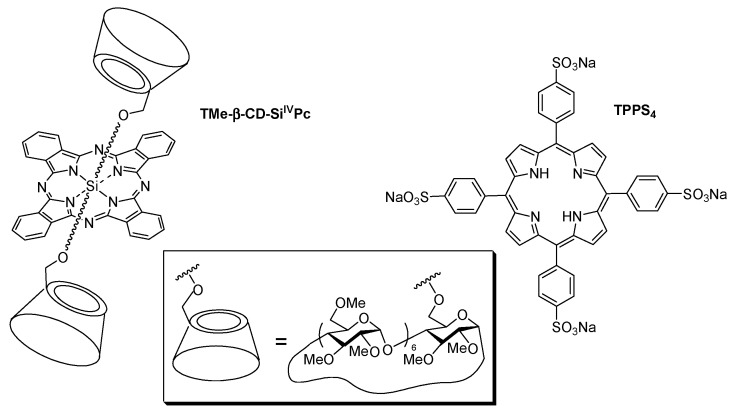
Schematic representation of TMe-β-CD-Si^IV^Pc and TPPS_4_. Adapted from Leng; Choi; Lo; Ng [122].

In 2007, Lo et al. [122] studied the supramolecular hetero-arrays of tetrapyrrole derivatives held by host–guest interactions (1:1 stoichiometric ratio). The nanostructure contained heptakis(2,3,6-tri-*O*-methyl)-β-cyclodextrin-conjugated silicon(IV) phthalocyanine (TMe-β-CD-Si^IV^Pc) complexed with the tetrasulfonated porphyrin (TPPS_4_) (Figure 6).

**Figure 7:**
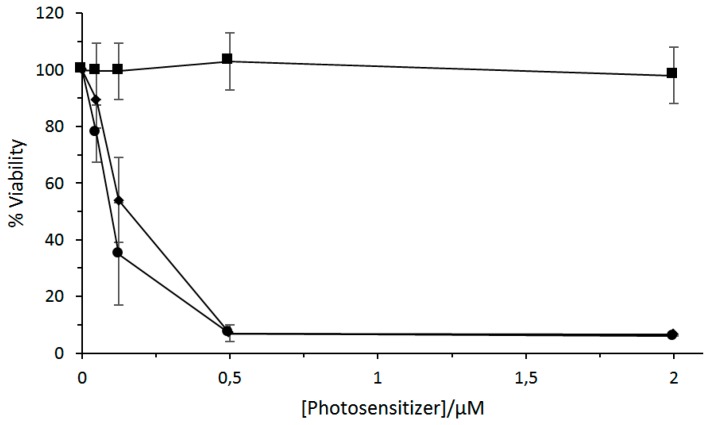
In vitro photocytotoxicity of TMe-β-CD-Si^IV^Pc (♦), TPPS_4_ (■), and the 1:1 host–guest complex (●) against HT29 cells under red light illumination (λ > 610 nm, total fluence = 48 J/cm^2^). Adapted from Leng; Choi; Lo; Ng [122].

The authors studied briefly the in vitro PDT efficiency of TMe-β-CD-Si^IV^Pc and TPPS_4_, and the 1:1 inclusion complex on HT29 human colon adenocarcinoma cells upon illumination with a red light (λ > 610 nm, total fluence = 48 J/cm^2^; Figure 7). They concluded that the porphyrin enhances the water solubility and facilitates the formulation of phthalocyanine through complex formation.

**Figure 8:**
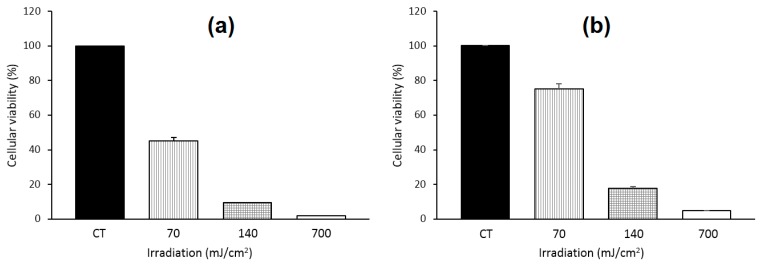
Cellular viability of J774-A1 after treatment with (**a**) CIAlPc/HP-β-CD and (**b**) CIAlPc/β-CD complexes under various irradiation. The cells were exposed to increasing doses of light at 70 mJ/cm^2^, 140 mJ/cm^2^, and 700 mJ/cm^2^ (**a**) *p*-values < 0.001 and (**b**) *p*-values < 0.05 in relation to the respective control. Data is expressed as means ± SD (*n* = 3). Adapted from Silva; Simioni; Tedesco [123].

In 2011, Silva et al. [123] studied a complex 2:1 of β-CD or HP-β-CD with a chloro-aluminum phthalocyanine (ClAlPc). The photophysical properties of the inclusion complexes in deuterated ethanol showed a fluorescence quantum yield of 0.38 and 0.09 for ClAlPc/β-CD and ClAlPc/HP-β-CD, respectively, and a ^1^O_2_ quantum yield of 0.65 and 0.80 for ClAlPc/β-CD and ClAlPc/HP-β-CD, respectively; the authors concluded that the ClAlPc/Hp-β-CD inclusion complex should be the best candidate for PDT application. An in vitro study on J774 mouse macrophage tumor cells with a CIAlPc/HP-β-CD inclusion complex under various irradiation (70 mJ/cm^2^, 140 mJ/cm^2^, and 700 mJ/cm^2^) indicated a decrease of the cell viability depending on the applied dose light (Figure 8).

**Figure 9:**
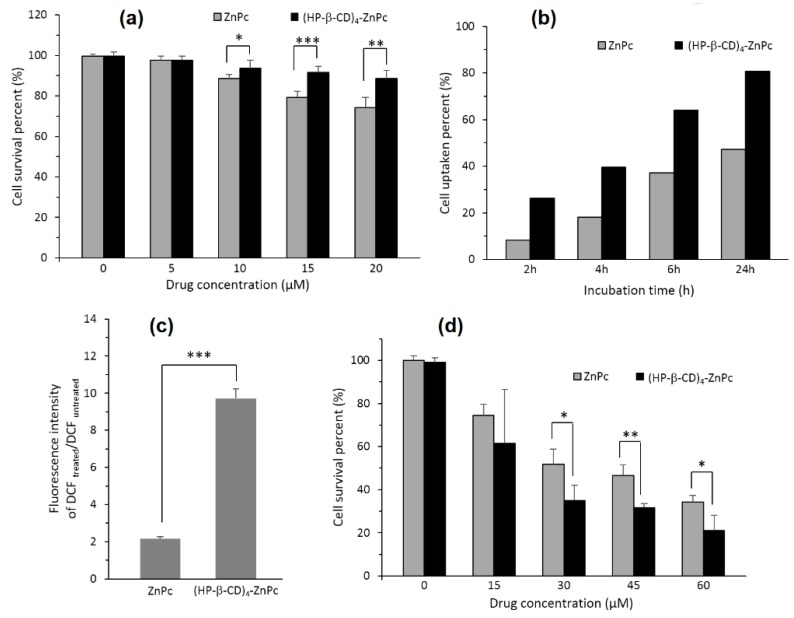
(**a**) Dark toxicity of zinc phthalocyanine (ZnPc) and (HP-β-CD)_4_-ZnPc (drug concentration was calculated by ZnPc. * *p* < 0.05, ** *p* < 0.01, *** *p* < 0.001 ZnPc vs. (HP-β-CD)_4_-ZnPc). (**b**) Human cervical carcinoma (HeLa) cellular uptake of ZnPc and (HP-β-CD)_4_-ZnPc ([ZnPc] = [(HP-β-CD)_4_-ZnPc] = 5 µM). (**c**) In vitro ROS production induced by ZnPc and (HP-β-CD)_4_-ZnPc ([ZnPc] = [(HP-β-CD)_4_-ZnPc] = 5 µM, irradiation time = 5 min, *** *p* < 0.001 ZnPc vs. (HP-β-CD)_4_-ZnPc). (**d**) Light toxicity of ZnPc and (HP-β-CD)_4_-ZnPc with different drug doses and 5 min irradiation (* *p* < 0.05, ** *p* < 0.01, ZnPc vs. (HP-β-CD)_4_-ZnPc). Adapted from Lu; Ma; Xuan; Wang; Zhao; Li; Zhou; Lin; Zhou; Wei [124].

In 2014, Lu et al. [124] prepared and studied a 4:1 inclusion complex of zinc phthalocyanine (ZnPc) with HP-β-CD ((HP-β-CD)_4_-ZnPc) to improve the PDT efficiency of ZnPc by increasing the water solubility and decreasing the aggregation in the physiological environment of ZnPc. No obvious toxicity was observed on human cervical carcinoma (HeLa) cells at a high concentration of 80 µM. The inclusion complex exhibited superior ^1^O_2_ production, intracellular ROS generation cellular uptake ability, and phototoxicity to cancer cells compared to free ZnPc. The first results are presented in Figure 9.

**Figure 10:**
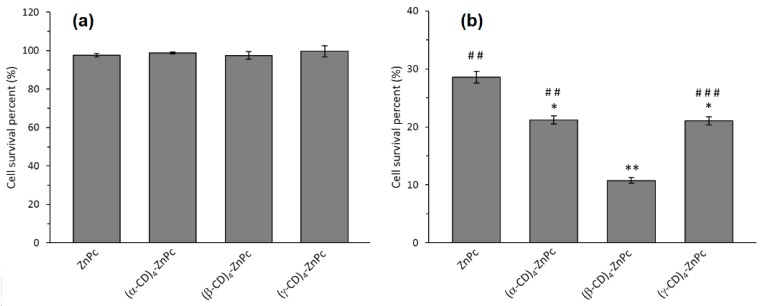
(**a**) Dark toxicity and (**b**) light toxicity of ZnPc, (α-CD)_4_-ZnPc, (β-CD)_4_-ZnPc, and (γ-CD)_4_-ZnPc on Hela cells. For dark toxicity study, the cells were incubated for 24 h, and their survival percent were studied by 3-(4,5-dimethylthiazol-2-yl)-2,5-diphenyl tetrazolium bromide (MTT) assay. For light toxicity study, after 4 h of incubation by drugs (drug concentration was 5 µM, which was calculated by ZnPc), the cells were irradiated using 665 nm LED for 5 min, and then laid back into an incubator for 24 h before cell viability measurement by MTT assay (* *p* < 0.05, ** *p* < 0.01, host-guest complex vs. ZnPc. ^##^ *p* < 0.01, ^###^ *p* < 0.001 ZnPc, (α-CD)_4_-ZnPc, and (γ-CD)_4_-ZnPc vs. (β-CD)_4_-ZnPc). Adapted from Lu; Wang; Ma; Xuan; Zhao; Li; Zhou; Zhou; Wei [125].

Two years later, the same group [125] studied the influence of the size of CD and synthesized 4:1 inclusion complexes: (α-CD)_4_-ZnPc, (β-CD)_4_-ZnPc, and (γ-CD)_4_-ZnPc. Based on extracellular ^1^O_2_ generation ability studies, (β-CD)_4_-ZnPc appeared to be the best generator of ^1^O_2_. The cellular uptake of the inclusion complexes was increased when compared with free ZnPc and particularly with (β-CD)_4_-ZnPc. They also compared the PDT efficiency of these different compounds using Hela cells, and Figure 10 shows the better in vitro PDT efficiency of the inclusion complexes compared to free ZnPc.

**Figure 11:**
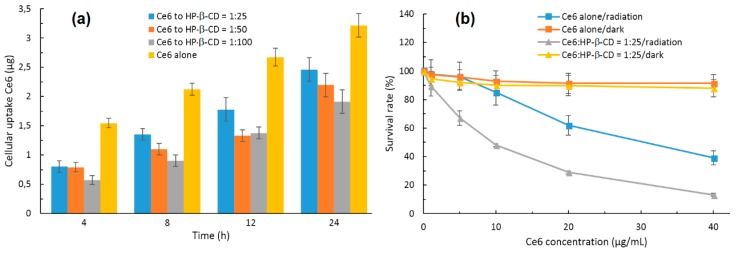
(**a**) In vitro cellular uptake of the Ce6-HP-β-CD inclusion complex by oral squamous carcinoma (OSC) cells for different Ce6 to HP-β-CD ratios and (**b**) phototoxicity of optimized inclusion complex and control formulations against OSC cells. Adapted from Paul; Heng; Chan [126].

In 2015, Paul and his coworker [126] described the incorporation of chlorin e6 (Ce6) into the HP-β-CD cavity, and a 1:1 stoichiometric ratio was found. The authors showed that the complexation of Ce6 with HP-β-CD enhanced the Ce6 solubility, decreased its aggregation in water, and enhanced its ^1^O_2_ yield at the pH tumor environment compared to the free Ce6. However, the in vitro cellular uptake of the Ce6-HP-β-CD inclusion complex performed on human oral squamous carcinoma (OSC) indicated that Ce6-HP-β-CD with a higher HP-β-CD concentration decreased their cellular uptake due to the higher viscosity of the microenvironment in the presence of a significant amount of HP-β-CD. Finally, the in vitro phototoxicity studies of the Ce6-HP-β-CD inclusion complex (Ce6:HP-β-CD = 1:25, pH 6.2, 30 mW/cm^2^ for 5 min) were performed on the OSC cells. The obtained results highlighted the important role of HP-β-CD in affecting the phototoxicity and PDT efficiency of Ce6 against tumors (Figure 11). The authors concluded that CDs derivatives of larger size such HP-β-CD could be a successful formulation excipient to deliver monomeric Ce6 with improved PDT efficiency.

**Figure 12:**
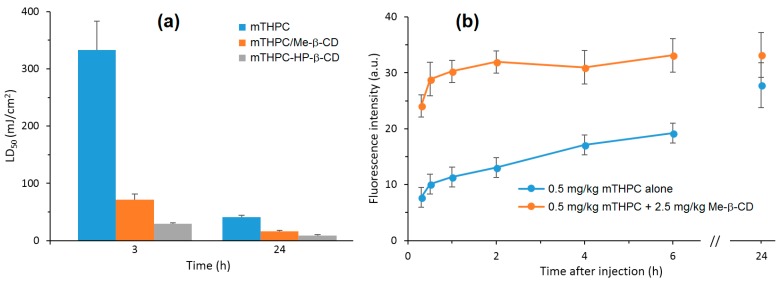
(**a**) Influence of 10 μM methyl-β-cyclodextrin (Me-β-CD) and 200 μM HP-β-CD on the photosensitivity of HT29 cells incubated in the presence of 1.47 μM meta-tetra(hydroxyphenyl)chlorin (mTHPC) over 3 h and 24 h. LD_50_ was determined in three independent experiments and expressed as the mean with the vertical bar showing SD. (**b**) Kinetics of in vivo fluorescence (λ_exc_ = 405 nm, λ_em_ = 600–700 nm) from the surface of the HT29 tumor grafted to NMRI^nu/nu^ mice after injection of 0.5 mg/kg mTHPC alone in the tail vein or with 2.5 mg/kg Me-β-CD. Results are expressed as the mean of three to four measurements with the vertical bar showing SD. Adapted from Yankovsky; Bastien; Yakavets; Khludeyev; Lassalle; Gräfe; Bezdetnaya; Zorin [127].

Recently, Yankovsky et al. [127] studied the effect of two β-CDs derivatives, i.e., methyl-β-cyclodextrin (Me-β-CD) and HP-β-CD, at a wide range of concentrations on the in vitro and in vivo distribution of meta-tetra(hydroxyphenyl)chlorin (mTHPC). The authors found that the association of mTHPC with the β-CDs prevents its aggregation after introduction into blood, and enhanced its diffusion movement between biological structures. In addition, they demonstrated that the mTHPC distribution in blood serum and its accumulation in cellular culture medium were highly dependent on the β-CDs concentrations (maximal mTHPC accumulation at 10 µM of Me-β-CD and 200 µM of HP-β-CD) (Figure 12a). Furthermore, photosensitization studies showed that the addition of β-CDs affect the intracellular distribution of mTHPC and enhance its photocytotoxicity effect toward HT29 human adenocarcinoma cultured cells inclusion complexes (Me-β-CD LD_50_: 70 mJ/cm^2^ and 14 mJ/cm^2^ at 3 h and 24 h, and in the case of HP-β-CD 27 mJ/cm^2^ and 8 mJ/cm^2^) (Figure 12a). Finally, the in vivo fluorescence kinetics studies and fluorescent imaging of the mTHPC distribution in different tissues (Figure 12b) confirm that the use of β-CDs modifies the mTHPC distribution process in tumor-bearing animals with a decreased level of mTHPC in skin and muscles, and an increased mTHPC accumulation in tumor were observed.

**Figure 13:**
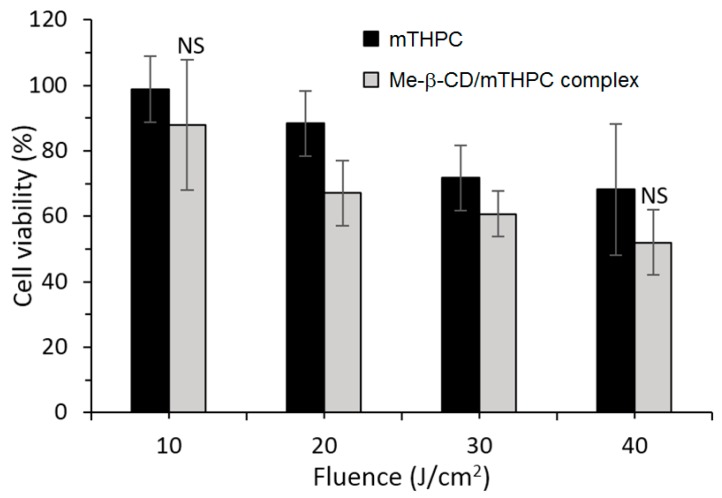
Cell viability of spheroids incubated 24 h with free mTHPC and Me-β-CD/mTHPC inclusion complex. The percentage of cell survival was counted by means of clonogenic assay 15 days after PDT. mTHPC concentration was 4.5 µM. Statistically different from mTHPC alone treatment at the given fluence, *p* < 0.05; NS: no significant differences from mTHPC alone treatment at the given fluence, *p* < 0.05. Adapted from Yakavets; Yankovsky; Millard; Lamy; Lassalle; Wiehe; Zorin; Bezdetnaya [128].

More recently, the same group published a work concerning the distribution and PDT efficiency of Me-β-CD or HP-β-CD encapsulating mTHPC on multicellular HT29 tumor spheroids [128]. The 2:1 inclusion (Me-β-CD/mTHPC or HP-β-CD/mTHPC) induced showed two and three times higher mTHPC accumulation in spheroids than mTHPC alone. The authors highlighted the different distribution of the two inclusion complexes: whereas HP-β-CD/mTHPC accumulated at the spheroid periphery, Me-β-CD/mTHPC penetrated deeper, with a more homogeneous distribution into the spheroid. At a low light dose such as 20 J/cm^2^, the Me-β-CD/mTHPC inclusion complex presented a phototoxicity that was 25% higher than mTHPC alone (Figure 13).

**Figure 14:**
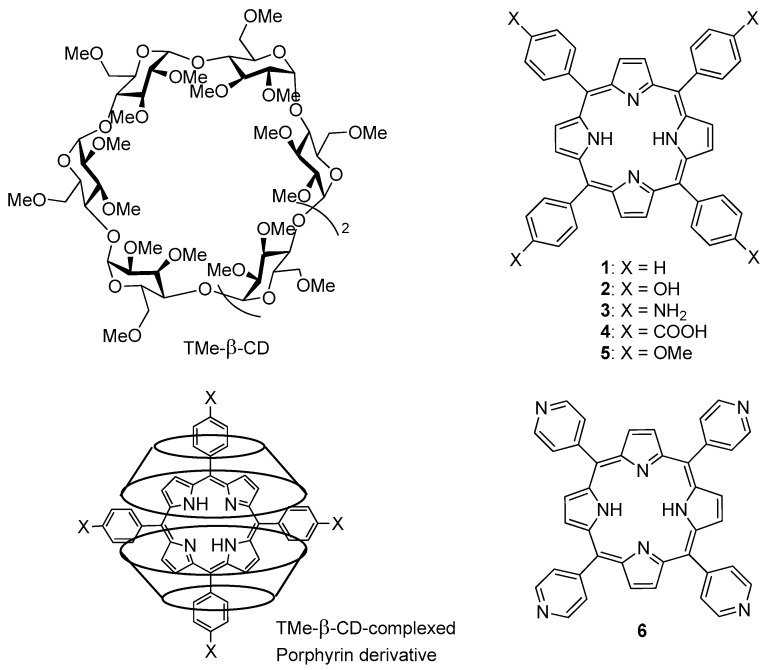
Chemical structure and schematic illustration of the trimethyl-β-CD (TMe-β-CD) complexed with porphyrin derivatives.

In 2017, Ikeda et al. [129] described the PDT effect of 2:1 inclusion complexes **1**–**6** of trimethyl-β-CD (TMe-β-CD) with different porphyrins, as described in Figure 14.

For photostability reasons and ^1^O_2_ generation ability, only **2**–**4** were selected for in vitro PDT study. Inclusion complexes **1**, **2**, and **6** were unstable under light irradiation. Inclusion complexes **2**–**4** generated ^1^O_2_ in the order **3** > **2** ≈ **4**. PDT experiments were done with the HeLa cell line after irradiation of 610–740 nm for 30 min under 9 mW/cm^2^ of light power. The order of PDT activity was **3** > **2** >> **4**, suggesting that the PDT effect is more due to the higher intracellular uptake of inclusion complexes **2** and **3** (endocytosis) than ^1^O_2_ formation. The authors could check the formation of ^1^O_2_ in Hela cells by inclusion complexes **2** and **3**, which presented 14 and 26 times higher PDT activity than Photofrin^®^.

- Non-Porphyrinoid photosensitizers

Many of the approved PDT PSs for clinical use are porphyrinoid derivatives. However, their usefulness in PDT can sometimes be limited by various factors such as relatively poor water solubility and photostability. As a consequence, major efforts are made to develop new non-porphyrinoid PSs [130].

**Figure 15:**
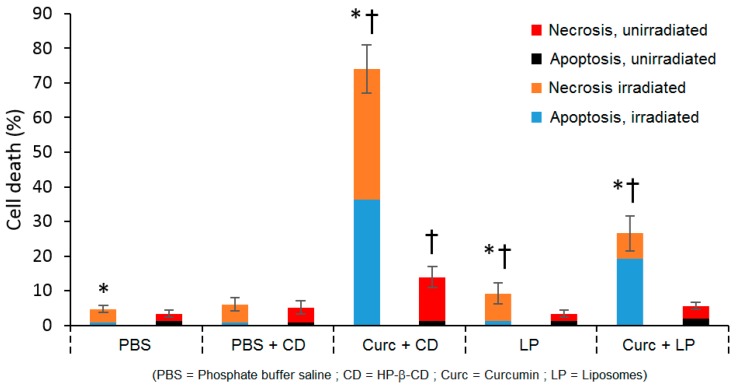
Cytotoxicity expressed as percentage of salivary gland acinar cells SM 10–12 cell death in terms of apoptosis and necrosis assayed by the PI/Hoechst staining and fluorescence technique. The cells were irradiated with blue light of a 6 J/cm^2^ beam area or kept for 5 min in the dark in the presence of 13.5 µM natural curcumin in 5% cyclodextrin (CD) or 0.4 µM in 5 µg/mL liposomes (LP). Data are means ± SEM (*n* ≥ 6). Significantly different from corresponding unirradiated samples (for phosphate buffered saline (PBS): necrosis only) (*); significantly different from irradiated vehicle in PBS and PBS only (for CD samples: also different from unirradiated vehicle) (^†^); *p* ≤ 0.05. Adapted from Bruzell; Morisbak; Tonnesen [131].

In 2005, Bruzell et al. compared the PDT efficiency of different formulations of curcumin (curc) into DMSO, non-ionic micelles liposomes (LP), HP-β-CD, or alginate viscous solution [131]. PDT efficiency was evaluated by two techniques: PI/Hoechst staining and fluorescence technique, and 3-(4,5-dimethylthiazol-2-yl)-2,5-diphenyl tetrazolium bromide (MTT) assay. By MTT assay, no effect of light could be observed after 350 nm to 550 nm of light irradiation (1.6 J/cm^2^ and 3.6 J/cm^2^ per well) of HP-β-CD/curcumin inclusion complex (5% HP-β-CD), whereas curcumin in HP-β-CD increased the apoptotic SM10-12 cells by 20-40-fold compared to non-irradiated cells or irradiated cells without curcumin (Figure 15).

As a conclusion, except for viscous alginate solution, all of the formulations were suitable for using curcumin in damaging submandibular acinar cells. The phototoxic effect can be detected at low curcumin concentration (13.5 µM) with low light doses (1.6 J/cm^2^ and 3.6 J/cm^2^ per well) and a short incubation time (3 h). Unfortunately, the authors did not continue their study on anticancer PDT, and focused their efforts on antimicrobial PDT (see Valeron Bergh; Hjorth Tonnesen. [113] and references cited therein.)

**Figure 16:**
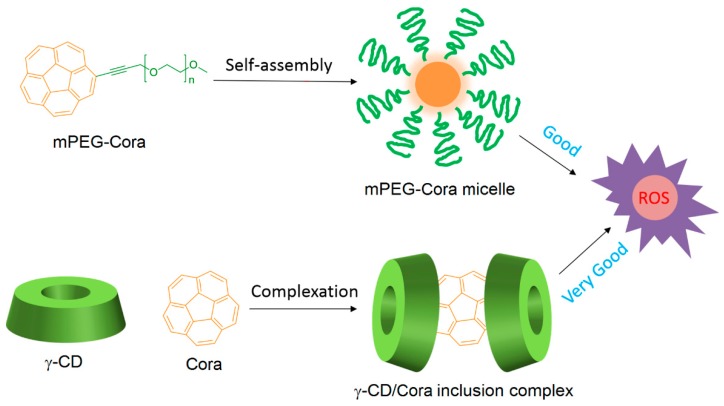
Two types of Corannulene (Cora) solubilization vehicles. The γ-CD/Cora inclusion complex is proposed to have a better ability for apparent ROS generation compared to the self-assembling methoxy poly(ethylene glycol)-corannulene (mPEG-Cora) micelle. Adapted from Zhang; Dong; Lu; Liu; Ding; Kong; Fan; Wang; Zhao [132].

The non-porphyrinoid PS Corannulene (Cora) is known to produce ROS in a controlled manner, but the use of Cora is limited because of its low water solubility. Very recently, Zhang et al. [132] designed two types of water-soluble Cora systems, i.e., methoxy poly(ethylene glycol)-corannulene (mPEG-Cora) micelle and a γ-CD/Cora inclusion complex (Figure 16).

Subsequently, the vehicle effect on the ROS production by Cora contained in mPEG-Cora and γ-CD/Cora systems at the cellular level was studied using confocal laser scanning microscopy (365 nm, 95 mW/cm^2^, 20 min). It was found that both systems can produce ROS, but the γ-CD/Cora inclusion complex was the most effective system. 

**Figure 17:**
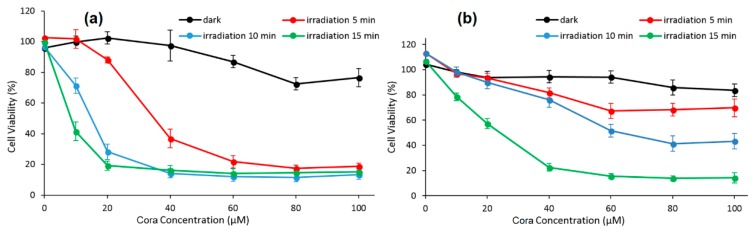
The dose-dependent viability of PC-3 cells in response to (**a**) γ-CD/Cora inclusion complex and (**b**) mPEG-Cora micelle in the absence or presence of light irradiation (365 nm, 95 mW/cm^2^). Three irradiation times were set at 5 min, 10 min, and 15 min (*n* = 3). Adapted from Zhang; Dong; Lu; Liu; Ding; Kong; Fan; Wang; Zhao [132].

The PDT effect (type I, 365 nm, 95 mW/cm^2^ for 5 min, 10 min, and 15 min) of each system on PC-3 cells were studied. At the same irradiation circumstance and Cora dose, the authors found that γ-CD/Cora inclusion complex could induce a higher extent of photocytotoxicity (e.g., for 15 min of irradiation, IC_50_ of γ-CD/Cora = 9.2 ± 4.7 μM, IC_50_ of m-PEG-Cora = 22.5 ± 2.6 μM), indicating more satisfactory therapeutic outcomes (Figure 17).

Finally, the vehicles’ effect on the mitochondria targeting behavior of Cora was studied on PC-3 cells (365 nm, 95 mM/cm^2^, 20 min). It was found that the γ-CD/Cora complex has shown superior ability to deliver more Cora to mitochondria compared with the m-PEG-Cora micelle. The authors concluded that the CD complexation approach prevailed over the PEGylation method for PDT applications.

#### 2.2.2. Cyclodextrin–Photosensitizer Conjugates

A second possible way to improve the photophysical properties of PSs for an anticancer PDT application would be to bind the CD and PS by a covalent link, i.e., the formation of a CD–PS conjugate. This binding mode has shown its potential validity for anticancer PDT application in various studies. This part of the review focuses on the in vitro or/and in vitro biological effect of CD–PS conjugates in anticancer PDT. However, with respect to articles involving the potential use of CD–PS conjugates in anticancer PDT (without in vitro and/or in vivo biological studies), we can emphasize the use of CD dimers as potential carriers with a ^1^O_2-_responsive linker that would allow either the PS release [133,134,135] or the PS concentration in the light beam and water solubility [136]. In addition, the conjugation of β-CD with a PS via a non-cleavable ether bond showed an improvement of the water solubility and in vitro fluorescent intensity of PS [137]. Other CD–PS conjugates with a dithienylethene linker have an enhanced water solubility and biocompatibility of PS, while also resulting in photo-controlled ^1^O_2_ generation in aqueous solution [138]. The studies that have presented an in vitro and/or in vivo biological anticancer PDT evaluation of CD–PS conjugates are summarized below.

- Porphyrinoid photosensitizers

**Figure 18:**
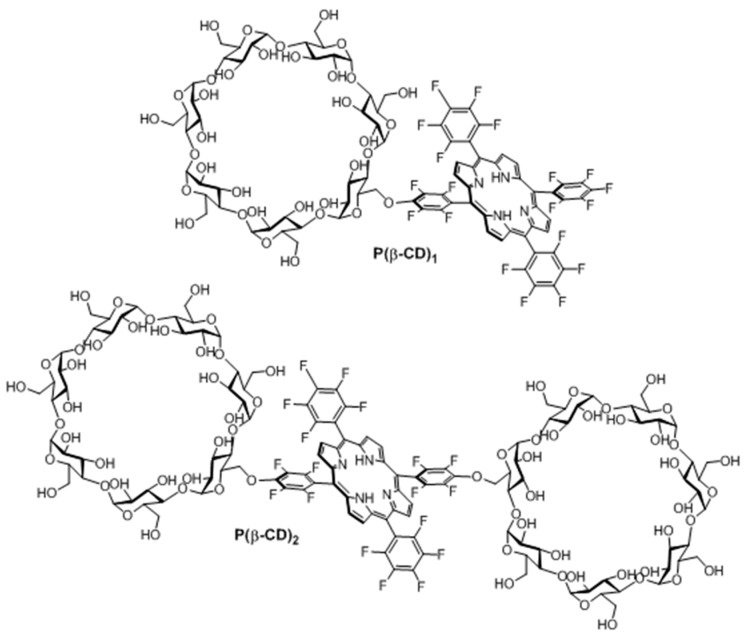
Chemical structure of P(β-CD)_1_ and P(β-CD)_2_.

In 2006, Králová et al. [139] synthesized two new perfluorinated porphyrin derivatives conjugated to one or two β-CD units (P(β-CD)_1_ and P(β-CD)_2_, as shown in Figure 18).

With the aim of estimating the pharmacokinetic and photosensitizing properties of P(β-CD)_1_ and P(β-CD)_2_, in vitro studies were performed on different cell lines, i.e., human promyelotic leukemia (HL-60), mouse mammary carcinoma (4T1), mouse colon carcinoma (CT26.CL25), and human cervical carcinoma (HeLa). The phototoxicity of P(β-CD)_1_ and P(β-CD)_2_ at concentrations of 5 µM and 10 µM were investigated with HL-60 and 4T1 cells under irradiation at various doses of light (0 to 4.2 J/cm^2^). No dark cytotoxicity was detected; nevertheless, an increased cell death was seen in both cell lines when concentration and light dose increased. It was found that P(β-CD)_2_ was less efficient compared to P(β-CD)_1_. A similar effect was observed with the other cell lines (CT26.CL25 and HeLa), along with an increase in cell death over time. Furthermore, the accumulation in the tumor is higher and faster for P(β-CD)_2_.

**Figure 19:**
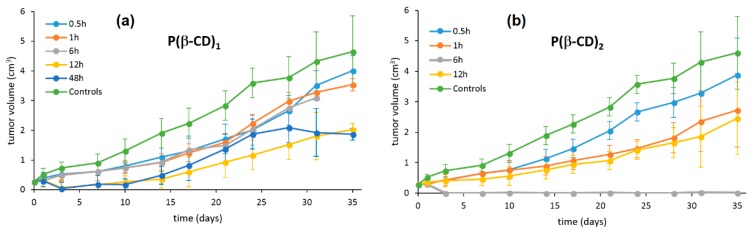
Effects of (**a**) P(β-CD)_l_- and (**b**) P(β-CD)_2_-mediated PDT on tumor growth. BALB/c mice bearing subcutaneously growing 4T1 mammary carcinoma (*n* = six per each group) received a single dose of the drug (5 mg/kg) and were then irradiated (100 J/cm^2^, 200 mW/cm^2^) at indicated time points. The control group consisted of untreated tumor bearing mice. Adapted from Králová; Synytsya; Pouckova; Koc; Dvorak; Kral [139].

Finally, in vivo studies were realized using a 4T1 mouse-tumor model. P(β-CD)_1_ and P(β-CD)_2_ were injected into mice (5 mg/kg) and then exposed to light illumination (100 J/cm^2^) at various times between 0.5 h and 35 h after injection. It was found that P(β-CD)_2_ was the most efficient at totally inhibiting the tumor growth for a drug-light interval of 6 h (Figure 19).

**Figure 20:**
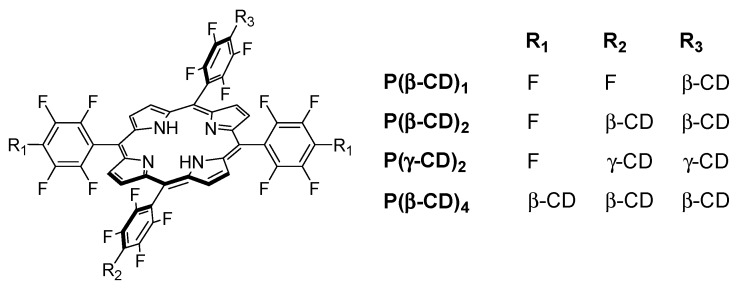
Chemical structure of porphyrin–CD conjugates (P(CD)_x_).

In 2010, the same team synthesized a new Lego-like system composed of perfluorinated porphyrin–CD conjugates (P(CD)_x_) and various chemotherapy drugs [140]. The perfluorinated porphyrins were first conjugated to CDs (β-CD or γ-CD) by covalent ether bond (Figure 20) using the same strategy as described in their previous study [139]. The chemotherapy drugs were then encapsulated into the cavity of CDs by non-covalent bond, i.e., inclusion complexes.

Due to the low affinity of P(β-CD)_1_ conjugate with the various chemotherapy drugs, the authors only investigated P(β-CD)_2_, P(γ-CD)_2_, and P(β-CD)_4_ conjugates for in vitro and in vivo studies using mouse mammary carcinoma (4T1) and human chronic myelogenous leukemia (K562) cell lines. The dark cytotoxicity and phototoxicity (λ = 500–520 nm, 4 J/cm^2^, 0.7 mW/cm^2^) of cells treated with chemotherapy drugs alone or their corresponding inclusion complexes with (P(CD)_x_ conjugates were compared. They found that compared to the non-irradiated cells, the irradiated ones were much efficient in cancer cell killing. Furthermore, the authors observed a synergistic effect between PDT and a large fraction of chemotherapy drugs compared to chemotherapy or PDT alone. 

Finally, in vivo studies were also done in a mouse cancer model. The P(β-CD)_2_, P(γ-CD)_2_, and P(β-CD)_4_ conjugates or their corresponding inclusion complexes with two chemotherapy drug models (doxorubicin and paclitaxel) were injected to the mouse followed by light irradiation (100 J/cm^2^, 200 mW/cm^2^). The highest decrease of tumor growth was observed for inclusion complexes of P(β-CD)_2_ and P(γ-CD)_2_ with chemotherapy drugs (paclitaxel and doxorubicin, respectively). These results are in agreement with those of in vitro studies indicating the usefulness of the synthesized conjugates for both targeted chemotherapy drug delivery and combined cancer therapy.

**Figure 21:**
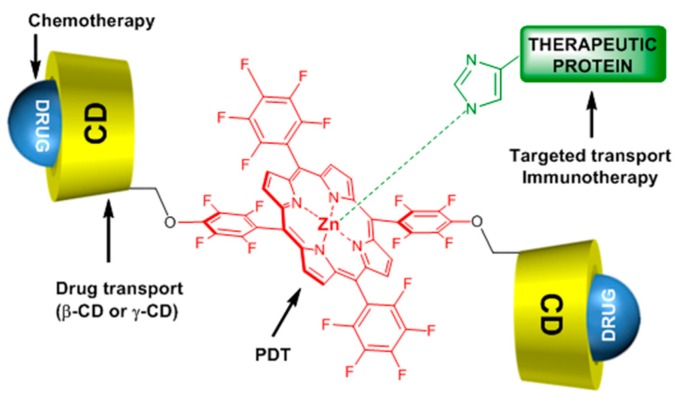
Schematic representation of Lego-like systems (including three parts: therapeutic protein, ZnP(β-CD)_2_ or ZnP(γ-CD)_2_ conjugates, and a chemotherapy drug) for targeted and combined therapy. Adapted from Kejik; Briza; Kralova; Pouckova; Kral; Martasek; Kral [141].

One year after and being encouraged by the results obtained in the last study [140], the same team developed another “Lego”-like system based on a combination of therapeutic protein, metallo-cyclodextrin-porphyrin, and chemotherapy drugs in order to enhance the inhibition of tumor progress [141]. The authors used the previous P(β-CD)_2_ and P(γ-CD)_2_ conjugates with the encapsulation of chemotherapy drugs (paclitaxel, doxorubicin), but this time by metalating the porphyrin core with Zn to coordinate therapeutic proteins (Figure 21).

**Figure 22:**
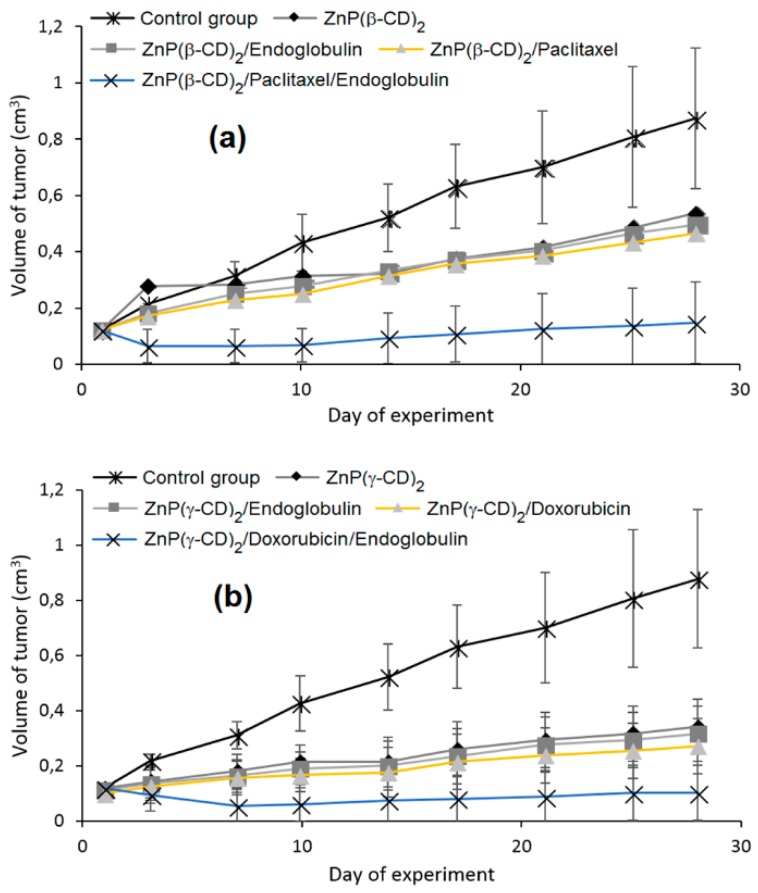
Effect of combined therapy with ZnP(β-CD)_2_ or ZnP(γ-CD)_2_ conjugates, chemotherapy drugs (paclitaxel or doxorubicin), and endoglobulin on the tumor volume of human amelanotic melanoma C32 in an in vivo nude mouse model. (**a**) ZnP(β-CD)_2_/paclitaxel/endoglobulin or (**b**) ZnP(γ-CD)_2_/doxorubicin/endoglobulin (*n* = 6 at each group). Adapted from Kejik; Briza; Kralova; Pouckova; Kral; Martasek; Kral [141].

The authors prepared two types of Lego-like systems based on ZnP(β-CD)_2_/paclitaxel/endoglobulin or ZnP(γ-CD)_2_/doxorubicin/endoglobulin. The effect of combined therapy with both systems on the tumor volume of human amelanotic melanoma C32 in the in vivo nude mouse was studied. They found that compared to the ZnP(β-CD)_2_/paclitaxel/endoglobulin system, the ZnP(γ-CD)_2_/doxorubicin/endoglobulin system was most efficient in the case of PDT treatment alone (λ = 500–700 nm, 100 J/cm^2^, 200 mW/cm^2^) or combined therapy. Moreover, they found that by combining ZnP(β-CD)_2_ or ZnP(γ-CD)_2_ conjugates, the chemotherapy drug and endoglobulin enhanced the tumor destruction (Figure 22). 

**Figure 23:**
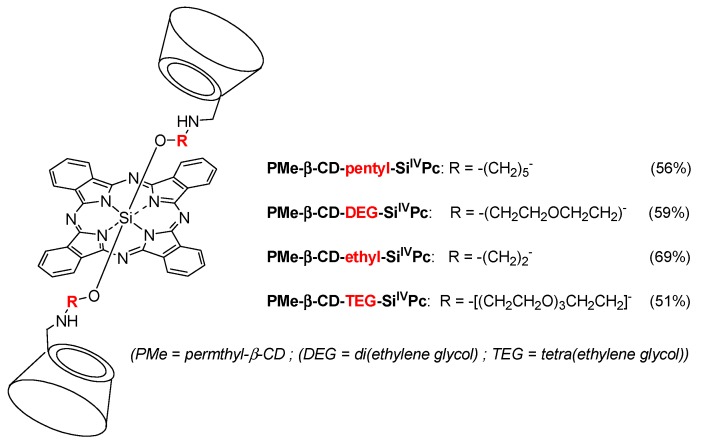
Chemical structure of PMe-β-CD-R-Si^IV^Pc conjugates.

In 2011, Ng et al. designed a new series of analogous complexes [142,143]. In the first study [142], they studied the influence of the linker’s size (Figure 23) on the photophysical properties and in vitro PDT activity.

All of the conjugates, except PMe-β-CD-ethyl-Si^IV^Pc due to a photoinduced electron transfer (PET) process, have the ability to greatly enhance the water solubility of the phtalocyanine core and reduce its self-aggregation in water. In vitro PDT activity using human colon adenocarcinoma (HT29) and human hepatocarcinoma (HepG2) cells was evaluated and summarized in Table 4.

**Figure 24:**
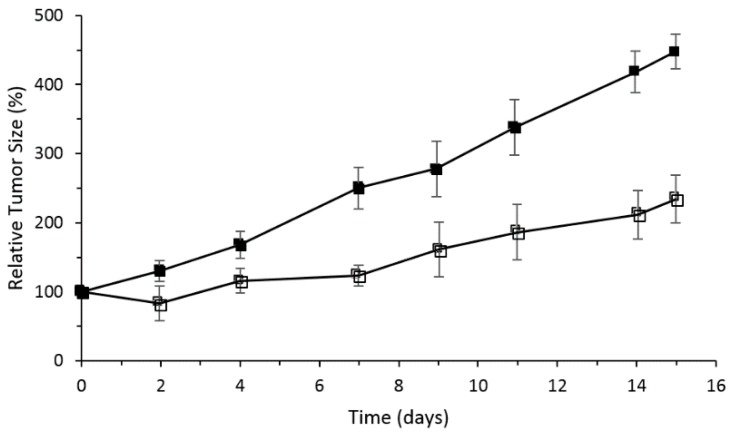
Tumor growth delay after PDT treatment with PMe-β-CD-hexyl-Si^IV^Pc under illumination (λ = 635 nm, 30 J/cm^2^) (□) and mice kept in darkness for control (■). Adapted from Lau; Lo; Fong; Ng [142].

The PMe-β-CD-hexyl-Si^IV^Pc conjugate showed the best photocytotoxicity, which was explained by the difference in ROS production efficiency and cellular uptake by the lysosomes of the cells. It was shown by flow cytometry that cells in the early apoptotic state increase to 80% upon red light illumination. In vivo PDT activity was performed using nude mice with an HT29 tumor, PMe-β-CD-hexyl-Si^IV^Pc, and irradiation at 675 nm (30 J/cm^2^). Figure 24 shows the relative tumor size for 15 days, and clearly indicates that the PMe-β-CD-hexyl-Si^IV^Pc conjugate is a promising system for anticancer PDT.

**Figure 25:**
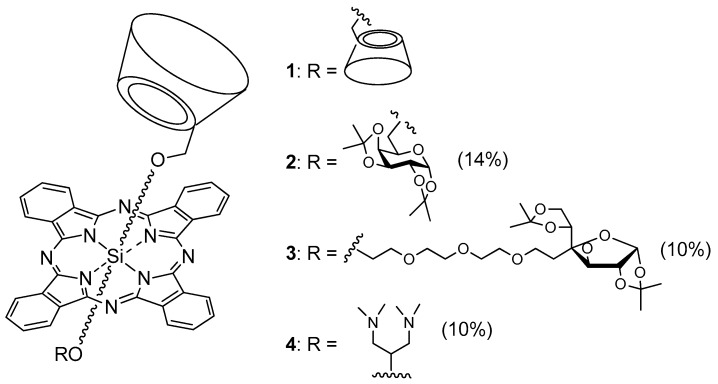
Chemical structure of PMe-β-CD-Si^IV^Pc conjugates **1**–**4**.

In their second study [143], the authors Lau; Lo; Fong; Ng reported the synthesis of unsymmetrical PMe-β-CD-Si^IV^Pc conjugates using Leng’s procedure [122] (Figure 25). The Q-band was sharp for PMe-β-CD-hexyl-Si^IV^Pc conjugates **1** and **4**, and significantly broadened for PMe-β-CD-hexyl-Si^IV^Pc conjugates **2** and **3**, suggesting that the sugar units are less effective at reducing the aggregation.

The in vitro studies were performed on HT29 and HepG2 cells with PMe-β-CD-Si^IV^Pc conjugates **1**–**4**. No dark cytotoxicity was observed for all of the compounds, but high cytotoxicity upon illumination (λ = 610 nm, 40 mW/cm^2^, 48 J/cm^2^) was highlighted. It was found also that the replacement of CD moieties in **1** by sugar or diamino groups enhance greatly the phototoxicity of the conjugates. The IC_50_ value comparison of conjugates **1**–**4** against HT29 and HepG2 cells are summarized in Table 5.

The in vivo study was also performed by injection of PMe-β-CD-Si^IV^Pc conjugate **2** in the nude mice bearing (HT29) tumor using the same protocol conditions as described in Lau; Lo; Fong; Ng [142]. Similarly, the same PDT effect was observed, indicating that the PMe-β-CD-hexyl-Si^IV^Pc conjugate **2** is a promising system for anticancer PDT.

In 2013, Aggelidou et al. [144] described a new bimodal conjugate constituted of protoporphyrin IX (PpIX) covalently linked through amide bond to β-CD (PpIX+β-CD). Spectroscopy studies were used to confirm the formation of two conjugates (PpIX-β-CD “Major” and PpIX-2β-CD “Minor”). The photophysical studies of PpIX alone compared to conjugates revealed that the presence of β-CD in the conjugates enhanced the water solubility of PpIX. Furthermore, the authors found also that both conjugates have the ability to host an anticancer drug (*N*-desmethyltamoxifen, NDMTAM.HCl) by its complexation in the empty cavity of β-CD, which shows that PpIX+βCD could efficaciously solubilize and transport NDMTAM.

**Figure 26:**
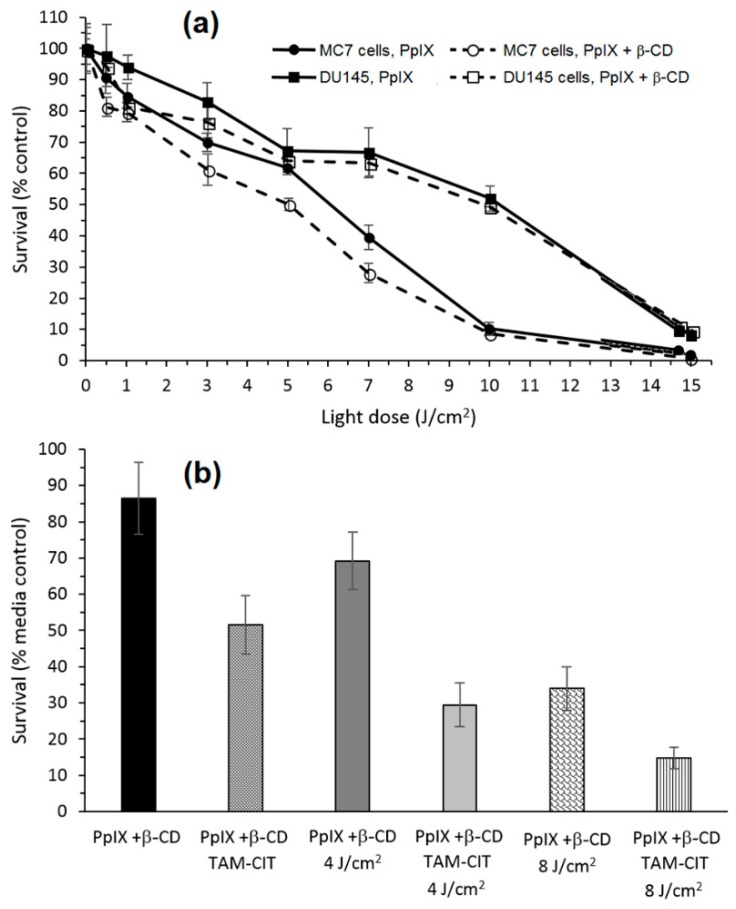
(**a**) Phototoxicity 24 h following 3 h of cell incubation with 7 µM of protoporphyrin IX (PpIX) and PpIX+β-CD and irradiation through a Schott RG610 long-pass filter (LD_50_ values for PpIX were 10 J/cm^2^ and 7 J/cm^2^ for MCF7 and DU145 cells, respectively, whereas the corresponding values for PpIX+β-CD were 10 J/cm^2^ and 6 J/cm^2^, respectively). (**b**) Bimodal action of PpIX+β-CD complexed with tamoxifen citrate (TAM-CIT) in MCF7 cells. Phototoxicity 48 h following 3 h of cell incubation with 7 µM of PpIX+β-CD and PpIX+β-CD complexed with TAM-CIT and irradiation through a Schott RG610 long-pass filter at 4 J/cm^2^ and 8 J/cm^2^. Adapted from Aggelidou; Theodossiou; Yannakopoulou [144].

The in vitro phototoxic properties of PpIX+β-CD compared to PpIX alone were performed on human prostate carcinoma (DU145) and breast adenocarcinoma (MCF7) cell lines. PpIX+β-CD displayed less toxicity in the dark than PpIX (15% against 25%, respectively) in both cell lines, which could be explained by β-CD improving the solubility of PpIX, and thus reducing its ability to aggregate. The in vitro evaluation of the phototoxicity depending of the light dose (λ = 610 nm, 0 to 15 J/cm^2^, 15 mW/cm^2^) is given in Figure 26a. There were no significant differences between PpIX and PpIX+β-CD, indicating that β-CD had no impact on the phototoxicity properties of PpIX.

Finally, the ability of PpIX+β-CD conjugates to host and transport a therapeutic molecule was investigated using NDMTAM. The PpIX+β-CD seemed to be a good drug carrier for the intracellular transport of this drug. Finally, the bimodal action of PpIX+β-CD complexed to tamoxifen citrate (TAM-CIT) was investigated using the MCF7 cell line (Figure 26b). Under an irradiation of 4 J/cm^2^, the PpIX+β-CD complexed to TAM-CIT had a toxicity of 70% against 30% for PpIX+β-CD alone, and under 8 J/cm^2^, the toxicity was 85% against 67%. These observations highlighted that there was a synergistic effect of the toxicity of the chemotherapy drug and the phototoxicity of the PpIX+β-CD conjugates. 

**Figure 27:**
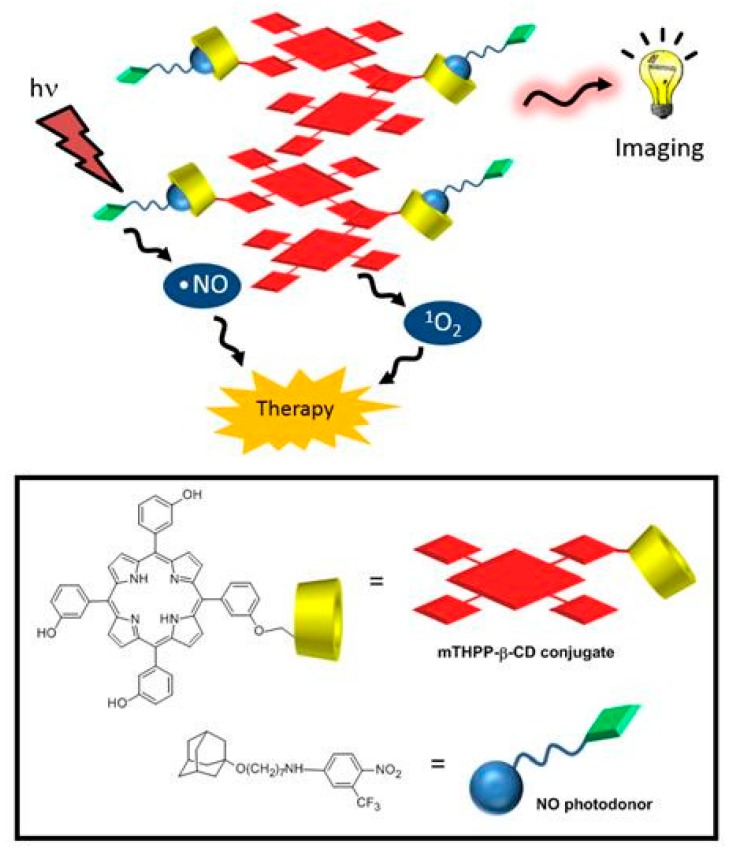
Schematic representation of the photoresponsive supramolecular nanoaggregate. Adapted from Fraix; Goncalves; Cardile; Graziano; Theodossiou; Yannakopoulou; Sortino [145].

In the same year, Fraix et al. [145] synthesized and investigated the properties of a new supramolecular assembly as a bimodal agent for PDT and imaging composed of *meta*-(3-hydroxyphenyl)-porphyrin (mTHPP) conjugated to β-CD (mTHPP-β-CD conjugate) by an ether bond, and a nitric oxide photodonor (NO phodonor) tailored to fit the β-CD cavity (Figure 27).

They found that compared to the free mTHPP, the mTHPP-β-CD conjugate has shown less aggregation in an aqueous environment, and formed small nanoassemblies with diameters of around 13 nm. Furthermore, the empty cavity of β-CD in the mTHPP-β-CD conjugate also had the ability to a host a nitroaniline derivative (NO photodonor) while maintaining the nanometer character of the aggregate and the fluorescence of the porphyrin core. In addition, bichromophoric nanoassemblies (β-CD-mTHPP/NO photodonor) were found to be able to generate both NO and ^1^O_2_ upon excitation with visible light (λ_exc_ > 400 nm).

**Figure 28:**
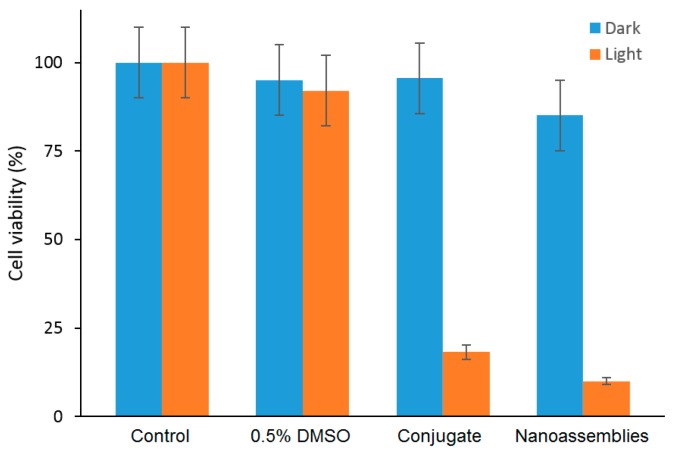
Dark and photoinduced mortality of A375 cells treated with *meta*-(3-hydroxyphenyl)-porphyrin (mTHPP)-β-CD conjugate and mTHPP-β-CD/nitric oxide (NO) photodonor assemblies (ca. 8 mm) in culture medium containing 0.5% DMSO compared to dark and photoinduced mortality in culture medium in the absence and the presence of 0.5% DMSO. Adapted from Fraix; Goncalves; Cardile; Graziano; Theodossiou; Yannakopoulou; Sortino [145].

By in vitro studies, the internalization in a human amelanotic melanoma cell line (A375) was evaluated for the conjugate and the bichromophoric nanaoassemblies at a concentration of 8 µM after 4 h of incubation, and the authors found a localization mainly in cytoplasm. The in vitro ability of the conjugate and the bichromophoric nanaoassemblies to induce A375 cell mortality upon light irradiation was compared. No dark toxicity was found, but a high phototoxicity was observed for both compounds, with a slight difference between them explained by the liberation of NO and ^1^O_2_ for the nanoassemblies (Figure 28).

**Figure 29:**
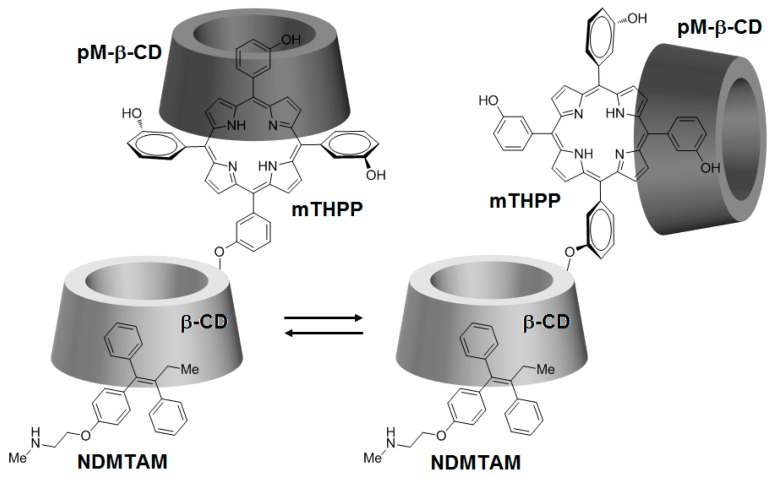
Solution structure of mTHPP-β-CD/pM-β-CD/*N*-desmethyltamoxifen (NDMTAM) as a 1:1 mixture of diastereoisomeric complexes, as derived from NMR spectroscopic data. Adapted from Theodossiou; Goncalves; Yannakopoulou; Skarpen; Berg [146].

Being encouraged by the potential properties of the mTHPP-βCD conjugate, recently, the same team [146] investigated the photochemical internalization (PCI) potential of the same conjugate with the NDMTAM drug as the guest molecule. In order to improve the water solubility of the mTHPP core in the conjugate, the authors encapsulated the mTHPP core in the empty cavity of heptakis(2,3,6-*O*-methyl)-β-CD (pM-β-CD), leading to the formation of a new mTHPP-β-CD/pM-β-CD nanosystem. The obtained nanosystem has shown the ability to host the NDMTAM drug through either one of its unsubstituted phenyl groups (Figure 29).

**Figure 30:**
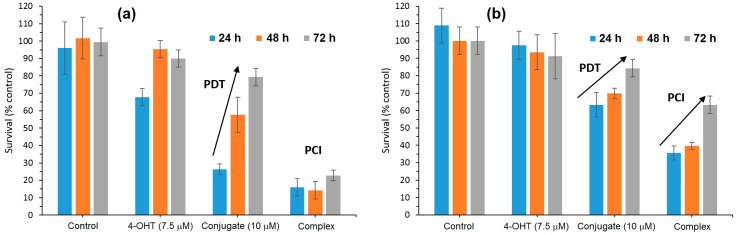
Photocytotoxicity to (**a**) breast adenocarcinoma (MCF7) and (**b**) breast human carcinoma (MDA-MB-231) cells following overnight (16 h) incubation with medium only (Control), tamoxifen (4-OHT) (7.5 µM), mTHPP-β-CD conjugate (10 µM), and mTHPP-β-CD(10 µM)-4-OHT(7.5 µM) complex. Adapted from Theodossiou; Goncalves; Yannakopoulou; Skarpen; Berg [146].

According to confocal microscopy studies, it was found that under irradiation, the porphyrin core of mTHPP-β-CD expedited endosomal membrane rupture and NDMTAM release into the cytosol. Furthermore, the authors also investigated the in vitro photocytotoxicity efficiency on breast human carcinoma (MDA-MB-231) and MCF7 cell lines of the mTHPP-β-CD conjugate, tamoxifen (4-OHT), and the resulting mTHPP-β-CD/4-OHT complex. Upon irradiation at LD_50_ light doses, in the case of cells treated with the conjugate, phototoxicity around 70% was observed, which was annulled after 48 h and 72 h. Concerning cells irradiated after treatment with the complex, they showed a cell death of 80%, with no change even after 48 h and 72 h (Figure 30).

**Figure 31:**
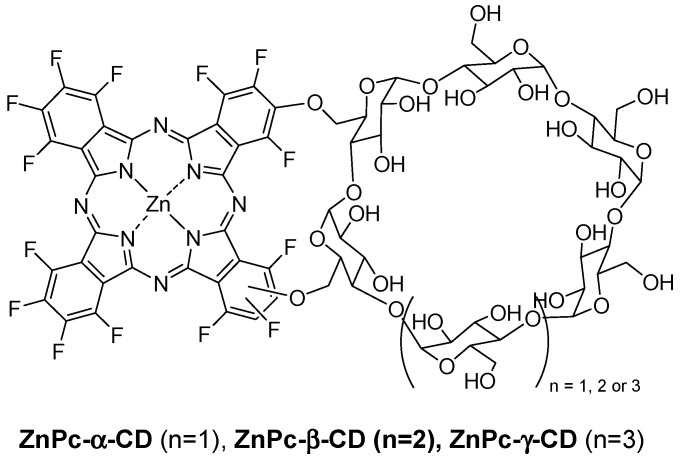
Chemical structure of ZnPc-α-CD, ZnPc-β-CD, and ZnPc-γ-CD conjugates.

In 2014, Lourenço et al. described the synthesis and PDT properties of CD–PS conjugates (ZnPc-α-CD, ZnPc-β-CD, and ZnPc-γ-CD) constituted by a zinc perfluorinated phthalocyanine (ZnPc) covalently linked through ether bonds to various types of CDs (α-CD, β-CD, and γ-CD) (Figure 31) [147].

It was found that ZnPc-α-CD and ZnPc-γ-CD have a better solubility compared to ZnPc-β-CD due to the lesser solubility of the β-CD unity itself in water compared to α-CD and γ-CD. The ability of each ZnPc–CD conjugate to generate ^1^O_2_ was evaluated using 1,3-diphenylisobenzofuran as a probe. The authors found a similar effectiveness for ZnPc-α-CD and ZnPc-γ-CD conjugates compared to ZnPc alone, but ZnPc-β-CD seemed to be less effective, which was maybe due to the same reasons mentioned before. 

The human cancer cell line derived from the transitional cell carcinoma of the bladder (UM-UC-3) was selected as a cancer cell model to study the in vitro photosensitizing efficiency. No dark toxicity was observed for the different conjugates at concentrations up to 10 µM and 4 h of incubation time. Under light irradiation (white or red-light source, 50 mW/cm^2^), after incubation with various concentrations of conjugates (0–1 µM), only ZnPc-α-CD and ZnPc-γ-CD conjugates have shown a phototoxic effect, and this effect was irradiation time and concentration-dependent. In addition, this effect was found to be more efficient under white-light irradiation compared to the red one. To better understand these results, the authors investigated the ROS production capacity of the conjugates under white or red-light irradiation using a 2′,7′-dichlorodihydrofluorescein diacetate (DCFDA) probe. They found that all of the conjugates produced ROS under white or red irradiation, and intracellular ROS production was higher in the case of ZnPc-α-CD and ZnPc-γ-CD conjugates. Indeed, they accumulated more in cells than ZnPc-β-CD, proving again their higher potential compared to ZnPc-β-CD for PDT.

**Figure 32:**
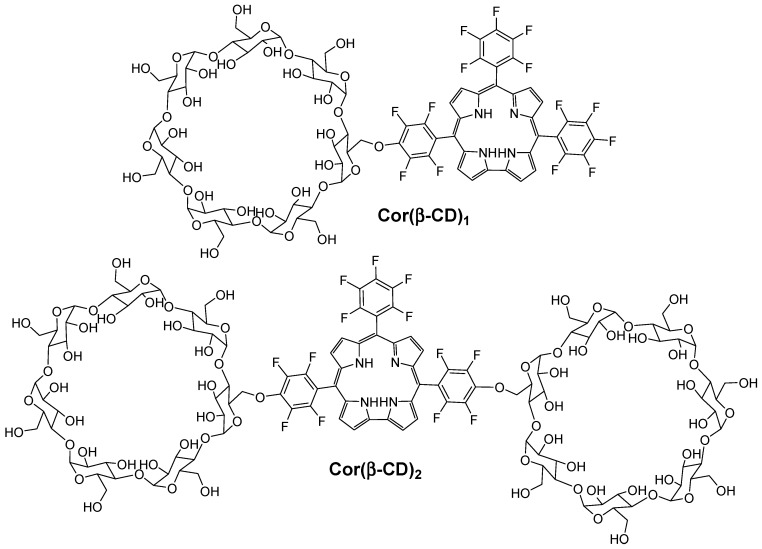
Chemical structure of Cor(β-CD)_1_ and Cor(β-CD)_2_ conjugates.

Recently, Barata et al. [148] described and investigated the photosensitizing properties of corrole β-cyclodextrin conjugates (Cor(β-CD)_1_ and Cor(β-CD)_2_), which were composed of 5,10,15-tris(pentafluorophenyl)corrole (Cor) and one or two β-CD units (Figure 32).

The authors found that the presence of β-CD units in both conjugates improved the hydrophicility of the corrole core. It was found also that all of the conjugates are highly photostable and able to produce ^1^O_2_. 

In vitro studies were performed on human cervical cancer cell line (HeLa). The cytotoxicity of each compound (Cor(β-CD)_1_, Cor(β-CD)_2_, and Cor) was evaluated by using MTT assay at different concentrations (from 10^−7^ M to 10^−4^ M) in the dark. No cytotoxicity was found for both Cor and Cor(β-CD)_1_ conjugate in contrast to the Cor(β-CD)_2_ conjugate, which conduced to 14% of cytotoxicity in cells. Upon red-light irradiation (3 J/cm^2^, 6 J/cm^2^, 9 J/cm^2^, and 12 J/cm^2^, 5 mW/cm^2^), all of the compounds showed concentration-dependent and light-dose dependent cell-destruction ability. Therefore, as shown in Table 6, Cor seems to be the most effective compound to be used as a PS for PDT.

Due to the dark cytotoxicity of the Cor(β-CD)_2_ conjugate, the authors selected only Cor and Cor(β-CD)_1_ compounds for the next studies. According to the subcellular localization study, Cor was found to be mainly accumulated in lysosomes, whereas Cor(β-CD)_1_ conjugate accumulated in the Golgi apparatus. Finally, they investigated the PDT effect of Cor and Cor(β-CD)_1_ compounds on the cytoskeleton by combination of the injection of Cor or Cor(β-CD)_1_ compounds (10^−5^ M) upon red-light irradiation (12 J/cm^2^) on microtubules in HeLa cells. They observed changes on microtubules for both Cor and Cor(β-CD)_1_ compounds, but a higher PDT efficiency was observed in the case of Cor, which may be due to its localization in lysosomes.

- Non-Porphyrinoid photosensitizers

**Figure 33:**
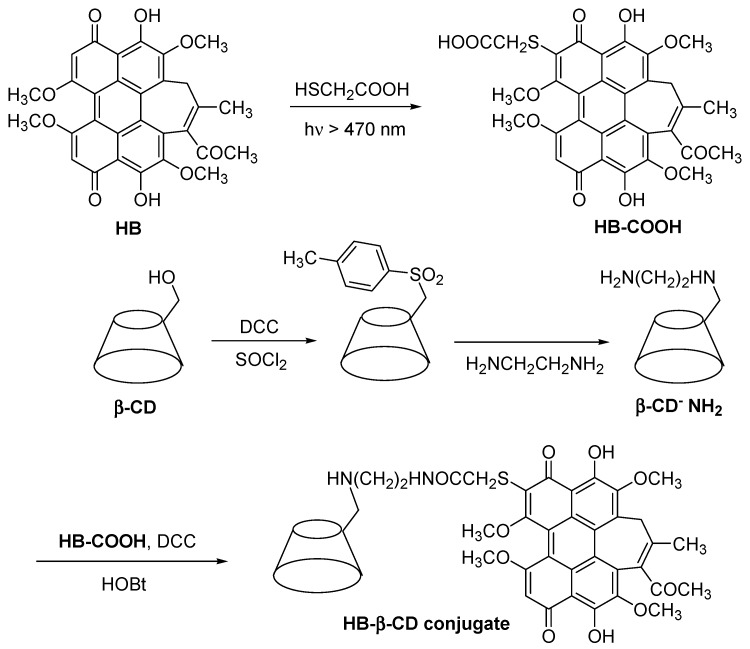
Synthesis of HB-β-CD conjugate.

In 2002, Ou et al. [149] synthesized a modified non-porphyrinoid PS (hypocrellin B, HB) conjugated to the β-CD (HB-β-CD conjugate) (Figure 33), and studied its water solubility and PDT properties. They found that the HB-β-CD conjugate had a higher water solubility compared to free HB. They also proved that the HB-β-CD conjugate can produce different ROS such as O_2_**^•−^**, **^•^**OH, and ^1^O_2_ species. Furthermore, the authors found that the β-CD unit present in the HB-β-CD conjugate reinforced their affinity for calf thymus DNA (CT DNA), leading to the stronger photodamage of CT DNA compared to free HB. The authors concluded that the introduction of β-CD units enhanced the water solubility and PDT properties of HB. 

**Figure 34:**
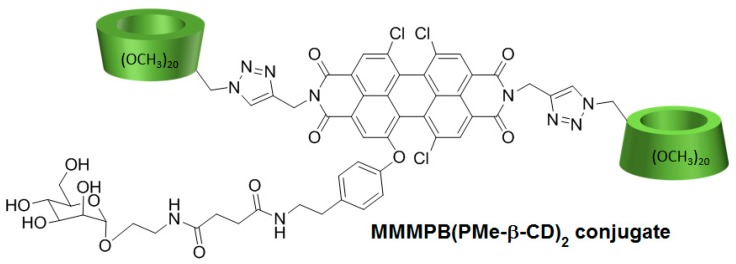
Chemical structure of mono-mannose modified perylene bisimide (MMMPB)/(PMe-β-CD)_2_ conjugate.

In 2017, Cao et al. [150] developed a new PDT agent (MMMPB(PMe-β-CD)_2_ conjugate) by conjugation of a non-porphyrinoid PS (a mono-mannose modified perylene bisimide, MMMPB) to two permethyl β-CDs (PMe-β-CDs) via click triazole links (Figure 34), and evaluated its PDT properties.

In vitro studies were performed to investigate the PDT activity of the MMMPB(PMe-β-CD)_2_ conjugate using four cell lines (A549, Hela, MCF-7, and Hep G2) at various concentrations of MMMPB(PMe-β-CD)_2_ conjugate, TPPS_4_, and cisplatin as controls (0.2 µM, 1 µM, 5 µM, 25 µM, and 100 µM). The conjugate showed no dark toxicity, but was highly phototoxic under irradiation (20 mW/cm^2^) with better IC_50_ than controls. The ability to produce ^1^O_2_ was evaluated by using 1,3-diphenylisobenzofuran as a trap, and the production of ^1^O_2_ was highlighted. Fluorescence imaging was performed in MCF-7 cells by incubation with the conjugate (10.0 µM) to localize the distribution of the conjugate in cells, and it was found mainly in cytoplasm. They finally found that the cell death was induced with an apoptotic pathway.

#### 2.2.3. Cyclodextrins-Photosensitizer Nanoassemblies

The last type of binding between CD and PS refers to CD–PS nanoassemblies. These nanoassemblies are supramolecular colloidal systems involving non-specific external links between the external sites of CDs and PSs. Chemical modifications of either narrow or wide edges of natural CDs by various substituents make them possible to produce ionic or nonionic CD derivatives that are capable of forming supramolecular buildings such as vesicles, micelles, or nanoparticles (NPs) that have the capacity to host PSs. With regard to the anticancer PDT application, it has been shown in vitro that this type of vector can improve PS administration and PDT efficacy [151,152]. However, to date, no in vivo PDT study has been devoted to PS–CD nanoassemblies.

- Nanoassemblies using amphiphilic cyclodextrines

Among the various types of CD derivatives, amphiphilic CDs are largely used to form supramolecular nanoassemblies, and have had many applications in the biomedical field [153,154,155,156,157]. Researchers have developed the concept of amphiphilic CDs to adjust the hydrophobic/hydrophilic balance of their construction, leading to the formation of various types of supramolecular nanoassemblies (vesicles, micelles, NPs…) [158,159,160,161]. Amphiphilic CDs can be obtained by enzymatic pathways or by grafting various substituent groups via amino, amido, thio, ester, and ether bonds [155]. 

All the studies described below are drawn from the work of Mazzaglia et al.

**Figure 35:**
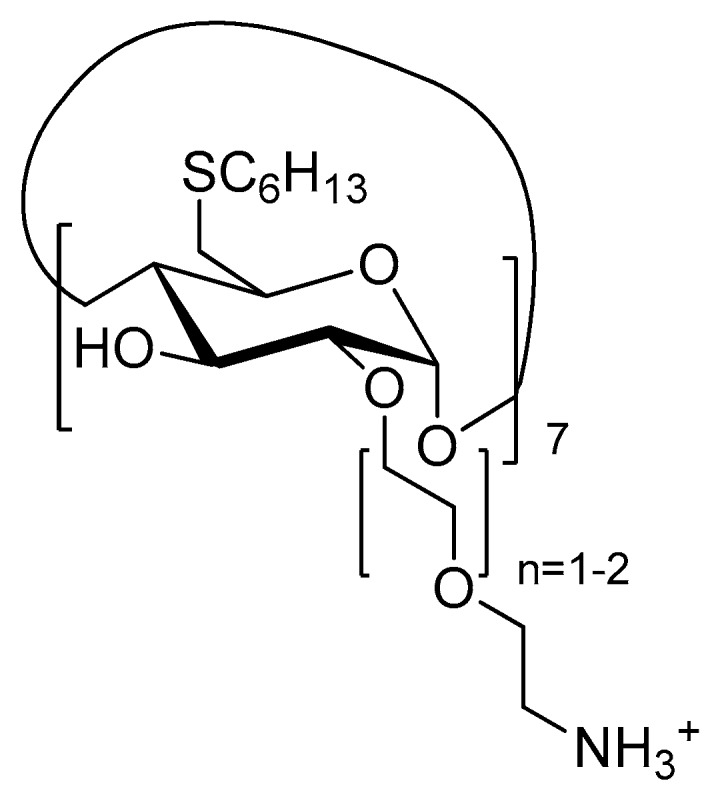
Chemical structure of SC_6_-β-CD-NH_2_ vesicles.

In two articles published in 2003 and 2005 [162,163], Mazzaglia et al. investigated the development of a new carrier–PS system TPPS_4_/SC_6_-β-CD-NH_2_ composed of amphiphilic heptakis(2-*o*-amino-*O*-oligo(ethylene oxide)-6-hexylthio)-β-CD (SC_6_-β-CD-NH_2_, Figure 35) self-assembled into vesicles and an encapsulated water-soluble porphyrin (TPPS_4_). The authors showed that TPPS_4_/SC_6_-β-CD-NH_2_ nanoaggregates produced ^1^O_2_, but in lower amounts than free TPPS_4_. However, it was found that SC_6_-β-CD-NH_2_ vesicles seemed to be an efficient carrier for intracellular PS delivery.

**Figure 36:**
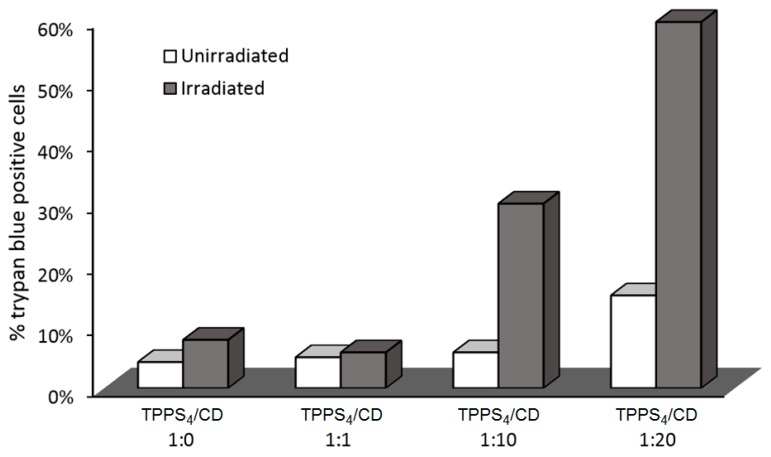
Cell death percentage in HeLa through trypan blue assay. HeLa cells treated with TPPS_4_ alone as a control, and with TPPS_4_/SC_6_-β-CD-NH_2_ nanoassemblies at 1:2, 1:10, and 1:20 molar ratios, respectively before and after exposition to visible light irradiation for 30 min. Adapted from Sortino; Mazzaglia; Scolaro; Merlo; Valveri; Sciortino [164].

In 2006, Mazzaglia et al. [164] investigated the PDT activity of TPPS_4_/SC_6_-β-CD-NH_2_ nanoassemblies. It was found that they had a size ranging from 100 nm to 1000 nm, and that ^1^O_2_ generation was highly dependent on the SC_6_-β-CD-NH_2_ concentration in nanoassemblies. Based on in vitro studies, the TPPS_4_ internalization and PDT efficiency of nanoassemblies using HeLa cells were found to be highly dependent on the TPPS_4_/SC_6_-β-CD-NH_2_ molar ratio. Molar ratio of 1:10 seemed to be the best ratio in terms of TPPS_4_ internalization, which is the highest percentage of cells alive before irradiation and a considerably high percentage of cell death after irradiation (Figure 36). The authors concluded that TPPS_4_/SC_6_-β-CD-NH_2_ nanoassemblies can be used as potential candidates for PDT application.

**Figure 37:**
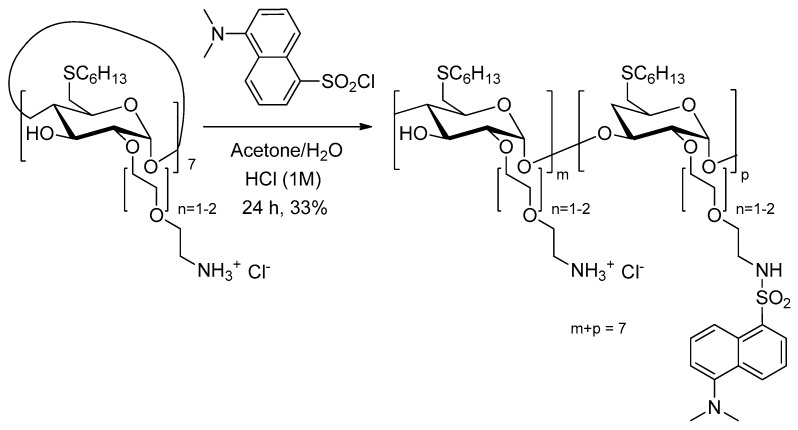
Functionalization of SC_6_-β-CD-NH_2_ with the dansyl fluorophore.

In 2011, the same team [165] used the above amphiphilic TPPS_4_/SC_6_-β-CD-NH_2_ nanoassemblies covalently functionalized by the dansyl fluorophore. The resulting supramolecular system showed a good PS delivery while allowing a simultaneously detection of carrier and PS in tumor cells (Figure 37).

**Figure 38:**
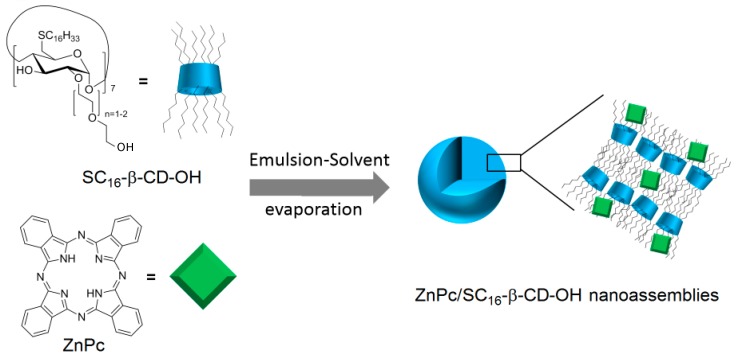
Molecular structure of SC_16_-β-CD-OH, ZnPc, and corresponding ZnPc/SC_16_-β-CD-OH nanoassembly. Adapted from Conte; Scala; Siracusano; Leone; Patane; Ungaro; Miro; Sciortino; Quaglia; Mazzaglia [166].

In 2014, Mazzaglia et al. [166] synthesized the hydroxylated analog of SC_6_-βCD-NH_2_ (noted SC_16_-βCD-OH), and developed a novel biodegradable phototherapeutic nanoassembly ZnPc/SC_16_-β-CD-OH based on the self-assembly in aqueous media of heptakis(2-oligo(ethylene-oxide)-6-hexadecylthio-)-β-CD (SC_16_-βCD-OH) and zinc-phthalocyanine (ZnPc) (Figure 38). ZnPc/SC_16_-β-CD-OH nanoassemblies have a hydrodynamic diameter of around 200 nm, and a shown ability to produce ^1^O_2_.

**Figure 39:**
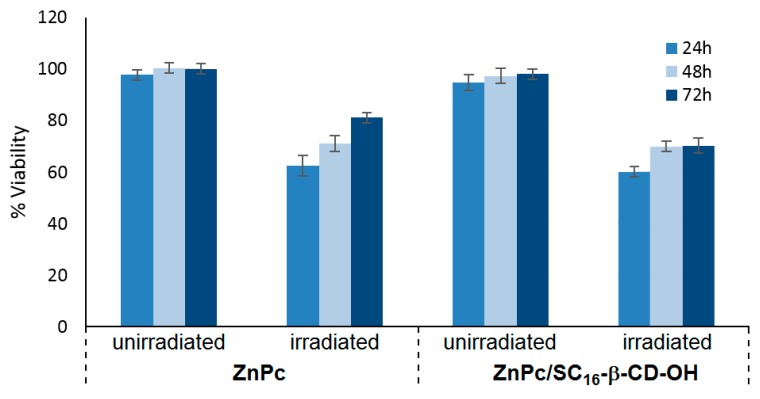
Effects of ZnPc and ZnPc/SC_16_-β-CD-OH nanoassemblies on HeLa cells viability at 24 h, 48 h, and 72 h. ZnPc concentration was set at 0.012 µg/mL in all of the samples. Cell viability was quantified before (dark) and after irradiation (λ = 340 nm, 5 J/cm^2^, 30 min) by using MTS assay. Adapted from Conte; Scala; Siracusano; Leone; Patane; Ungaro; Miro; Sciortino; Quaglia; Mazzaglia [166].

The nanoassemblies’ formation was assessed by Dynamic Light Scattering (DLS), zeta potential, ^1^H NMR, TEM, and Scanning Near-Field Optical Luminescence (SNOL) spectroscopy. ZnPc/SC_16_-β-CD-OH nanoassemblies have a hydrodynamic diameter of around 200 nm and a shown ability to produce ^1^O_2_. Cellular uptake and cytotoxicity was investigated in HeLa cancer cells. The in vitro studies showed that ZnPc/SC_16_-β-CD-OH can be internalized in HeLa cells at 37 °C, and their uptake was mediated by endocytosis, which is strongly temperature-dependent. Furthermore, the PDT efficiency of ZnPc/SC_16_-β-CD-OH was performed using HeLa cells under irradiation (λ = 340 nm, 5 J/cm^2^, 30 min), and ZnPc/SC_16_-β-CD-OH showed a better phototoxic activity against HeLa cells than free ZnPc in DMSO (Figure 39).

**Figure 40:**
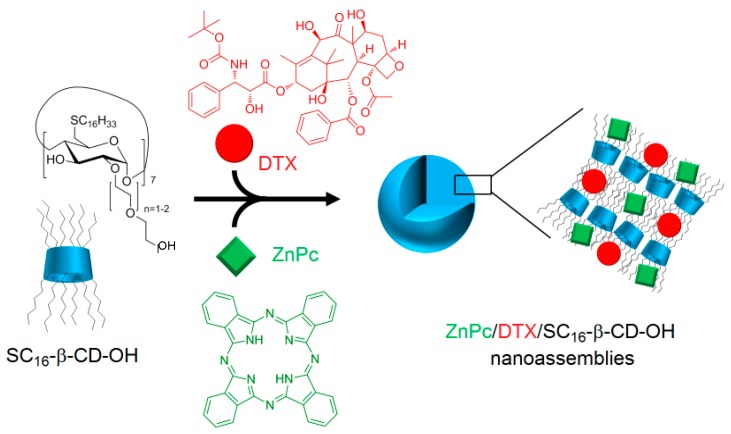
Molecular structure of SC_16_-β-CD-OH, ZnPc, docetaxel (DTX), and corresponding ZnPc/DTX/SC_16_-β-CD-OH nanoassembly. Adapted from Conte; Scala; Siracusano; Sortino; Pennisi; Piperno; Miro; Ungaro; Sciortino; Quaglia; Mazzaglia [167].

Encouraged by the promising results of their previous work, the authors investigated the possibility of using the same nanoasemblies for combined cancer therapies (PDT and chemotherapy) [167]. The new nanoassemblies (hydrodynamic size of 200 nm) were formed as previously described [166] in aqueous medium in the presence of docetaxel (DTX) as the chemotherapeutic drug (ZnPc/DTX/SC_16_-β-CD-OH, Figure 40).

**Figure 41:**
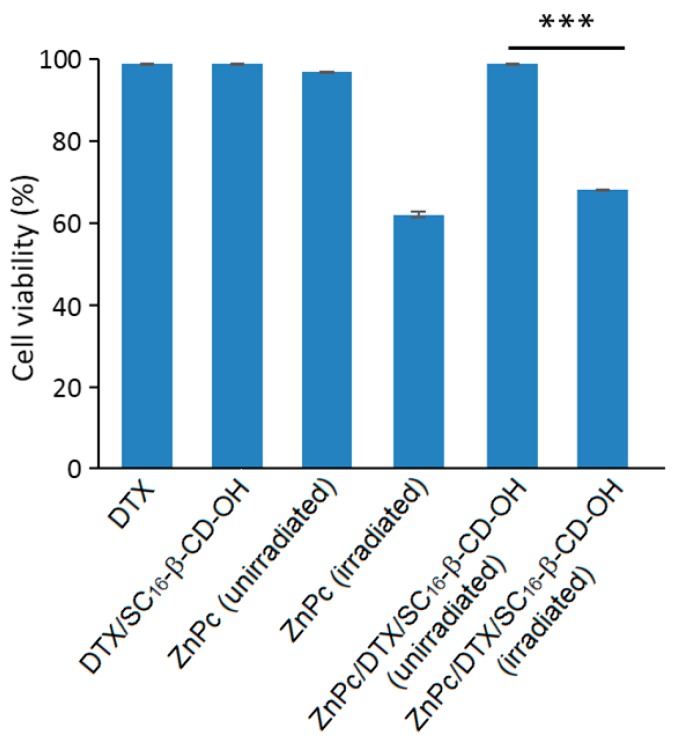
HeLa cell viability (MTS assay, *** *p* < 0.01 by Student’s *t*-test) in the dark and after light irradiation (λ = 340 nm, 5 J/cm^2^, 30 min). The cells were treated with free DTX, free ZnPc, DTX/SC_16_-β-CD-OH nanoparticles (NPs), and ZnPc/DTX/SC_16_-β-CD-OH NPs (10 µg/mL for all samples) and collected at 24 h. Adapted from Conte; Scala; Siracusano; Sortino; Pennisi; Piperno; Miro; Ungaro; Sciortino; Quaglia; Mazzaglia [167].

The new ZnPc/DTX/SC_16_-β-CD-OH nanoassemblies were characterized as previously described [166]. These NPs have a hydrodynamic diameter of around 200 nm, and no specific interaction with the SC_16_-β-CD-OH cavity was shown. Finally, the PDT efficiency of ZnPc/DTX/SC_16_-β-CD-OH NPs using HeLa cells was investigated and compared to free drugs. Based on in vitro studies using HeLa cells, no dark cytotoxicity was detected in all of the treated cells in the dark, but under irradiation (λ = 340 nm, 5 J/cm^2^, 30 min), a similar phototoxic effect was observed for ZnPc/DTX/SC_16_-β-CD-OH NPs, and exhibited a comparable phototoxic effect to that of irradiated free ZnPc in DMSO (Figure 41).

**Figure 42:**
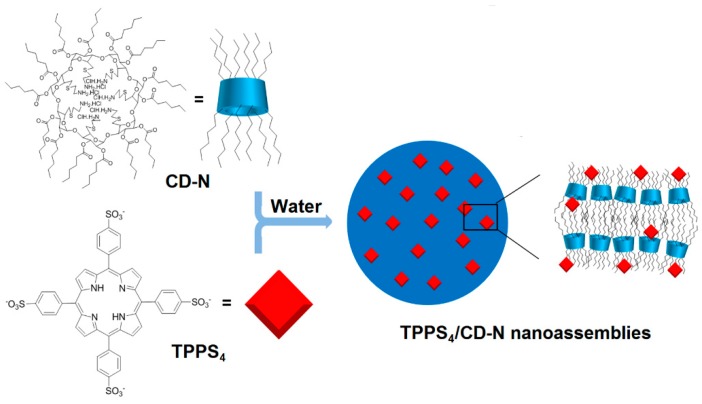
Molecular structure of cyclomaltoheptaose (CD-N), TPPS_4_ and corresponding TPPS_44_/CD-N nanoassembly. Adapted from Mazzaglia; Micali; Villari; Zagami; Pennisi; Mellet; Fernandez; Sciortino; Scolaro [168].

Finally, Mazzaglia et al. in 2017 [168] described the elaboration of a novel nanophototherapeutic using the same emulsion–solvent evaporation procedure in a water environment as described in their previous studies [162,163,165,166,167]. For this novel nanoassembly (hydrodynamic size of around 40 nm) and as reported in their previous work [164], the authors used the same water-soluble anionic PS (TPPS_4_), but this time with a new cationic amphiphilic CD, i.e., heptakis[6-(2-aminoethylthio)-6-deoxy-2,3-di-*O*-hexanoyl] cyclomaltoheptaose (CD-N) (Figure 42).

**Figure 43:**
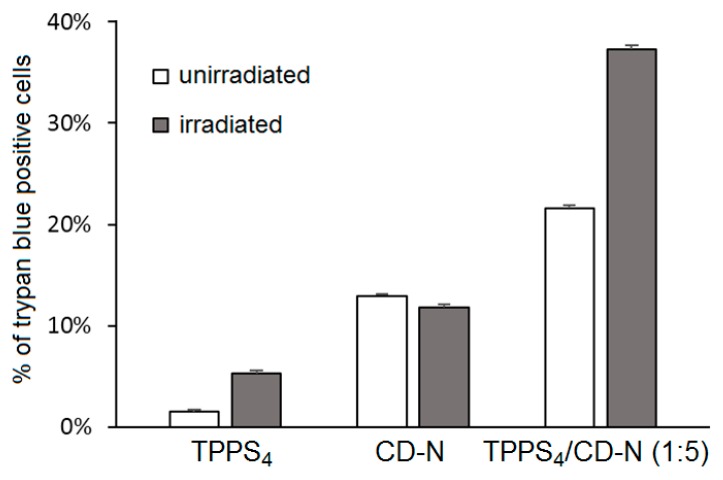
Cell death percentage in HeLa using trypan blue assay. HeLa cells were treated with free TPPS_4_ (1.7 µM) and unloaded CD-N as controls, and with TPPS_4_/CD-N nanoassemblies prepared at 1:5 molar ratios, respectively. TPPS_4_ amount was fixed at 1.7 µM. Samples were exposed to visible light irradiation (λ = 340 nm, 5 J/cm^2^, 30 min). Adapted from Mazzaglia; Micali; Villari; Zagami; Pennisi; Mellet; Fernandez; Sciortino; Scolaro [168].

Cellular uptake was performed on HeLa cells treated with TPPS_4_/CD-N nanoassemblies, and the authors showed that amphiphilic CD-N was able to promote the intracellular delivery of TPPS_4_. The in vitro PDT efficiency of TPPS_4_/CD-N nanoassemblies on the same cell lines model upon visible light irradiation (λ = 340 nm, 5 J/cm^2^, 30 min) were also investigated. In the absence of light, higher dark cytotoxicity was observed in the case of cells treated with the TPPS_4_/CD-N nanosystem compared to those treated with TPPS_4_ alone, which may be due to a higher TPPS_4_ uptake. Interestingly, the TPPS_4_/CD-N nanosystem was found to be more efficient than free TPPS_4_ to induce photodamage upon light irradiation (Figure 43).

The development of supramolecular self-assemblies using amphiphilic CDs as drug delivery nanocarriers may suffer from the conjugate’s lack of stability [169,170,171]. In fact, the chemical bonds that are often used to bind CDs to the drugs can be labile, and thus cause the premature loss of drugs [156,172]. To deal with this problem, Xiong et al. [173] in 2017 used the high affinity of carboranes (CBs) for the β-CD and developed two stable amphiphilic supramolecular nanossemblies based on β-CDs and CBs, and loaded with PS (5-(4-hydroxy-phenyl)-10,15,20-triphenyl-porphyrin, TPP). Host–guest interactions between amphiphilic PEG-modified β-CD (PEG-β-CD) and octyl-carborane (C_8_-CB) led to the formation of 1:1 and 2:1 inclusion complexes named PEG-β-CD/C_8_-CB and 2PEG-β-CD/C_8_-CB. These inclusion complexes self-assembled into spherical NPs and finally loaded with TPP to afford TPP@PEG-β-CD/C_8_-CB and TPP@2PEG-β-CD/C_8_-CB NPs with hydrodynamic sizes of 113 nm and 93 nm, respectively. The in vitro behavior of TPP@PEG-β-CD/C_8_-CB NPs was investigated on human liver cancer cells (HepG2) using confocal microscopy and flow cytometry. The authors showed that NPs were mainly localized in the cytoplasm region, in close vicinity to the nuclei, and provided higher fluorescent signals compared to cells treated with free TPP or with the physical mixture of PEG-β-CD/C_8_-CB and TPP. In vitro cell viability on normal human bronchial epithelial (BEAS-2B) cells, and HepG2 and HeLa cancer cells was investigated by MTT assay under irradiation (λ = 620 nm, 16 mW/cm^2^). No dark cytotoxicity was observed, even at an NP concentration of 8 µg/mL. For TPP@PEG-β-CD/C_8_-CB NPs, it was found that the viability of cancer cells (HepG2 and HeLa) after irradiation was 10 times lower compared to TPP alone or to the physical mixture of PEG-β-CD/C_8_-CB and TPP. Finally, the stability in solution and the photostability of TPP@PEG-β-CD/C_8_-CB NPs were evaluated, and the authors estimated that less than 5% of TPP leaked after five days in physiological solution (10% fetal bovine serum), and only a 5.2% absorbance decrease was observed after 90 min of irradiation.

As complementary information and concerning the elaboration of the nanoassemblies of amphiphilic CDs and porphyrinoid PSs, we can mention also an article published in 2013 by Voskuhl et al. [174]. The authors investigated the self-assembly of a supramolecular ^1^O_2_ photosensitizing system based on host–guest interactions between an adamantane-functionalized ZnPc (Ada-ZnPc) and β-CD vesicles (β-CDVs). The resulted Ada-ZnPc/ β-CDV nanoassemblies allowed an increase of the ^1^O_2_ photosensitizing ability of ZnPc, while preventing its self-aggregation. These results reflect the possible use of this supramolecular assembly as a biocompatible photoactive platform for the design of phototherapeutic agents. Concerning non-porphyrinoid PSs, only one article has been found in the literature. In 2016, Kauscher et al. [175] described the immobilization of photoreactive squaraines (non-porphyrinoid PS) on a supramolecular amphiphilic CD nanoassemblies, and highlighted the ^1^O_2_ production under irradiation and an improvement in the photochemical activity of squaraines. However, no in vitro biological study has yet been made of the impact of these nanoassemblies on the PDT efficiency.

- Nanoassemblies using polymeric cyclodextrines

Besides the supramolecular nanoassemblies based on amphiphilic CD, the researchers are studying new CD derivatives, and some works deal with the use of polymeric CDs for new stable and efficient buildings that can be used for PDT [176].

**Figure 44:**
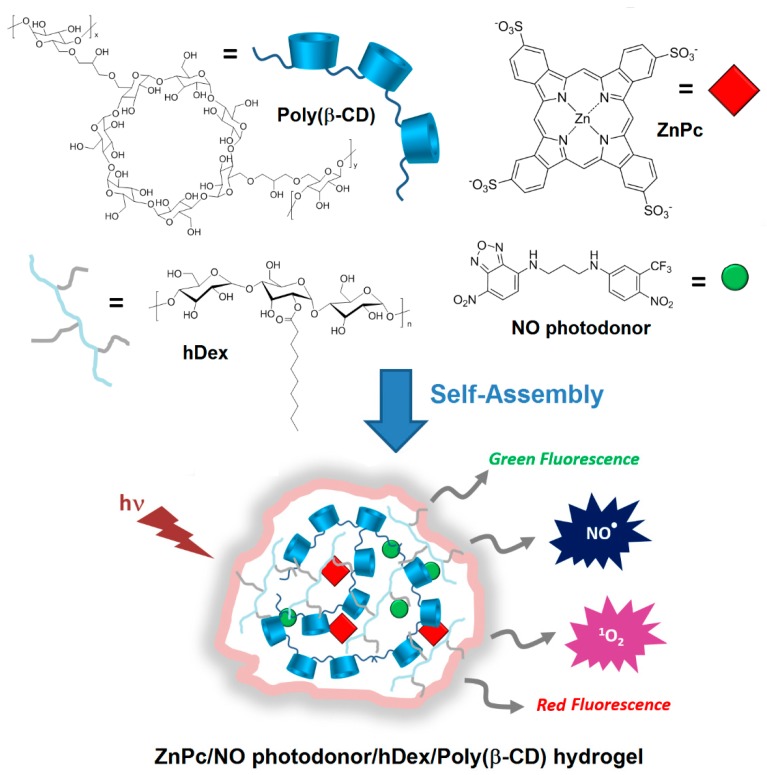
Molecular structure of Poly(β-CD), ZnPc, hydrophobically modified dextran (hDex), NO photodonor, and corresponding ZnPc/NO photodonor/hDex/Poly(β-CD) hydrogel. Adapted from Fraix; Gref; Sortino [177].

In 2014, Sortino et al. [177] engineered supramolecular nanoassemblies that were composed of four different components, i.e., a poly(β-CD) polymer, a hydrophobically modified dextran (hDex), an anionic zinc phthalocyanine (ZnPc), and a NO photodonor. The assembly occurred spontaneously in aqueous medium forming a “Lock and Key” hydrogel, since the alkyl chains of hDex were included in the cavity of poly(β-CD) (Figure 44). The authors described for the first time a hydrogel system that was suitable for (1) producing both red and green fluorescence signals, (2) photoreleasing simultaneously two cytotoxic species (^1^O_2_ and NO^•^), and (3) inducing an amplified cancer cell death (Figure 44).

**Figure 45:**
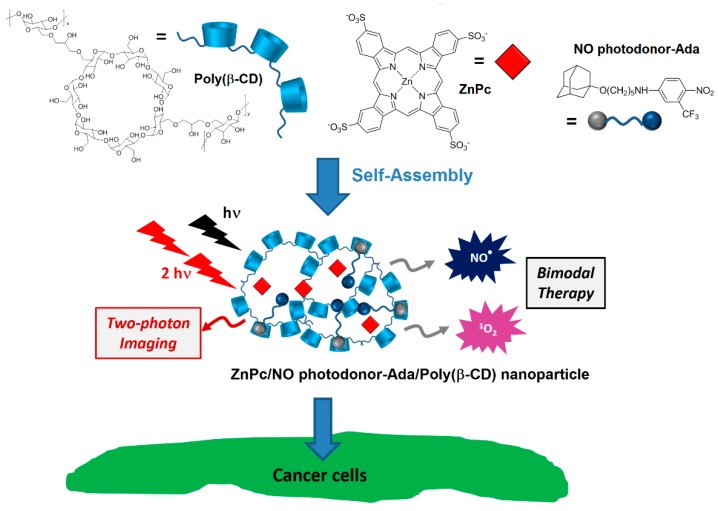
Molecular structure of Poly(β-CD), ZnPc, an NO photodonor attached to an adamantane moiety (NO photodonor-Ada), and corresponding ZnPc/NO photodonor-Ada/Poly(β-CD) nanoparticle. Adapted from Kandoth; Kirejev; Monti; Gref; Ericson; Sortino [178].

In that same year, Sortino et al. [178] engineered supramolecular nanoassemblies composed of three different components, i.e., a poly(β-CD) polymer, an anionic zinc phthalocyanine (ZnPc), and an NO photodonor attached to an adamantane moiety (NO photodonor-Ada). The adamantane unit is known to form an inclusion complex with the cavity of β-CD. The self-assembly led to the formation of ZnPc/NO photodonor-Ada/Poly(β-CD) NPs with an average hydrodynamic diameter of 35 nm (Figure 45).

**Figure 46:**
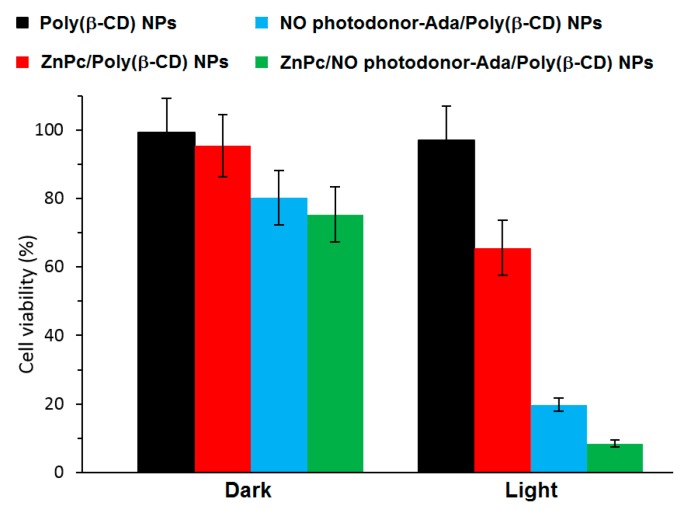
Dark and photoinduced mortality of A431 cells incubated with aqueous dispersion of Poly(β-CD), ZnPc/Poly(β-CD), NO photodonor-Ada/Poly(β-CD), and ZnPc/NO photodonor-Ada/Poly(β-CD) NPs. The samples were simultaneously irradiated with 405 nm (10 J/cm^2^) and 633 nm (10 J/cm^2^) LED sources. [Poly(β-CD)] = 11 µM (7.75 mM in β-CD), [ZnPc] = 15 µM, [NO photodonor-Ada] = 40 µM. Adapted from Kandoth; Kirejev; Monti; Gref; Ericson; Sortino [178].

To validate the feasibility of ZnPc/NO photodonor-Ada/Poly(β-CD) NPs as bimodal phototherapeutic agents, an in vitro PDT study were performed using human squamous carcinoma (A431) cells (Figure 46). The authors thereby showed that the irradiation of ZnPc/NO photodonor-Ada/Poly(β-CD) NPs with visible light (405-nm and 633-nm light to target the NO photodonor and ZnPc, respectively) triggered the simultaneous delivery of cytotoxic ^1^O_2_ and NO^•^ species, resulting in an amplified cell photomortality due to the synergistic effect of both cytotoxic agents. It was shown also that ZnPc/NO photodonor-Ada/poly(β-CD) NPs could act as two photon emission (TPE) imaging agents.

**Figure 47:**
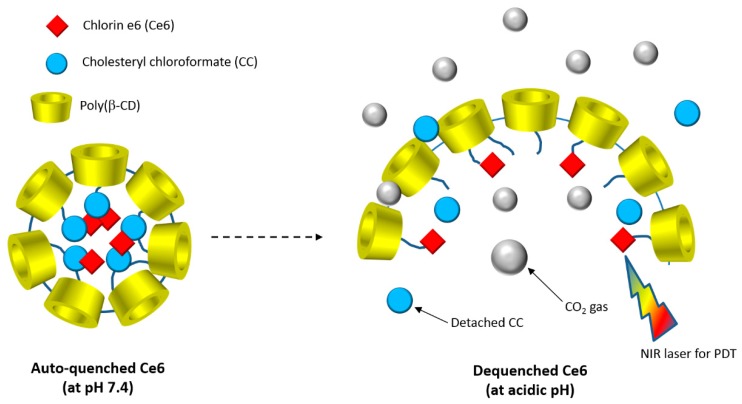
Schematic concept of the poly(β-CD)-*g*-CC-*g*-Ce6 NPs responding to an acidic pH. Adapted from Lee; Oh; Youn; Lee [179]. CC: cholesteryl chloroformate, Ce6: chlorin e6.

In 2015, Lee et al. [179] developed a new biocompatible nanoassembly that was constituted of poly(β-CD) linked by carbonate bonds to cholesteryl chloroformate (CC) and chlorin e6 (Ce6). The self-assembly induced the formation of core-shell poly(β-CD)-*g*-CC-*g*-Ce6 NPs (average particle size of 61 nm) in which the hydrophobic CC and Ce6 core is enclosed with a layer of hydrophilic poly(β-CD) shell. The authors found that the poly(β-CD)-*g*-CC-*g*-Ce6 properties as drug carrier were highly pH-dependent. It was also found that at an acidic pH, poly(β-CD)-*g*-CC-*g*-Ce6 NPs disintegration happened due to the carbonate linkages cleavage producing CO_2_ (Figure 47). 

**Figure 48:**
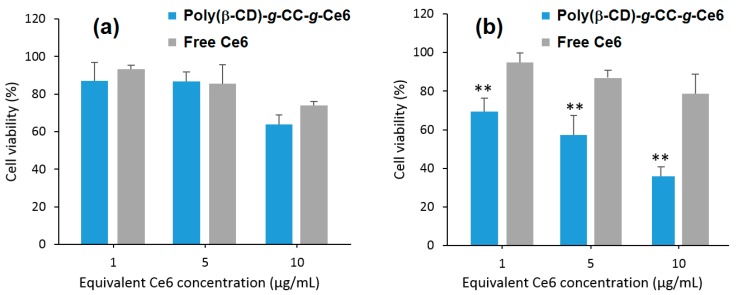
Photoinduced mortality of the human nasopharyngeal epidermal carcinoma (KB) cell line treated with Poly(β-CD)-*g*-CC-*g*-Ce6 NPs (equivalent Ce6 1–10 µg/mL) or free ZnPc (1–10 µg/mL) upon irradiation (λ = 670 nm, 5.2 mW/cm^2^ for 10 min) at (**a**) pH 7.4 and (**b**) pH 6.5. ** *p* < 0.01 compared to free Ce6. Results are expressed as the mean of seven measurements with the vertical bar showing SD. Adapted from Lee; Oh; Youn; Lee [179].

Furthermore, they found that upon irradiation (λ = 670 nm, 5.2 mW/cm^2^ during 10 min), poly(β-CD)-*g*-CC-*g*-Ce6 NPs have the ability to produce more ^1^O_2_ at acidic pH (tumor environment, pH 6.5) compared to the physiological pH (pH 7.4). The in vitro studies performed on the human nasopharyngeal epidermal carcinoma (KB) cell line showed that compared to Ce6 alone, a highest cellular uptake at acidic pH and mainly in nucleus for poly(β-CD)-*g*-CC-*g*-Ce6 NPs was observed. No dark cytotoxicity was detected for the poly(β-CD)-*g*-CC-*g*-Ce6 NPs. However, upon irradiation (λ = 670 nm, 5.2 mW/cm^2^, during 10 min), poly(β-CD)-*g*-CC-*g*-Ce6 NPs exhibited stronger phototoxicity at pH 6.5 compared to that at pH 7.4 (Figure 48). These results highlighted the potential usefulness of poly(β-CD)-*g*-CC-*g*-Ce6 NPs in anticancer PDT treatment.

As complementary information concerning the elaboration of nanoassemblies polymeric CDs and non-porphyrinoid PSs, only one article has been found in the literature. In 2014, Kirakci et al. [180] described three supramolecular nanoassemblies composed by poly(β-CD) and octahedral molybdenum cluster complexes (non-porphyrinoid PSs). The resulting assemblies afforded hydrogel particles (hydrodynamic diameter from 160 nm to 240 nm) without adversely affecting the photophysical properties of the octahedral molybdenum cluster complexes (red luminescence and high quantum yield). The photophysical properties of these hydrogel particles were associated with the oxygen sensitivity of the luminescence, making them interesting for a usefulness as potential dual agents for PDT/boron neutron capture therapy.

- Other cyclodextrin–photosensitizer nanoassemblies

Aside from the supramolecular CD–PS nanoassemblies based on PSs and chemically modified CDs, some teams wished to reverse this strategy by forming nanoassemblies with CDs and chemically modified PSs.

**Figure 49:**
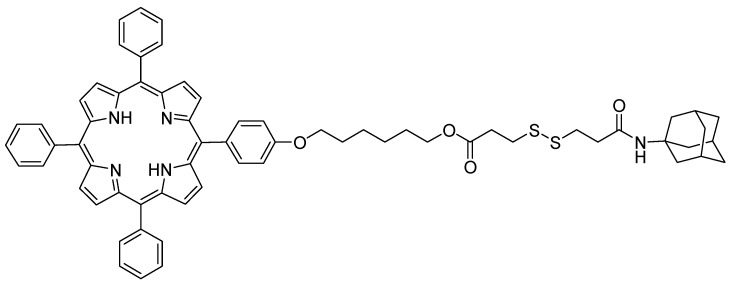
Chemical structure of a porphyrin derivative containing a disulfide bond (S-S) and an adamantane (Ada) group (TPPC_6_-SS-Ada).

In 2015, Liu et al. [181] synthesized a supramolecular system using poly(ethylene glycol 400)-β-CD (PEG_400_-β-CD) and a porphyrin derivative containing a disulfide bond (S-S) and an adamantane (Ada) group (TPPC_6_-SS-Ada, Figure 49). 

**Figure 50:**
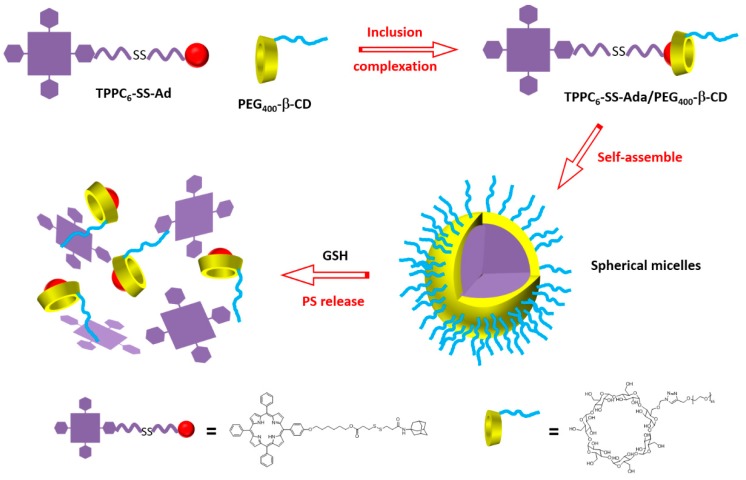
Self-assembly and disaggregation process of TPPC_6_-SS-Ada/PEG_400_-β-CD micelles. Adapted from Liu; Ma; Xu; Liu; Zhang [181].

The host–guest interactions between PEG_400_-β-CD and TPPC_6_-SS-Ada led to the formation of TPPC_6_-SS-Ada/PEG_400_-β-CD polypseudorotaxanes (PPRs) that were able to self-assemble into spherical micelles in aqueous solution (average particle size of around 72 nm, Figure 50). The S–S linkage can be cleaved in reducing intracellular microenvironment. The high level of glutathione (GSH) in cytosol could allow the PS release.

For comparison, the authors also synthesized the TPPC_6_-Ada/PEG-β-CD analog without the disulfide bridge. The authors proved that the S–S bond could be cleaved upon the addition of GSH. Moreover, an in vitro study using MCF-7 cells showed that free porphyrin accumulated less than TPPC_6_-SS-Ada/PEG_400_-β-CD, which might be taken up through an endocytosis process. No dark toxicity could be observed for free porphyrin, TPPC_6_-SS-Ada/PEG-β-CD, and TPPC_6_-Ada/PEG_400_-β-CD micelles, even up to 100 mg/µL of porphyrin. The phototoxic effect upon irradiation with a visible light LED lamp (400 mW/cm^2^) for 20 min was the best for TPPC_6_-SS-Ada/PEG_400_-β-CD with an IC_50_ of 31 µg/mL. At 100 mg/µL of porphyrin, cell viability was 60% for TPPC_6_-Ada/PEG_400_-β-CD, and around 35% for TPPC_6_-SS-Ada/PEG_400_-β-CD. This better result is due to the release of the porphyrin upon cleavage of the S–S bond with GSH. The authors were the first to report the utilization of polypseudorotaxanes (PPRs) for PDT.

Based on the work above described about PPRs for PDT [181], Tong et al. [182] used a similar strategy in 2016 to develop GSH activatable PS-conjugated PPR nanocarriers (Ce6-SS-α-CD/PEG-*b*-PMPC with an average size of 60 nm) for photodynamic theranostics. This time, the supramolecular system was based on host–guest interactions between PEG-*b*-poly (2-methacryl-oyloxyethyl phosphorylcholine) (PEG-*b*-PMPC) block copolymers and a chlorin e6 derivative containing a disulfide bond and α-CD (Ce6-SS-α-CD). The in vitro cellular redox activatable behavior of Ce6-SS-α-CD/PEG-*b*-PMPC NPs was investigated on human oral epidermoid carcinoma (KB) cells using confocal microscopy and flow cytometry. The authors observed (1) a clear red fluorescence of Ce6 in KB cells without any treatment, indicating an efficient internalization of NPs, (2) the strongest intracellular Ce6 fluorescence in the presence of high GSH concentration, which became weakest in the presence of low GSH concentration. The in vitro study of KB cells under irradiation (λ = 660 nm, 50 mW/cm^2^) showed a better phototoxic effect on cells treated with Ce6-SS-α-CD/PEG-*b*-PMPC NPs compared to cells treated with free Ce6. Finally, in vivo fluorescence imaging-guided PDT of Ce6-SS-α-CD/PEG-*b*-PMPC NPs in tumor-bearing mice (tumor size of 250 mm^3^) highlighted an impressive PDT effect on the tumor size after 14 days of therapy. All of these results demonstrated the ability of Ce6-SS-α-CD/PEG-*b*-PMPC NPs to exhibit redox activatable fluorescence signal and ROS generation for photodynamic theranostics.

**Figure 51:**
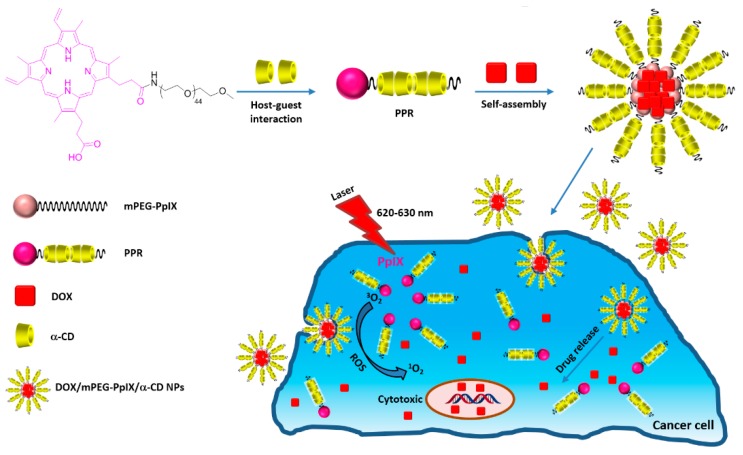
Illustration of polypseudorotaxane doxorubicin (DOX)/mPEG-PpIX/α-CD NPs with dual PDT/chemotherapy effect. Adapted from Xu; Li; Cheng; Zhang; Cao; Gao; He [183].

In 2017, Xu et al. [183] developed polypseudorotaxane NPs (mPEG-PpIX/α-CD) based on self-assembly of mPEG-protoporphyrin IX (mPEG-PpIX) conjugate and α-CDs via host–guest interactions, followed by a chemotherapy drug (doxorubicin, DOX) encapsulation (DOX/mPEG-PpIX/α-CD NPs) with an average size of 89 nm, as shown in Figure 51.

**Figure 52:**
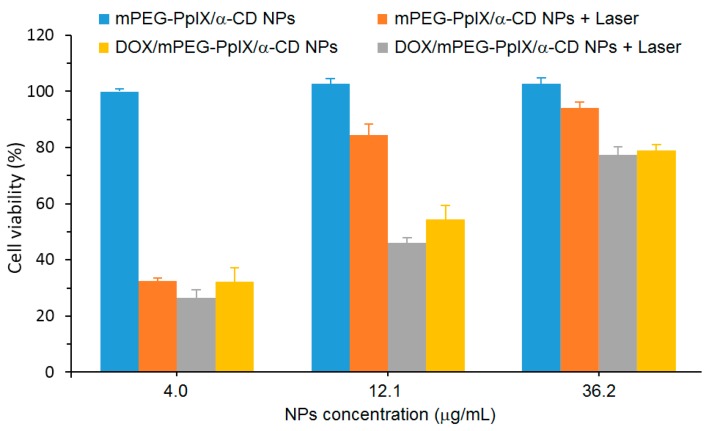
Cell viability of HepG2 cells incubated with mPEG-PpIX/α-CD and DOX/mPEG-PpIX/α-CD NPs before and after laser irradiation (λ = 620–630 nm) at different NPs concentration. Adapted from Xu; Li; Cheng; Zhang; Cao; Gao; He [183].

A cytotoxicity study of mPEG-PpIX/α-CD NPs using L929 fibroblasts cells was performed, and low dark cytotoxicity was found after the 48 h incubation of cells with mPEG-PpIX/α-CD NPs, even with a high concentration of NPs (80% cell viability at a NP concentration of 300 µg/mL). The in vitro PDT efficiency of mPEG-PpIX/α-CD and DOX/mPEG-PpIX/α-CD NPs was evaluated with different NP concentrations (4.0 µg/mL, 12.1 µg/mL, and 36.2 µg/mL) with diiode laser irradiation (λ = 620–630 nm) using HepG2 cells (Figure 52). For mPEG-PpIX/α-CD NPs, no dark cytotoxicity was observed, whatever the concentration used. However, the cell viability upon irradiation was found to be NP concentration-dependent with a higher impact in the presence of DOX. These results revealed that DOX/mPEG-PpIX/α-CD NPs exhibited synergistic PDT/chemotherapy effects under laser irradiation.

As complementary information and concerning the elaboration of supramolecular nanoassemblies based on CDs and chemically modified PSs, we can also mention the work of Jin et al. [184] in 2015. This work described the development of supramolecular hydrogels based on host–guest interactions between amphiphilic PS cored and α-CD leading to the formation of PPRs, which were subsequently self-assembled into hydrogels and loaded with DOX (chemotherapy drug). The resulted supramolecular DOX@PS/α-CD hydrogel exhibited efficient DOX release and ^1^O_2_ generation upon light irradiation, which makes this promising for both cancer chemotherapy drug delivery systems and as a potential PDT agent.

Aside from the supramolecular CD–PS nanoassemblies based on PSs (or chemically modified PSs) and chemically modified CDs (or CDs), another strategy employed by Zhang et al. [171] involved the use of platinium(IV) prodrug bridged β-CD dimer (Pt^IV^(β-CD)_2_) and 5,10,15,20-tetrakis(1-adamantyl-pyridinium-4-yl)porphyrin (TPyP-(Ada)_4_). The authors investigated the formation of Pt^IV^(β-CD)_2_/TPyP-(Ada)_4_ NPs (average size of around 100 nm) by host–guest interactions (stable 2:1 CD-PS inclusion complex) for chemo-photodynamic dual therapy against cisplatin-resistant cancer cells. The in vitro behavior of Pt^IV^(β-CD)_2_/TPyP-(Ada)_4_ NPs was investigated on cisplatin-resistant human lung adenocarcinoma epithelial cells (A549R) using confocal microscopy and flow cytometry. Pt^IV^(β-CD)_2_/TPyP-(Ada)_4_ NPs were internalized by A549R cells in the form of NPs, and a rapid spread of Pt^IV^(β-CD)_2_/TPyP-(Ada)_4_ NPs happened in the whole cytoplasm under light irradiation. An in vitro cytotoxicity study on A549R cells in the dark or with light irradiation (λ = 430 nm, 10 mW/cm^2^ for 2 min) showed that Pt^IV^(β-CD)_2_/TPyP-(Ada)_4_ NPs were much more cytotoxic than cisplatin, even in the dark. The cytotoxicity of Pt^IV^(β-CD)_2_/TPyP-(Ada)_4_ NPs was better under light irradiation, owing to the PDT effect (Table 7). All of these results highlighted a further synergistic effect by the combination of PDT treatment. 

### 2.3. Cyclodextrin–Photosensitizer Systems into Hybrid Nanoparticles

As mentioned in Section 2.2, various CD–PS systems, i.e., inclusion complexes, conjugates, and nanoassemblies, were investigated by researchers to improve some PS properties such as drug delivery, water solubility, stability, and ^1^O_2_ production in aqueous media. The main goal of all of these improvements was to obtain the most adapted PDT agent for anticancer therapy. However, CDs alone cannot solve all of the problems related to PSs, and various studies aimed to combine nanoparticles to CD–PS systems to overcome a part of these limitations. 

#### 2.3.1. Cyclodextrin–Photosensitizer Systems into Up-Conversion Nanoparticles

One of the problems limiting the use of many PSs, and that cannot be addressed by using only CD–PS systems, is the limited light penetration depth in biological tissues. This limitation is due to the light absorption and scattering by biological tissue, resulting in ineffective PDT effect in cases where cancer cells are located deeply in the body [185,186,187]. Irradiations in the near-infrared (NIR) region are known to have a penetration depth that is greater than UV visible light, but PSs that can efficiently absorb NIR light are still rare [188]. As a consequence, major efforts are made to develop CD–PS systems such as up-converting NPs (UCNPs), which are known for their ability to absorb and convert NIR light to visible photons efficiently [189,190,191].

**Figure 53:**
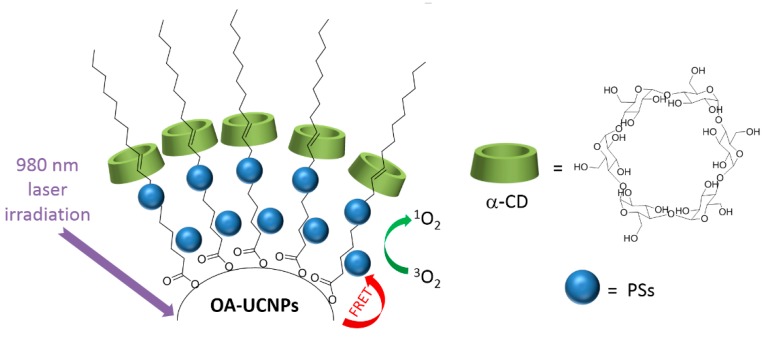
Schematic illustration of 980-nm near-infrared (NIR)-induced PDT using PS@α-CD/oleic acid-capped NaYF_4_:Yb/Er up-converting nanoparticle (OA-UCNP) complexes. Adapted from Tian; Ren; Yan; Jian; Gu; Zhou; Jin; Yin; Li; Zhao [73].

In 2013, Tian et al. [73] synthesized red-emitting oleic acid-capped NaYF_4_:Yb/Er UCNPs (OA-UCNPs) functionalized with α-CD and loaded by different PSs (Figure 53). Firstly, functionalizing the OA-UCNPs with α-CD aimed to tune the hydrophobic character of the OA-UCNPs and make it water-soluble in a host–guest strategy. The hydrophobic cavity of α-CD interacted with the hydrophobic oleic acid surfactants that occupied the surface of the OA-UCNPs.

**Figure 54:**
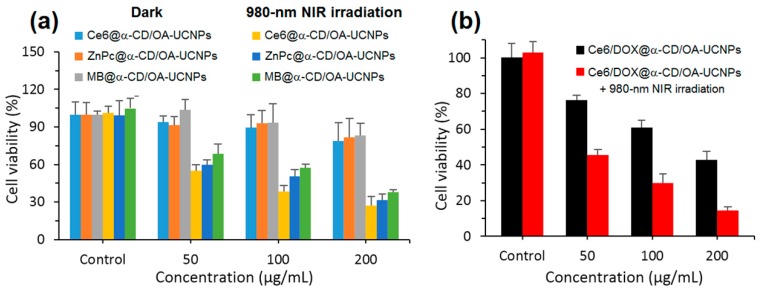
Dark and photoinduced (λ = 980 nm, 1 W/cm^2^ for 5 min) mortality of A549 cells treated with (**a**) α-CD/OA-UCNPs as control, Ce6@α-CD/OA-UCNPs, ZnPc@α-CD/OA-UCNPs, MB@α-CD/OA-UCNPs, and (**b**) Ce6/DOX@α-CD/OA-UCNPs at different concentrations. Adapted from Tian; Ren; Yan; Jian; Gu; Zhou; Jin; Yin; Li; Zhao. [73].

The biocompatibility of the yielded α-CD/OA-UCNPs was assessed by exposing the human epithelial lung cancer (A549) cells to different concentrations of the UCNPs (12.5 µg/mL to 400 µg/mL) in the dark. The α-CD/OA-UCNPs left more than 90% of the cells alive. Moreover, the efficacy of α-CD/OA-UCNPs in the in vivo imaging of deep tissues was further demonstrated through their inoculation into a Kunming mouse (10 mm depth) and observing their red emission under 980-nm NIR excitation. After those validations, and for their efficient use in PDT, OA-capped Mn^2+^-doped NaYF^4^-based UCNPs (OA-UCNPs) were loaded by three PSs, i.e., chlorine e6 (Ce6), zinc phthalocyanine (ZnPc), and methylene blue (MB) via hydrophobic interactions to form a stable donor-acceptor system (PS@α-CD/OA-UCNPs). The NIR irradiation of PS@α-CD/OA-UCNPs could indirectly activate the loaded PSs that in their turn generated large levels of ^1^O_2_ in contrast to the free PS and the bare α-CD/OA-UCNPs. The ^1^O_2_ production was time and dose-dependent for all three PSs, with different levels, which were probably due to their different absorption profiles or loading capacities. The pre-mentioned advantageous properties of the PS@α-CD/OA-UCNPs enabled this system to reduce the viability of A549 cells under NIR irradiation at 980 nm while keeping a very low dark toxicity for all the three PSs (Figure 54a). It is noteworthy to mention that Ce6@α-CD/OA-UCNPs yielded the largest phototoxicity among the three tested PSs. Encouraged by these phototoxicity results, the authors co-loaded a PDT agent (Ce6) and a chemotherapy drug (DOX) into their α-CD/OA-UCNPs to estimate their efficiency in a combined therapy. In response to the change in pH from more basic to a more acidic media, DOX showed a high tendency to detach as it became more hydrophilic. On the contrary, Ce6 was barely released from the α-CD/OA-UCNPs under the same conditions. This behavior of Ce6 conserved the PDT effect that was based on the proximity of both α-CD/OA-UCNPs and Ce6 to guarantee the latter’s activation by Förster Resonance Energy Transfer (FRET). Although the presence of DOX in the Ce6/DOX@α-CD/OA-UCNPs triggered a larger phototoxicity toward A549 cells under 980 nm-NIR excitation, in parallel, it caused a higher dark toxicity (Figure 54b).

**Figure 55:**
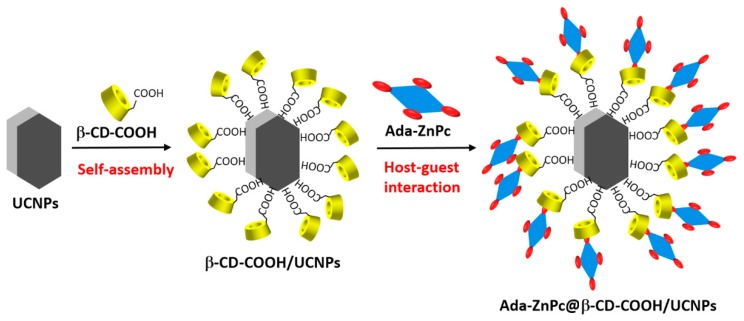
Schematic representation of Ada-ZnPc@β-CD-COOH/UCNP complex. Adapted from Wang; Jin; Chen; Zhou; Zhou; Wei [192].

Conjugating the CD to the UCNPs aims to confine the PSs, prevent their release, and maintain their accessibility to the UCNPs to ensure the activation of the PS through the pre-mentioned FRET process. Thus, to avoid the liberation of ZnPc and strengthen the interactions between ZnPc and CD/UCNPs, Wang et al. [192] functionalized ZnPc with adamantane (Ada-ZnPc) and synthetized a stable Ada-ZnPc@β-CD-COOH/UCNP complex (Figure 55). The prepared Ada-ZnPc@β-CD-COOH/UCNPs had the advantageous properties of β-CD-COOH, especially its good water solubility. In addition, under NIR excitation (980 nm), the β-CD-COOH/UCNPs emitted in red at about 660 nm, which enabled the excitation of Ada-ZnPc through FRET.

**Figure 56:**
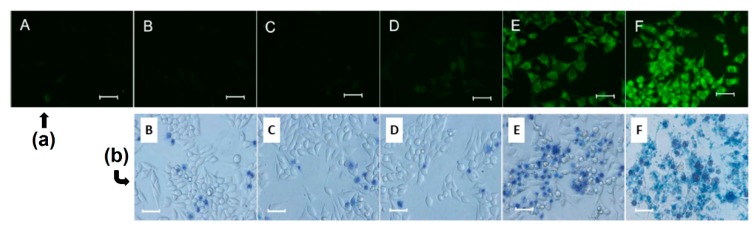
(**a**) Singlet oxygen sensor green (SOSG) fluorescence images of HeLa cells. (**b**) Micrographs of HeLa cells stained with trypan blue of (**A**) control cells, and when exposed to (**B**) β-CD-COOH/UCNPs, (**C**) β-CD-COOH, (**D**) Ada-ZnPc, (**E**) β-CD-COOH/UCNPs + Ada-ZnPc, and (**F**) Ada-ZnPc@β-CD-COOH/UCNPs after 980-nm laser irradiation. Bar = 100 μm. Reproduced by permission of The Royal Society of Chemistry [192].

The ability of Ada-ZnPc@β-CD-COOH/UCNPs to produce ^1^O_2_ in water was indirectly detected using anthracene-9,10-dipropionic acid (ADPA) as a fluorescent probe. Based on the in vitro ^1^O_2_ production results using HeLa cells when exposed to NIR irradiation, a significant level of ^1^O_2_ was generated by Ada-ZnPc@β-CD-COOH/UCNPs compared to β-CD-COOH/UCNPs and Ada-ZnPc. The amount of ^1^O_2_ increased proportionately with the time of light exposure. Separately, the components of the Ada-ZnPc@β-CD-COOH/UCNP complex could not produce any ROS; yet, a lower level of ^1^O_2_ was generated when β-CD-COOH/UCNPs and Ada-ZnPc were physically mixed together. The superior ^1^O_2_ production in the case of Ada-ZnPc@β-CD-COOH/UCNPs was attributed to the short distance between the β-CD-COOH/UCNPs and Ada-ZnPc that was convenient to establish an efficient FRET, which was not the case when those two components were only mixed together. However, when the intracellular generation of ^1^O_2_ was assessed after NIR irradiation using a SOSG (singlet oxygen sensor green) probe and HeLa cells, all of the cells treated with β-CD-COOH/UCNPs, Ada-ZnPc, and Ada-ZnPc@β-CD-COOH/UCNPs exhibited a fluorescence of the probe that was strongest in the case of Ada-ZnPc@β-CD-COOH/UCNPs (Figure 56a). These results assured their capability of elaborating ^1^O_2_ inside the cells. The Ada-ZnPc@β-CD-COOH/UCNP complex was proved to localize into the cytoplasm of the cells, similarly to the unmodified β-CD-COOH/UCNPs. Thus, under NIR excitation, FRET between β-CD-COOH/UCNPs and ZnPc took place; consequently, ZnPc became excited and produced ^1^O_2_, which finally caused the massive destruction of the cells that appeared stained in blue due to their chromatin damage (Figure 56(bF)).

**Figure 57:**
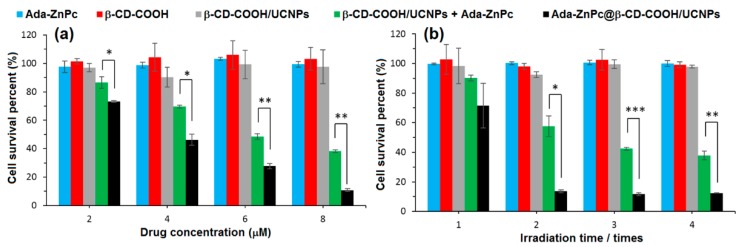
(**a**) Drug dose and (**b**) light dose-dependent in vitro PDT activity for β-CD-COOH/UCNPs, β-CD-COOH, Ada-ZnPc, β-CD-COOH/UCNPs + Ada-ZnPc, and Ada-ZnPc@β-CD-COOH/UCNPs after 980-nm laser irradiation. * *p* < 0.05, ** *p* < 0.01, *** *p* < 0.0001. Adapted from Wang; Jin; Chen; Zhou; Zhou; Wei [192].

The PDT efficiency of the different components was evaluated by trypan blue, excluding experiments and MTT assay. It is noteworthy to mention that this PDT effect also took place with the cells exposed to β-CD-COOH/UCNPs and Ada-ZnPc together, but to a much lower extent than with the complex. The PDT effect appeared to be drug and light dose-dependent (Figure 57). This research effort proved that the intervention of CD made these β-CD-COOH/UCNPs efficient as drug-delivery systems for NIR-triggered PDT.

**Figure 58:**
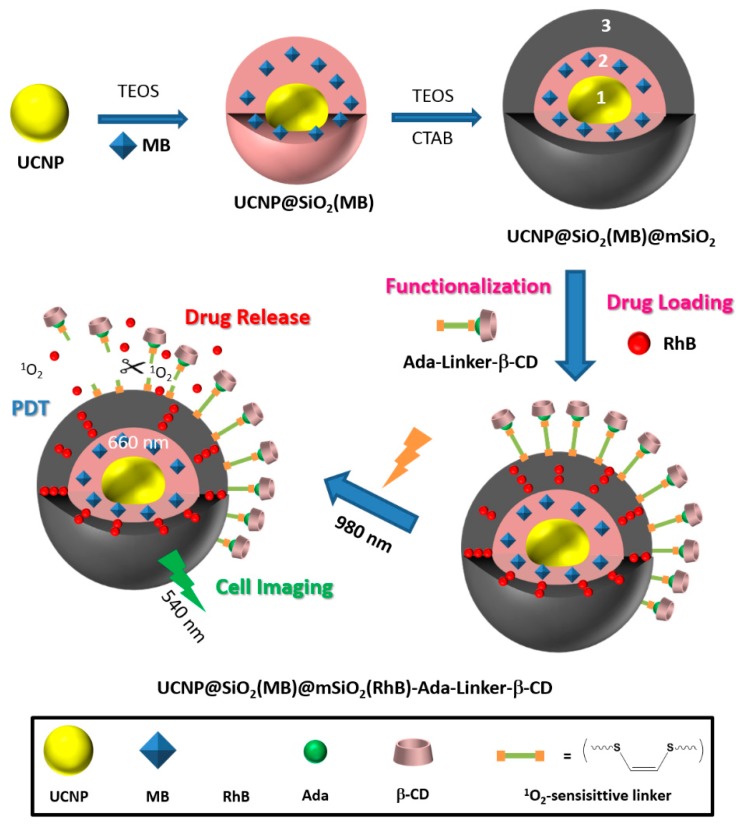
Schematic diagram showing the synthetic procedure of UCNP@SiO_2_(MB)@mSiO_2_(RhB)-Ada-linker-β-CD NPs. Adapted from Wang; Han; Yang; Shi; Liu; Hu; Wang; Liu; Gan [193]. @mSiO_2_(RhB): Ada-linker: adamantane-^1^O_2_-sensitive linker, RhB: rhodamine B-anchored mesoporous silica shell.

In 2016, Wang et al. [193] developed new core-shell NPs constituted of a NaYF_4_:Yb/Er UCNP core (UCNP), a methylene Blue (MB)-loaded silica shell (@SiO_2_(MB), MB as PS), and a rhodamine B-anchored mesoporous silica shell (@mSiO_2_(RhB), rhodamine B (RhB) as model drug). The resulted UCNP@SiO_2_(MB)@mSiO_2_(RhB) NPs were functionalized on the surface with an adamantane-^1^O_2_-sensitive linker (Ada-linker) which forms an inclusion complex with β-CD (Figure 58). The inclusion complexes served to enhance water dispersion of resulting NPs and as « gatekeepers » to prevent PS release. The UCNP@SiO_2_(MB)@mSiO_2_(RhB)-Ada-linker-β-CD NPs (hydrodynamic diameter of 75 nm) could be used for simultaneous photo-responsive drug release, PDT and cell imaging.

Photoluminescence and UV-vis analysis revealed that UCNP@SiO_2_@mSiO_2_(RhB)-Ada-linker-β-CD NPs have a strong emission in the red upon 980-nm NIR excitation, and MB absorbs intensely in the range between 650–670 nm. The sound emission–absorption match at around 660 nm may prompt a FRET. This FRET was proved to exist by observing a decrease of emission intensity at 660 nm when UCNP@SiO_2_@mSiO_2_(RhB)-Ada-linker-β-CD NPs were loaded with MB under 980-nm NIR excitation. Interestingly, the emission spectra of UCNP@SiO_2_(MB)@mSiO_2_(RhB)-Ada-linker-β-CD NPs showed also a strong emission peak at 540 nm (green light), offering the feasibility of in vitro cell imaging. In vitro cytotoxicity on human lung adenacarcinoma (A549) cells were evaluated by MTT assay after the incubation of UCNP@SiO_2_(MB)@mSiO_2_(RhB)-Ada-linker-β-CD NPs. A study of the influence of both UCNP@SiO_2_(MB)@mSiO_2_(RhB)-Ada-linker-β-CD NPs concentration and laser treatment (exposure time at the power density of 2.0 W/cm^2^) allowed authors to determine safe conditions, i.e., [UCNP@SiO_2_(MB)@mSiO_2_(RhB)-Ada-linker-β-CD NPs] = 32 µg/mL with 980-nm NIR irradiation at 2.0 W/cm^2^ during 50 s, for performing cell imaging and PDT studies. The use of UCNP@SiO_2_(MB)@mSiO_2_(RhB)-Ada-linker-β-CD NPs as cell imaging agents was checked via confocal Upconversion Luminescence (UCL)/fluorescence in vitro imaging of A549 cells incubated with UCNP@SiO_2_(MB)@mSiO_2_(RhB)-Ada-linker-β-CD NPs upon 980-nm NIR excitation and in bright field mode. The in vitro imaging study of human lung adenacarcinoma (A549) cells treated with UCNP@SiO_2_(MB)@mSiO_2_(RhB)-Ada-linker-β-CD NPs (32 µg/mL) upon 980-nm NIR excitation (2.0 W/cm^2^ during 50 s) results showed an intense green luminescence and a successful cell uptake of UCNP@SiO_2_(MB)@mSiO_2_(RhB)-Ada-linker-β-CD NPs mainly located at the cytoplasmic regions. The in vitro PDT efficiency of UCNP@SiO_2_(MB)@mSiO_2_(RhB)-Ada-linker-β-CD NPs was evaluated on A549 cells under the previously determined as safe mentioned conditions, and showed a cell viability reaching 50%. Finally, the authors highlighted the photo-release of RhB from UCNP@SiO_2_(MB)@mSiO_2_(RhB)-Ada-linker-β-CD NPs induced by the ^1^O_2_-labile linkers thanks to a periodic 980-nm NIR ON/OFF illumination (2.0 W/cm^2^, periodicity of 10 min).

One year later, the same team [194] described a novel PDT nanoplatform (RB-NH_2_-UCNP@mSiO_2_(Ada)-β-CD) constituted of a NaYF_4_:Yb/Er/Nd@NaYF_4_:Nd UCNP core (UCNP, 40 nm), a Ada-anchored silica shell (@mSiO_2_(Ada), 12 nm), positive amino groups into inner channels of mSiO_2_, a negatively charged PS (Rose Bengal, RB), and β-CD as a solubilizing agent and « gatekeeper » to prevent PS release via an inclusion complex with Ada. Fluorescence microscopy imaging of HeLa cells incubated with RB-NH_2_-UCNP@mSiO_2_(Ada)-β-CD NPs showed that NPs were uptaken by HeLa cells through either endocytosis or micropinocytosis with a localization mainly in cytoplasm. No dark cytotoxicity of RB-NH_2_-UCNP@mSiO_2_(Ada)-β-CD NPs was observed, even with high NP concentrations (250 µg/mL). The in vitro PDT activity of RB-NH_2_-UCNP@mSiO_2_(Ada)-β-CD NPs using HeLa cells under 808-nm NIR excitation increased with the increasing irradiation time exposure (0 min to 10 min), laser power density (0 to 4 W/cm^2^), and NPs concentration (0 to 250 µg/mL). The in vitro results highlighted that under 808-nm NIR irradiation, the green 540 nm up-conversion from UNCP could activate RB to efficiently generate ^1^O_2_ and promote cancer cell death, enabling a high efficient anticancer PDT treatment upon the low heat effect 808-nm excitation. 

#### 2.3.2. Cyclodextrin–Photosensitizer Systems into Other Types of Nanoparticles

As well as the UCNPs being used for improving CD–PS system properties, some studies are devoted to the use of other types of nanoparticles, and are briefly discussed below.

In 2011, Dong et al. [195] developed supramolecular polymer micelles (SMPMs) as intelligent drug delivery systems. SMPMs were constructed from ethylcellulose-graft-poly(ε-caprolactone (EC-g-PCL) and maleic anhydride modified α-CD (Mah-α-CD) derivative via host–guest and hydrophobic interactions. The resulted Mah-α-CD/EC-g-PCL SMPMs were loaded with a PS (5,10,15,20-tetrakis(4-hydroxyphenyl)-21*H*,23*H*-porphyrin, THPP). The in vitro behavior of THPP-loaded SMPMs was investigated on breast tumor (MCF-7) cells using confocal microscopy, and it was found that THPP can be internalized in the MCF-7 cells via SMPMs. In vitro trigger-controlled THPP release was evaluated, and THPP was efficiently released from SMPMs by the addition of l-phenylalanine. The in vitro cell viability on MCF-7 cells was investigated by MTT assay under irradiation (λ = 400 nm, Xe lamp with total light dose of 120 J/cm^2^). No dark cytotoxicity was observed and the photomortality of MCF-7 cells was clearly demonstrated, which increases as the trigger l-phenylalanine concentration increases (from 0 mM to 3 mM). All of these results showed a promising application of the THPP-loaded SMPMs in cancer treatment as a drug delivery and controlled release system.

In 2013, Ma et al. [196] described the covalent conjugation of Ada onto both the surface and nanochannels of mesoporous silica NPs (MSNPs). Hydrophobic ZnPc was then loaded into the nanochannels of the resulted Ada-MSNPs (ZnPc/Ada-MSNPs) and capped with β-CD, which forms an inclusion complex with Ada. The inclusion complexes served to enhance water solubility of β-CD/ZnPc/Ada-MSNPs and act as « gatekeepers » to prevent ZnPc release. In vitro PDT activity on HeLa cells used MTT assay with light irradiation (λ = 675 nm, 2.5 mW/cm^2^). No dark cytotoxicity was observed, and the authors showed a higher phototoxicity after 60 min of light irradiation compared with 30 min. Furthermore, in the case of 30 min of light irradiation, an IC_50_ value of 10 µg/mL was determined.

**Figure 59:**
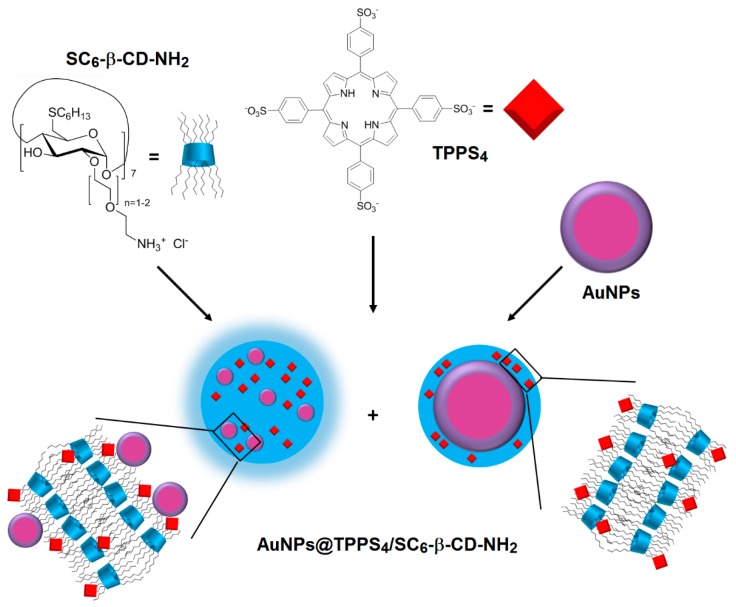
Sketch (not to scale) of the two typologies of ternary AuNPs@TPPS_4_/SC_6_-β-CD-NH_2_ systems presumably formed in aqueous solution. Adapted from Trapani; Romeo; Parisi; Sciortino; Patane; Villari; Mazzaglia [197].

In 2013 also, Mazzaglia et al. [197] built on their results achieved in 2006 [164] concerning the PDT effect of TPPS_4_/SC_6_-β-CD-NH_2_ nanoassemblies (results previously described in Section 2.2.3.). In this new work, the authors investigated the coating of gold nanoparticles (AuNPs) with their previous TPPS_4_/SC_6_-β-CD-NH_2_ nanoassemblies to afford new hybrid assemblies AuNPs@TPPS_4_/SC_6_-β-CD-NH_2_ (average size of around 200 nm, Figure 59). AuNPs were added to the previous TPPS_4_/SC_6_-β-CD-NH_2_ nanoassemblies for the purpose of generating an in vitro dual photothermal (PTT)–PDT effect on HeLa cells.

**Figure 60:**
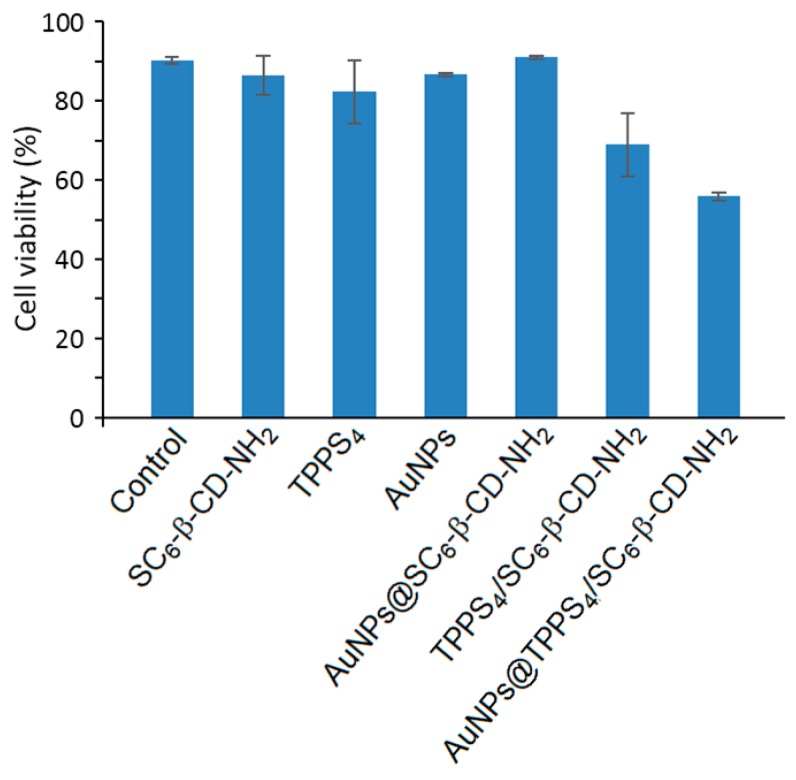
HeLa cell viability (MTS assay). Cells treated with SC_6_-β-CD-NH_2_, TPPS_4_, gold nanoparticles (AuNPs), AuNPs@SC_6_-β-CD-NH_2_, TPPS_4_/SC_6_-β-CD-NH_2_, and AuNPs@TPPS4/SC6-β-CD-NH_2_ compared to negative control (cell untreated). Cells washed in PBS (10 mM, pH 7.4) and analyzed in the dark and after photothermal (PTT)–PDT bimodal treatment. Adapted from Trapani; Romeo; Parisi; Sciortino; Patane; Villari; Mazzaglia [197].

The in vitro behavior of supramolecular AuNPs@TPPS_4_/SC_6_-β-CD-NH_2_ hybrid assemblies was investigated by fluorescence microscopy analysis of Hela cells, and AuNPs@TPPS_4_/SC_6_-β-CD-NH_2_ assemblies were mainly localized in the cytoplasm region. To evaluate the feasibility of using a ternary AuNPs@TPPS_4_/SC_6_-β-CD-NH_2_ system for dual PTT–PDT action, HeLa cells were treated with AuNPs@TPPS_4_/SC_6_-β-CD-NH_2_ NPs and either kept in the dark or irradiated with 532 nm neodymium-doped yttrium aluminium garnet (Nd-YAG) pulsed laser and then by visible light for 30 min. AuNPs@TPPS_4_/SC_6_-β-CD-NH_2_ showed scarce dark toxicity while single components, binary TPPS_4_/SC_6_-β-CD-NH_2_ and AuNPs@SC_6_-β-CD-NH_2_ systems, showed some dark toxicity. After PTT treatment, the toxicity was almost unaltered, except for the ternary system (~30% cell death), demonstrating that AuNPs were playing a role in the cell photodamage. Finally, after PTT–PDT treatment, the cells incubated with the ternary AuNPs@TPPS_4_/SC_6_-β-CD-NH_2_ system showed a further increase of toxicity (~45% cell death), which was higher with respect to the PTT–PDT effect on the cells incubated with binary systems (Figure 60).

As complementary information and concerning the elaboration of supramolecular nanoassemblies based on CD–PS systems into other types of nanoparticles, we can also mention the work of Xu et al. [198] in 2016, who described host–guest interactions between di-β-CD-modified PpIX (PpIX-CD_2_) and azobenzene (AZO)-focused hydrophobic/hydrophilic hyperbranched polymers, leading to the formation of PpIX-bridged Janus particles via self-assembly (PpIX-CD_2_@AZO Janus particles). The obtained supramolecular Janus particles (average size of around 10 nm) were used to build large vesicles (up to 694 nm) by aggregation. UV irradiation (λ = 365 nm) caused vesicles destruction because of the trans-cis isomerization of the AZO unit. A decrease of 25% in the absorbance of the main Soret band indicated some PpIX degradation, but less than free PpIX, in favor of a protection (photostability) effect induced by vesicles. The conversion rate of triiodide ion was measured in buffer solution at 86% after 1 h of irradiation, indicating a high ^1^O_2_ quantum yield production.

### 2.4. Cyclodextrins with Fullerenes

Many researchers, in their quest for the development of a better suitable PS for anticancer PDT treatment, pursue their efforts and grapple with this challenge by trying to develop new original PSs. Among these, one can cite the use of fullerenes. Fullerenes are carbon molecules whose shape can be close to a sphere, an ellipsoid, a tube, or a ring. They are composed of hexagonal rings (such as graphite) but also pentagonal rings, which give the possibility of closed structures. Fullerenes are natural molecules (found in soot) that were identified in 1985 by Kroto et al. [199] who received the Nobel Chemistry Prize in 1996 for this discovery. Fullerenes are extracted from the soot using a multistep procedure. After dissolution of the soot in appropriate organic solvents (solution containing up to 70% of C_60_ ([60]Fullerene) and 15% of C_70_ ([70]Fullerene), as well as other fullerenes), each fullerene fraction is separated using chromatography.

**Figure 61:**
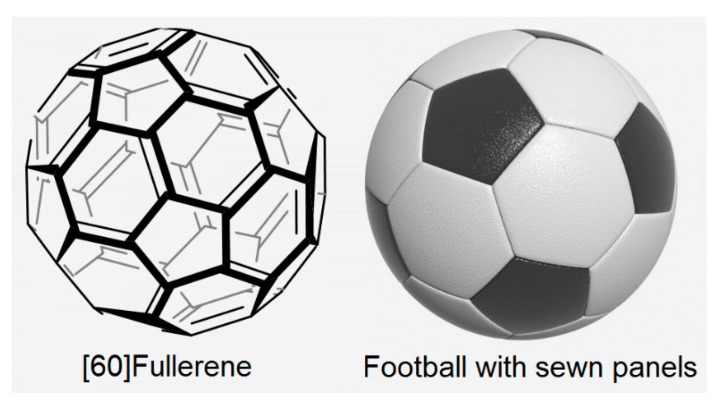
Three-dimensional (3D) representation of [60]Fullerene and a football with sewn panels.

[60]Fullerene is the most stable form of fullerene whose composition includes 60 carbon atoms forming 20 hexagons and 12 pentagons, with a carbon atom at the top of each polygon and a bond at each side of the polygon. Each hexagon is adjacent to three hexagons and three pentagons, and each pentagon is surrounded by five hexagons (Figure 61). [60]Fullerene has the same shape as a traditional football with sewn panels.

Over three decades have passed since the discovery of fullerenes, and the interest for medical applications generated by them over the years has increased considerably [200,201,202,203,204]. Fullerenes combine many very interesting properties, making them good candidates for developing suitable PSs for PDT application [205,206,207,208]. These interesting properties include: (1) a higher photostability and a lower photobleaching compared to tetrapyrroles (porphyrinoid PSs) and synthetic dyes (non-porphyrinoid PSs); (2) both type I (free radicals) and type II (^1^O_2_) ROS generation compared to largely type II for tetrapyrroles; (3) an increase of both overall quantum yield and ROS production; (4) an extension of their absorption spectrum further into the red wavelengths; and (5) an ability to self-assemble into vesicles and functionalize with the aim of improving drug delivery. In spite of all these benefits, pristine [60]Fullerene is poorly soluble in water and biological media and self-assembles into nano-aggregates, limiting its photoactivity [209]. Therefore, it appears obvious that the combination of fullerenes and CDs would be a reasonable alternative for developing ideal PSs for use in anticancer PDT treatment.

**Figure 62:**
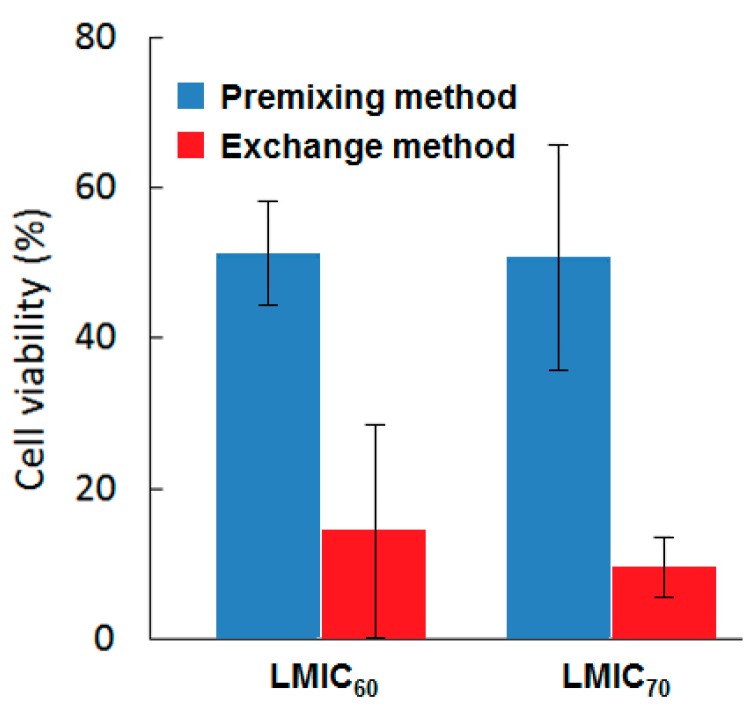
Cell viability following treatment with lipid membrane-incorporated fullerenes LMIC_60_ and LMIC_70_, prepared by the exchange and premixing methods, under excitation at 350–500 nm for 2 h in the case of LMIC_60_ and 30 min for LMIC_70_) ([C_60_] = 2 mM and [C_70_] = 0.3 mM). Adapted from Ikeda [212].

Between 2008–2017, Ikeda et al. focused on the application of cyclodextrin /fullerenes inclusion complexes in PDT [210,211,212,213,214,215,216]. In 2008, Ikeda et al. synthesized lipid membrane-incorporated fullerenes (LMIC_x_: x = 60 or 70) with high concentrations of fullerenes (C_x_) through a simple and time-saving approach [211]. The method was based on an exchange reaction where C_x_ was transferred from the C_x_-γ-CD complex to the liposomes. The elaborated LMIC_60_ and LMIC_70_ possessed an average diameter of about 100 nm. The authors also proved the efficiency of their guest exchange method for the preparation of the lipid membrane-incorporated fullerenes (LMIC_x_) in comparison to the conventional injection and premixing approaches [212]. LMIC_60_ and LMIC_70_ prepared by the guest exchange approach displayed a higher PDT activity accompanied with a better stability and solubility as compared to those prepared by the premixing one (Figure 62). γ-CD were employed in this method to embrace the fullerene in the first step and facilitate the transfer of this fullerene from the cavity of the CD to the liposomes or lipid membrane, where only fullerene was the PS fulfilling the PDT effect.

To investigate the localization and the mode of internalization of the LMIC_x_, only the liposomes were labeled by fluorochrome, and were incubated with HeLa cells. Fullerene was avoided, since it can quench fluorochrome. It appeared that only cationic liposomes were uptaken by the cells via endocytosis to be finally localized into the lysosomes. After 24 h of incubation with HeLa cells, both LMIC_60_ and LMIC_70_ revealed no dark toxicity. When excited at a wavelength between 350–500 nm, LMIC_60_ induced an 85% decrease of cell viability. However, when PDT was performed at a wavelength longer than 400 nm (400–740 nm), only LMIC_70_ was able to induce a significant decrease in the cell viability by 89% versus only 19% of reduction in the case of LMIC_60_. This difference in the performance was due to the greater production of ^1^O_2_ by LMIC_60_. Both LMIC_60_ and LMIC_70_ induced an early apoptotic cell death.

**Figure 63:**
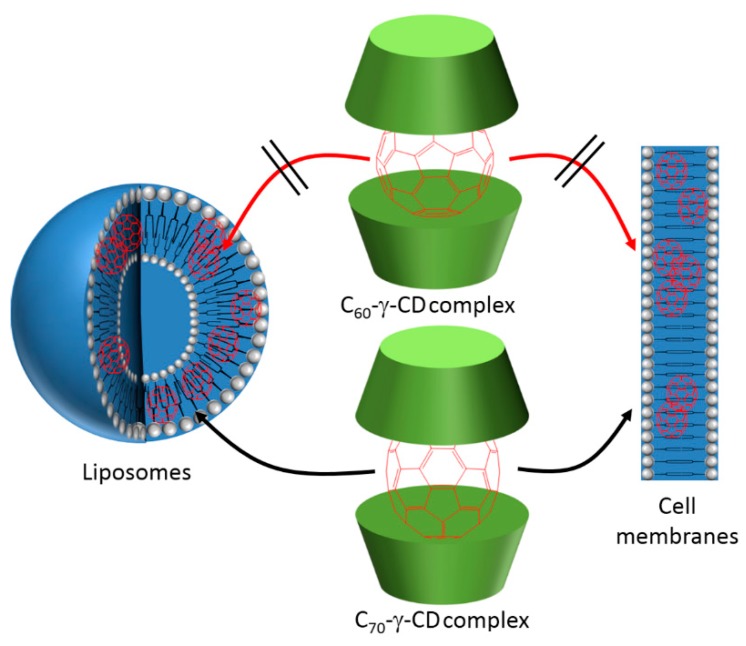
Schematic illustration of the exchange reaction from γ-CD cavity to the liposomal and cell membranes at 37 °C. Adapted from Ikeda; Matsumoto; Akiyama; Kikuchi; Ogawa; Takeya [214].

Ikeda et al. synthesized an inclusion complex that was constituted of fullerene C_x_ (x = 60 or 70) and γ-CD to implement the same concept but on the cellular membrane [214] (Figure 63). The intracellular uptake of both C_60_ and C_70_ from C_60_-γ-CD and C_60_-γ-CD inclusion complexes, respectively, was studied using human cervical HeLa cells ([C_60_] = [C_70_] = 20 µM). At 4 °C and 37 °C, C_60_-γ-CD preserved its encapsulated structure, as no transfer of C_60_ into the cells occurred. However, under the same conditions, C_70_ from C_70_-γ-CD were well incorporated into HeLa cells. Especially at 37 °C, 60% C_70_-γ-CD was uptaken by the cells after only 5 min of incubation. The stability and solubility C_70_-γ-CD was lower than that of the C_60_-γ-CD complex. The incorporation mechanism was the direct exchange reaction from the C_70_-γ-CD complex to the cell membrane. The fluorescence of rhodamine B 1,2-dihexadecanoyl-*sn*-glycero-3-phosphoethanolamine (RhB-DHPE) that was used to stain the HeLa cells was only quenched in the presence of C_70_-γ-CD. This result confirmed that C_70_ and RhB-DHPE coexisted in the cellular membrane. Under visible light irradiation (λ = 400–700 nm, 54 mW/cm^2^), the PDT efficiency against HeLa cells agreed with the results of the cellular uptake. In the dark, both complexes were not cytotoxic. Yet, as compared with the C_60_-γ-CD complex and 5-Aminolevulinic acid (5-ALA) as a reference, only C_70_-γ-CD triggered a drastic decrease of 75% of the cell viability, with only 1 µM of fullerene.

**Figure 64:**
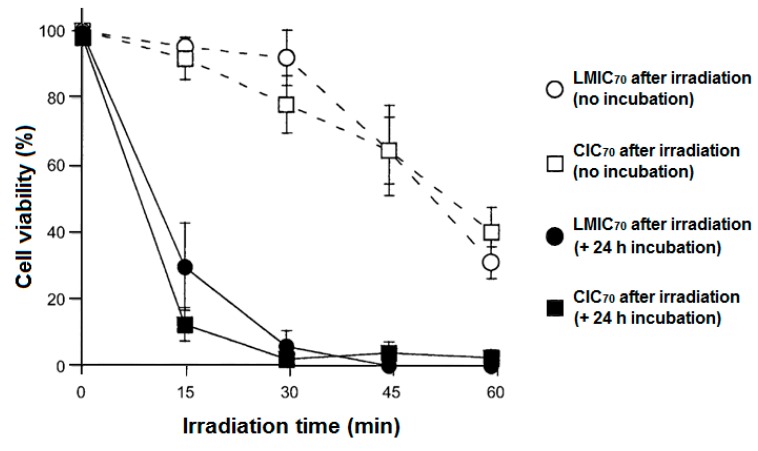
HeLa cell viability as a function of irradiation time (λ > 400 nm at 35 °C). Cells treated with LMIC_70_ and CIC_70_ and stained with propidium iodide immediately after irradiation and subsequent 24 h incubation (*n* = 3). Adapted from Ikeda; Nagano; Akiyama; Matsumoto; Ito; Mukai; Hashizume; Kikuchi; Katagiri; Ogawa; Takeya [215].

Encouraged and inspired by the good PDT activity obtained with LMIC_70_ (λ > 400 nm) and C_70_-γ-CD, the same team dedicated their efforts to improving this system for a higher stability and a more selective drug-delivery [215]. In this study, their cerasome-incorporated C_70_ (CIC_70_) system was based on encapsulating the C_70_ into a surface cross-linked liposomal bilayer covered with a polysiloxane surface (cerasome). This system was formed by the transfer of C_70_ from the cavities of the γ-CD to occupy the vesicles of the cerasomes. The stability of the cerasomes was enhanced compared with the general liposomes by the deposition of silica on the surface of the cerasomes. C_70_ acted as a fluorescence quencher in the presence of RhB, which confirmed the encapsulation of this fullerene in the lipid membrane of the cerasomes. A complete transfer of C_70_ from the γ-CD cavities into the cerasomes was achieved at 25 °C after 1 min. The CIC_70_ system possessed a diameter of about 170 nm, which was considered suitable for implementing an enhanced permeability and retention (EPR) effect. In addition, CIC_70_ held high surface positive charges (+50.8 mV) that induced a good cellular uptake, yet was still slightly lower than that of LMIC_70_ (+57.2 mV). The presence of C_70_ improved the morphological stability of the cerasomes. For their application in PDT toward HeLa cells, CIC_70_ appeared to be non-cytotoxic in darkness and induced a forceful PDT effect that was as good as that displayed by LMIC_70_ under visible light irradiation (57 mW/cm^2^, 400–740 nm) (Figure 64).

**Figure 65:**
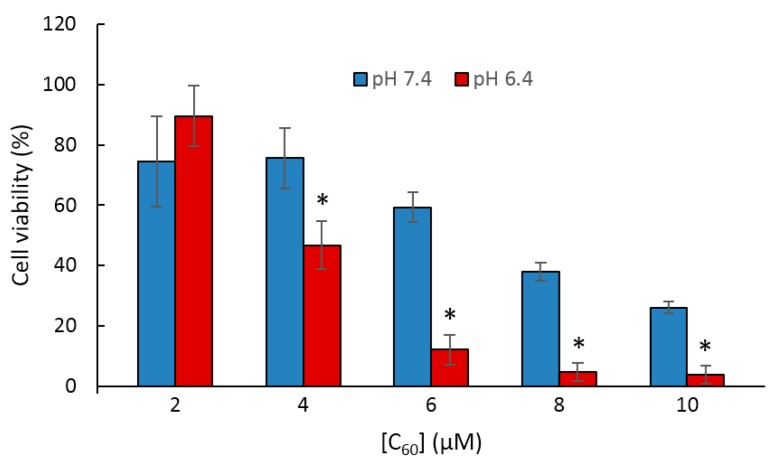
Cell viability of HeLa cells treated with C_60_-γ-CD-NH_2_ after irradiation (λ = 400–500 nm for 30 min). * *p* < 0.005 as compared to the PDT activity at pH 7.4. Adapted from Nobusawa; Akiyama; Ikeda; Naito [216].

**Figure 66:**
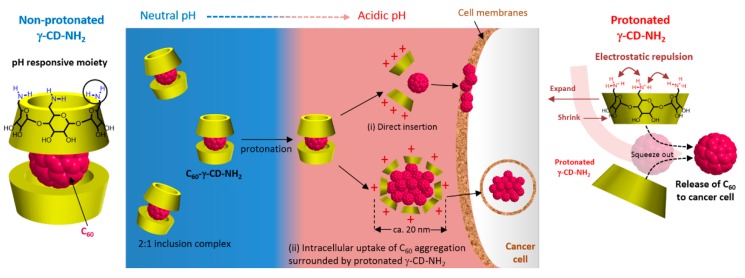
Schematic representation of efficient cellular uptake mechanisms and 2:1 C_60_-γ-CD-NH_2_ inclusion complex under neutral and acidic pH. Adapted from Nobusawa; Akiyama; Ikeda; Naito [216].

On this basis, Ikeda et al. [216] used a γ-CD derivative holding primary amine groups as pH responsive functions to carry their C_60_ (C_60_-γ-CD-NH_2_). To validate the efficiency of the γ-CD-NH_2_ carrier compared with γ-CD, the in vitro PDT activity of C_60_-γ-CD-NH_2_ and C_60_-γ-CD toward HeLa cells under visible light illumination (400–500 nm) was assessed. The experiments were conducted at two pH values, 7.4 and 6.4, which correspond respectively to the extracellular pH of normal and neoplastic cells. Both C_60_-γ-CD-NH_2_ and C_60_-γ-CD exhibited no dark toxicity, and negligible photoinduced toxicity was attained with C_60_-γ-CD under both pH conditions. As for C_60_-γ-CD-NH_2_, it was photoactive in destroying HeLa cells at both pH values, with an enhanced activity witnessed at pH 6.4 (Figure 65). Thus, it was revealed that as the pH started to become more acidic, the amine groups of the γ-CD-NH_2_ became gradually protonated up to a critical pH, i.e., 6.7, where the C_60_ was rapidly squeezed out of the γ-CD-NH_2_ into the extracellular region. The released C_60_ were rapidly uptaken by the cells through either endocytosis or direct insertion to the cellular membrane (Figure 66). This γ-CD-NH_2_ system represented a smart carrier of C_60_ for a more efficient application in PDT.

**Figure 67:**
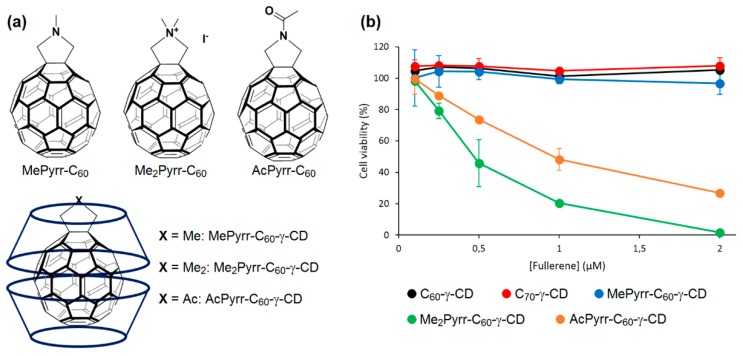
(**a**) Chemical structure of C_60_ derivatives and C_60_ derivatives-γ-CD inclusion complexes. (**b**) Concentration-dependent cytotoxicity of C_60_-γ-CD, C_70_-γ-CD, *N*-methylpyrrolidine (MePyrr)-C_60_-γ-CD, *N*,*N*-dimethylpyrrolidinium iodide (Me_2_Pyrr)-C_60_-γ-CD, and *N*-acetylpyrrolidine (AcPyrr)-C_60_-γ-CD on Hela cells under irradiation (λ = 610–740 nm for 30 min). Adapted from Ikeda; Iizuka; Maekubo; Aono; Kikuchi; Akiyama; Konishi; Ogawa; Ishida-Kitagawa; Tatebe; Shiozaki [213].

In 2013, Ikeda et al. addressed the problem of the stability and solubility of the C_x_-γ-CD inclusion complexes that affected their performance in PDT [213]. To form a stable inclusion complex, the authors used C_60_ derivatives holding nitrogen atom-containing groups, namely *N*-methylpyrrolidine (MePyrr, amino group), *N*,*N*-dimethylpyrrolidinium iodide (Me_2_Pyrr, ammonium group), and *N*-acetylpyrrolidine (AcPyrr, amide group) instead of C_60_ (Figure 67a). Compared with C_60_-γ-CD, all of the γ-CD-complexed C_60_ derivatives (MePyrr-C_60_-γ-CD, Me_2_Pyrr-C_60_-γ-CD and AcPyrr-C_60_-γ-CD) were found similarly water-soluble but more stable. In the absence of an irradiation source, the complexes were non-cytotoxic. The photoirradiation was performed at high wavelengths (610-720 nm). Under irradiation, Me_2_Pyrr-C_60_-γ-CD followed by AcPyrr-C_60_-γ-CD complexes exhibited a superior PDT activity against HeLa cells as compared to Photofrin^®^-γ-CD, C_60_-γ-CD, C_70_-γ-CD and MePyrr-γ-CD (Figure 67b). This superior photoactivity, especially for Me_2_Pyrr-C_60_-γ-CD, was attributed to the increased cellular uptake caused by the cationic nature of the surface of this complex vs. the other two neutral complexes MePyrr-γ-CD and AcPyrr-C_60_-γ-CD. In addition, higher levels of ^1^O_2_ were elaborated by Me_2_Pyrr-C_60_-γ-CD. The suppressed PDT activity of MePyrr-γ-CD complex was imputed to the low ^1^O_2_ production caused by the quenching effect of the triplet excited state of this complex by the lone pair of the electrons on the amino groups.

After they proved that using C_60_ derivatives with the γ-CD improved the cellular uptake and therefore the PDT activity compared with C_60_-γ-CD and C_60_-γ-CD, Ikeda et al. used those γ-CD-complexed C_60_ derivatives to prepare lipid membrane-incorporated C_60_ derivatives by an exchange method [210]. The C_60_ derivatives were transferred from the two cavities of γ-CD into the lipid membranes of the liposomes. In a similar trend, Me_2_Pyrr-C_60_-γ-CD incorporated into the lipid membranes (Me_2_Pyrr-LMIC_60_) resulted in the higher PDT activity toward HeLa cells under excitation between 610–740 nm as compared to the other C_60_ derivatives. The cationic nature of the incorporated Me_2_Pyrr-C_60_ did not induce any large effect on the surface potential of the Me_2_Pyrr-LMIC_60_ that might affect their internalization into the cells. Thus, the intercellular uptake of all of the tested liposomes was comparable. The source of this higher PDT activity of Me_2_Pyrr-LMIC_60_ was the greater ability to produce ^1^O_2_, since even more ^1^O_2_ was generated by Me_2_Pyrr-LMIC_60_ than that produced by the Me_2_Pyrr-C_60_-γ-CD inclusion complex.

**Figure 68:**
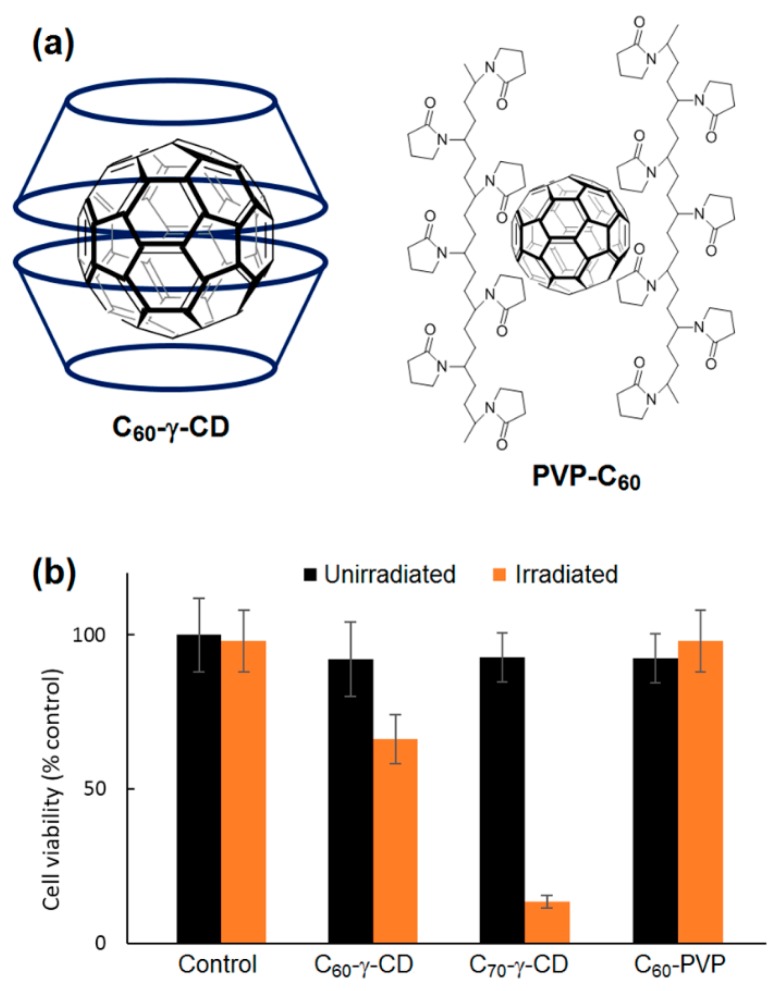
(**a**) Chemical structure of unmodified C_60_ and C_70_ dispersed in water using γ-CD (C_60_-γ-CD) and poly(vinylpyrrolidone) (C_60_-PVP). (**b**) 5RP7 cell viability (WST-1 assay). Cells treated with C_60_-γ-CD, C_70_-γ-CD, and C_60_-PVP aqueous solutions under irradiation (λ = 633 nm, 3 mW/cm^2^ for 1 h) compared to negative control (cell untreated). Adapted from Iizumi; Okazaki; Zhang; Yudasaka; Iijima [217].

The good photophysical properties of fullerenes nominate them as potential PSs in PDT. However, their scarce solubility in aqueous media hinders their biological application. To overcome this issue, Iizumi et al. [217] prepared water-soluble fullerene derivatives. The unmodified C_60_ and C_70_ were dispersed in water using γ-CD (C_60_-γ-CD and C_70_-γ-CD) or poly(vinylpyrrolidone) (C_60_-PVP) (Figure 68a). In C_60_-γ-CD and C_70_-γ-CD complexes, both fullerenes produced an appreciable amount of ^1^O_2_ in water as compared to Rose Bengal (RB). Yet, the produced ^1^O_2_ was less than that produced by fullerenes alone in organic solvents, most probably due to their inclusion into the cavities of γ-CD. This might have disrupted the efficient contact and accordingly the energy transfer between fullerenes and the molecular oxygen. Lower levels of ^1^O_2_ were produced by C_60_ in C_60_-PVP. The assumption that C_60_ tended to aggregate with the PVP dispersant while it formed a stable complex with γ-CD as monomers was possibly the reason behind the difference in ^1^O_2_ generation. Dark toxicity and phototoxicity (λ = 633 nm, 3 mW/cm^2^) were performed on the rat fibroblast cell line 5RP7 in the presence of C_60_-γ-CD, C_70_-γ-CD and C_60_-PVP with fullerene concentration fixed at 10 µM. Slight dark toxicity was revealed in all cases. Nevertheless, and in coherence with the ^1^O_2_ results, C_70_-γ-CD exhibited a massive phototoxic impact leaving only 12% of the cells alive (Figure 68b). Although C_60_-γ-CD was proved analogous to C_70_-γ-CD in terms of the ^1^O_2_ generation, a much lower PDT effect was observed (66% of cell survival). This was most probably due the much lower extinction coefficient of C_60_-γ-CD at 633 nm. Conversely, C_60_-PVP mediated no cell death under irradiation. Thus, the phototoxicities of C_60_ and C_70_ were greatly influenced by their dispersed forms.

**Figure 69:**
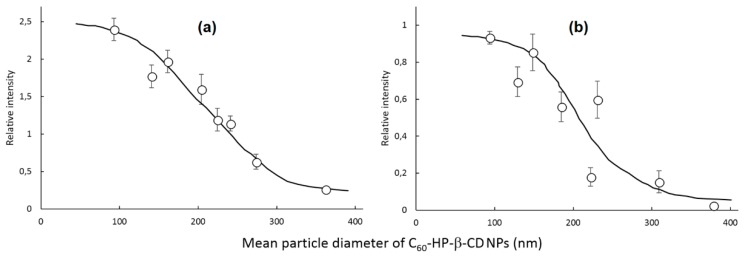
Particle size dependencies of C_60_ for the generation of (**a**) HO• and (**b**) ^1^O_2_ after visible light irradiation supplied from a fluorescence lamp (3500 lux, λ = 400–700 nm, 2 cm from the bottom) for 15 min. Each point represents the mean ± S.E. of three to five experiments. Adapted from Iohara; Hiratsuka; Hirayama; Takeshita; Motoyama; Arima; Uekama [218].

In 2011, Iohara et al. [218] prepared C_60_-HP-β-CD NPs by a co-grinding method. The authors compared the different properties of those NPs to C_60_ dispersed in poly(vinylpyrrolidone) (PVP). It appeared that when HP-β-CD was used as a dispersant for C_60_, a much lower mean particle diameter was attained (90 nm) than the one obtained in PVP (215 nm). This outcome agreed with the results obtained by Iizum et al. [217]. ^1^O_2_ was already proved to be elaborated from C_60_-HP-β-CD NPs through a type-II mechanism. Yet, in addition to ^1^O_2_, the authors revealed the great aptitude of those NPs to generate multiple ROS, including HO^•^ and O_2_^•^^−^ under visible light irradiation through a type I reaction. The increase in the particle size due to the aggregation caused a restricted penetration of light that consequently led to a gradual loss of the capability of C_60_-HP-β-CD NPs to produce ROS (Figure 69). C_60_-PVP and C_60_ alone produced lower levels of HO^•^ and O_2_^•^^−^, and barely generated ^1^O_2_. In the dark, C_60_, C_60_-PVP, and C_60_-HP-β-CD did not cause the death of HeLa cells, even at high concentrations. C_60_ remained non-toxic, even under visible light irradiation. In contrary, C_60_-PVP and C_60_-HP-β-CD prompted an interesting dose-dependent phototoxic effect. Bringing up their high capacity to produce different ROS, it was not surprising that C_60_-HP-β-CD NPs induced the most significant PDT effect among all. Those results assured the crucial role of HP-β-CD in elaborating such a stable and efficient system.

**Figure 70:**
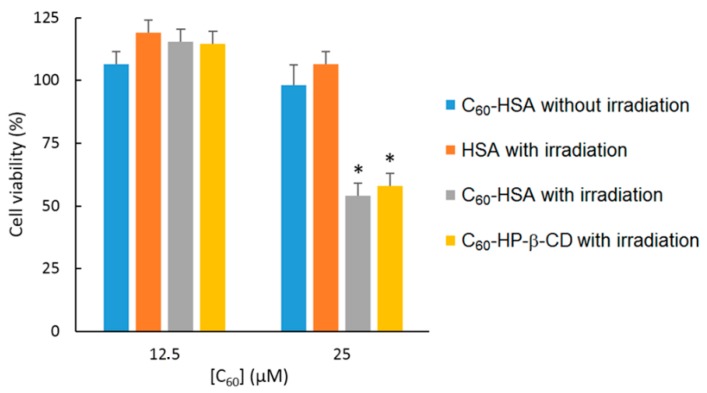
PDT activity of human serum albumin (HSA) solution alone, HSA-C_60_ solution, and C_60_-HP-β-CD NPs on A549 cells in the dark or after irradiation (λ = 400-700 nm, 35 mW/cm^2^ for 30 min). Each point represents the mean ± SE of five experiments. * *p* < 0.05 vs. HAS alone solution. Adapted from Altaf; Aldawsari; Banjar; Makoto; Daisuke; Masaki; Kaneto; Fumitoshi [219].

Many scientific efforts were devoted to render the fullerenes water-soluble for biological applications. Those attempts include the solubilization of those fullerenes by CDs through inclusion complexes, as previously presented by Ikeda et al. in their water-soluble host–guest fullerene–liposomes. On a similar track, Altaf et al. [219] prepared their C_60_-HSA NPs where the C_60_ were transferred from the C_60_/HP-β-CD NPs to the human serum albumin (HSA). The elaborated C_60_-HSA NPs did not aggregate and exhibited a narrow size distribution and good dispersion stability. When linked to C_60_, the fluorescence intensity of HSA decreased. C_60_-HSA held a slightly improved high-affinity binding and a larger scavenging activity, and produced much more multiple ROS under visible light (^1^O_2_ and O_2_^•^^−^) than HSA alone. HSA, C_60_-HSA, and C_60_-HP-β-CD did not exhibit dark cellular toxicity. However, the visible light-induced PDT effect of C_60_-HSA NPs toward A549 cells was interesting, and analogous to that displayed by C_60_-HP-β-CD NPs, and negligible in the case of HSA alone (Figure 70). In conclusion, β-CD helped elaborate a potential drug delivery system for PDT.

**Figure 71:**
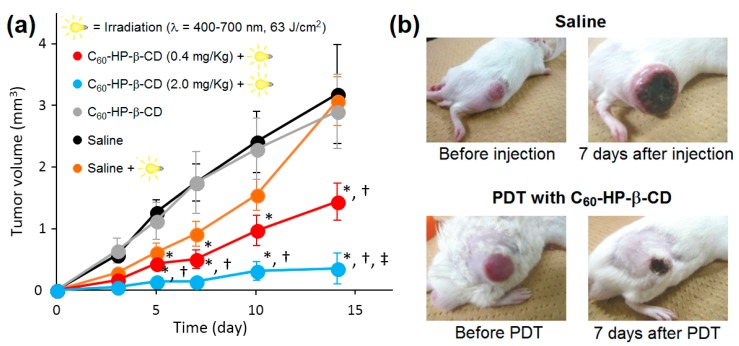
PDT effect of C_60_-HP-β-CD NPs (**a**) on tumor growth and (**b**) changes in body weight of ddY mice in the dark or after irradiation from a xenon light source (λ = 400–700 nm, 63 J/cm^2^, 350 mW/cm^2^). Each point represents the mean ± S.E. of six to nine experiments. * *p* < 0.05 vs. saline. ^†^ *p* < 0.05 vs. saline plus light irradiation. ^‡^ *p* < 0.05 vs. C_60_-HP-β-CD (0.4 mg/Kg) plus light irradiation (63 J/cm^2^). Adapted and reproduced from Altaf; Aldawsari; Banjar; Iohara; Anraku; Uekama; Hirayama [220].

In vitro and in vivo experiments were also conducted on C_60_/HP-β-CD NPs to assess their photosensitizing activity for PDT [220]. In those NPs, C_60_ partially occupied the hydrophobic cavity of CD. Under visible light irradiation, this system generated a high level of ^1^O_2_ (φ_Δ_ = 0.96 in benzene) that even exceeded that produced by PpIX (φ_Δ_ = 0.56 in PBS). In addition, O_2_^•^^−^ was also produced by the C_60_ component of the NPs. The generation of both types of ROS was dose-dependent, and it progressively and proportionally increased with the increase of the irradiation time and the energy of the light. In vitro, only when illuminated by visible light, C_60_-HP-β-CD NPs caused remarkable photoinduced toxicity toward HeLa and A549 cells, with IC_50_ values of 10 µM and 60 µM, respectively. C_60_-HP-β-CD NPs acted better as PDT PS than 5-ALA, which was probably due to the greater ROS production. Moreover, the authors investigated the most appropriate parameters for an efficient in vivo PDT. In the presence of C_60_-HP-β-CD NPs (2 mg/Kg of C_60_), 12 times repeated visible light irradiation (λ = 400–700 nm, 350 mW/cm^2^) for short period (15 s) with a total light dose of 63 J/cm^2^ efficiently suppressed the growth of the sarcoma S-180 cells in ddY mice without causing any skin damage or major hyperthermia (Figure 71).

**Figure 72:**
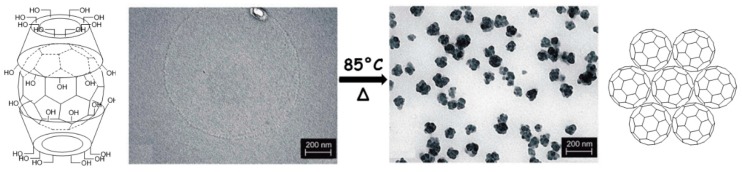
Visual and TEM images of C_60_-γ-CD and C_60_ aggregates after 150 min of heating at 85 °C. Reproduced and adapted by permission of The American Chemical Society [221].

**Figure 73:**
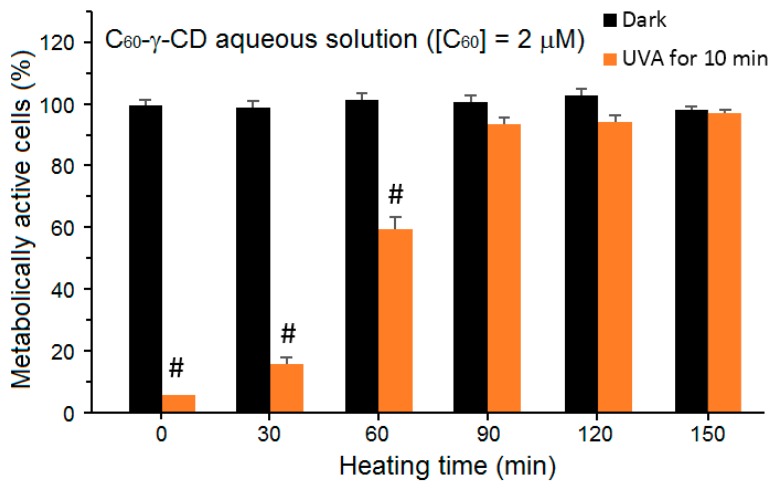
Effect of 2-μM fullerene water solutions as a function of heating time on the viability of HLE B-3 cells irradiated with UVA. Results were presented as the means ± SEMs from three independent experiments in quadruplicate. ** *p* < 0.01 and * *p* < 0.05 as compared with cells without fullerene treated and in the dark. ^#^ *p* < 0.01 as compared with corresponding cells in the dark. Adapted from Zhao; He; Chignell; Yin; Andley; Roberts [221].

Zhao et al. also addressed in their study the problem of the lack of solubility of fullerene in water [221]. The authors prepared a supramolecular complex that was constituted of fullerene and γ-CD. This complex appeared to be water-soluble at room temperature, but tended to aggregate when heated at 85 °C (Figure 72). The aggregated complexes were much more incorporated into the lens epithelial cells HLE B-3. In darkness and under visible light illumination, the monomeric and aggregated C_60_-γ-CD displayed no toxicity. Nevertheless, under UV-A irradiation, the viability of the HLE B-3 cells exposed to the monomeric C_60_-γ-CD decreased sharply to less than 10% in a dose-dependent manner. As for the aggregated fullerenes, the PDT efficiency was gradually lost with the gradual growth of the aggregates, and finally vanished when the cells were treated with the largest aggregates attained after 150 min of heating at 85 °C. The production of ^1^O_2_ followed the same trend, where the maximum amount was generated by C_60_-γ-CD, and decreased proportionally with the increasing aggregate size (Figure 73). After UVA irradiation, the ^1^O_2_ was generated by C_60_-γ-CD inside the cells, and it targeted the intracellular proteins in HLE B-3 cells. This outcome was confirmed by the presence of high amounts of protein peroxides in the cells, which led to their damage by the induced apoptosis.

**Figure 74:**
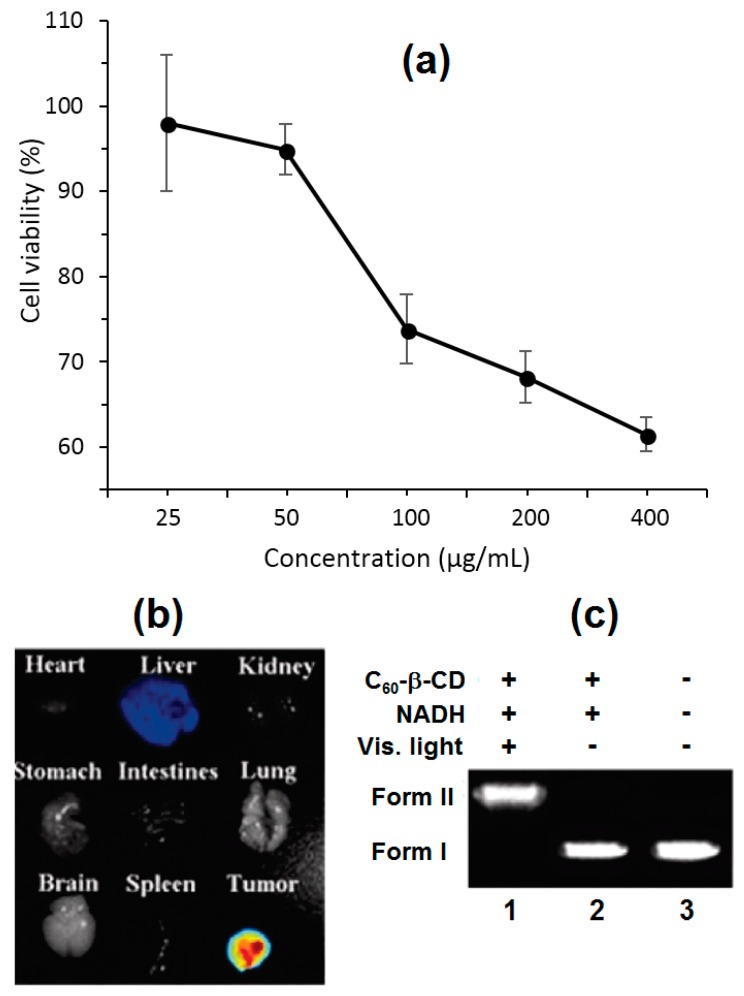
(**a**) In vitro cytotoxicity of C_60_-β-CD against human neuroblastoma SH-SY5Y cells upon visible light for 20 min. (**b**) Near Infrared Fluorescence (NIRF) images of different organs at 96 h after tail-vein injection of the NIR-797-labeled C_60_-β-CD. (**c**) Photoinduced DNA cleavage behavior of C_60_-β-CD. The pBR322 supercoiled plasmid was incubated with each chemical for 4 h at room temperature. Lane 1: pBR322 DNA (0.017 μg/μL) with C_60_-β-CD (33 μmol/L), NADH (330 μmol/L), incubated under visible light irradiation. Lane 2: pBR322 DNA (0.017 μg/μL) with C_60_-CD (33 μmol/L), NADH (330 μmol/L), incubated in dark. Lane 3: pBR322 DNA (0.017 μg/μL) incubated in the dark. Reproduced and adapted by permission of The John Wiley and Sons [222].

In their approach, Wang et al. [222] associated the fullerene C_60_ to the outside of the β-CD molecule to yield a water-soluble exclusion complex rather than embedding it in the cavities. C_60_ and β-CD were linked together via diaminotriethylene glycol as a hydrophilic spacer. Both the hydrophilic spacer and β-CD maintained an enhanced solubility of C_60_ in water. The absorption profile and thus the basic structure of C_60_ remained intact after being conjugated to β-CD. On the one hand, when this C_60_-β-CD was introduced in different concentrations up to 200 µg/mL into the human neuroblastoma SH-SY5Y cells, a negligible dark toxicity was witnessed. On the other hand, under irradiation with visible light (λ ≥ 400 nm, 20 min), C_60_-β-CD revealed a dose-dependent phototoxicity against the tumor cells (Figure 74a). After investigating the in vitro cytotoxicity and phototoxicity of their C_60_-β-CD, the authors aimed to assess the dispersion distribution of the C_60_-β-CD throughout the living body. For actions to serve this goal, they labeled C_60_-β-CD with a NIR-dye, i.e., NIR-797, and injected it into hepatic H22 tumor-bearing mice. At 96 h post-injection, C_60_-β-CD was massively localized in the tumor and scarcely in the liver (Figure 74b). C_60_-β-CD appeared to circulate throughout the blood with a half-life of about 4 h, leaving behind no acute or subacute toxicity in the different organs of the mice’s body. Only in the presence of visible light and nicotinamide adenine dinucleotide (NADH), which is a reducing agent that naturally assists in the photocleavage of DNA, was C_60_-β-CD was active toward the cleavage of pBR322 plasmid DNA into form II (Figure 74c). Both the ability of C_60_-β-CD to cleave DNA and kill tumor cells by PDT were due to the action of ROS, precisely HO^•^ and O_2_^•^^−^, which were significantly produced by the excited PS.

**Figure 75:**
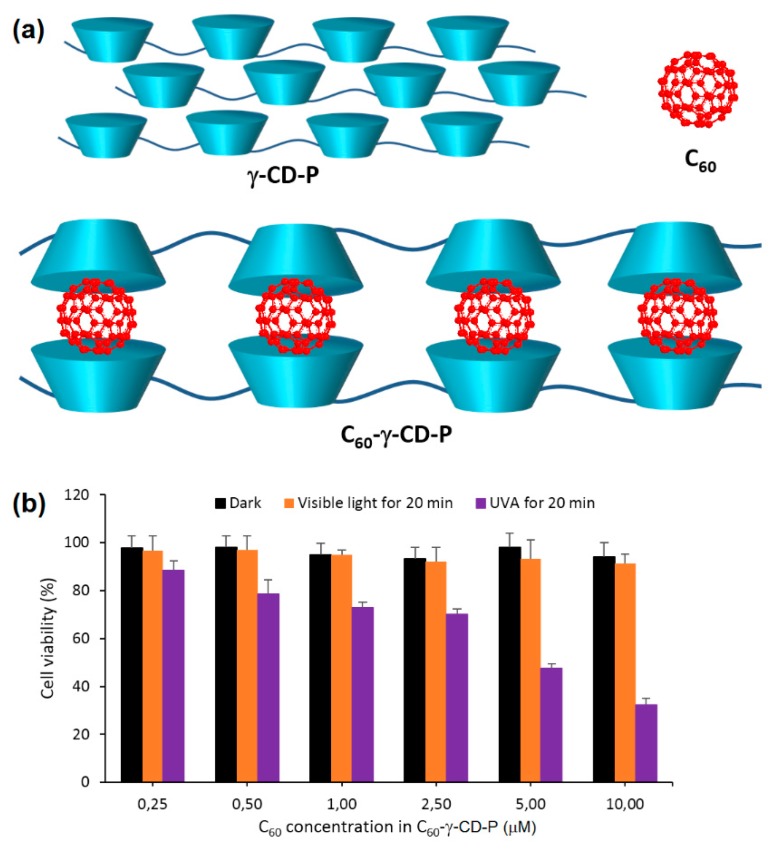
(**a**) Schematic illustration of γ-CD polymer (γ-CD-P), C_60_, and C_60_-γ-CD-P. (**b**) Effect of different concentration of C_60_-γ-CD-P exposure on the B16-F10 cell viability irradiated with UV-A from two fluorescent PUV-A lamps (Philips, PL-L36W) or two cool white visible light lamps (Philips, TLD36W) for 20 min. Adapted from Zhang; Gong; Liu; Piao; Sun; Diao [223].

To overcome the water-solubility problem of fullerene C_60_ and enhance its phototoxicity, Zhang et al. manipulated C_60_ via the pre-used CD-functionalization tactic [223]. However, instead of working with unmodified γ-CD, they used γ-CD polymer (γ-CD-P) to form their inclusion complex (C_60_-γ-CD-P) (Figure 75a). In this system, monomeric C_60_ occupied the hydrophobic cavities of γ-CD-P. Similar to β-CD and γ-CD, γ-CD-P successfully rendered C_60_ water-soluble, and the absorption spectrum of this inclusion complex displayed the characteristic peaks of C_60_. However, those peaks were red-shifted as compared to the absorption peaks obtained from C_60_-γ-CD-P. However, γ-CD-P appeared to be capable of forming inclusion complexes easier than γ-CD. In addition, C_60_ in the C_60_-γ-CD-P complex remained well-dispersed in water without forming aggregates, even at high concentrations of the complex due to the supramolecular interaction with γ-CD-P, which was an advantage over the other C_60_ inclusion complexes with CD. The C_60_-γ-CD-P complex efficiently produced ^1^O_2_ only under UVA light, unlike C_60_-β-CD, which can generate ^1^O_2_ under both visible and UVA lights. Those complexes were studied in vitro (MTT assay) with mouse melanoma cell lines B16-F10. γ-CD-P increased the biocompatibility of C_60_, as no cytotoxic was observed when the cells were exposed to different concentrations of C_60_-γ-CD-P ([C_60_] = 0.5–20 µM) after 48 h of incubation in the dark. Due to the non-aggregated nature of the C_60_ enclosed into γ-CD-P and in agreement with the significant ^1^O_2_ production, C_60_-γ-CD-P exhibited a superior phototoxic effect than C_60_ toward the tumor cells under UVA irradiation (Figure 75b).

## 3. Conclusions and Perspectives

As a conclusion, we can notice that all of the papers describing the use of CDs stress how important CD–PS systems are in improving PDT efficiency. Various CD–PS systems have been developed and can be formed by three types of binding mode of PSs with CDs, which are non-covalent binding (CD–PS inclusion complexes), covalent binding (CD–PS conjugates), and non-specific external binding (CD–PS nanoassemblies). For porphyrinoid PSs, it was found that CD–PS inclusion complexes can: (1) improve PDT efficiency [120,121,126] compared with free PSs; (2) reduce some drawbacks of PSs such as their lack of water solubility [120,121,124,125,126] and self-aggregation [124,127]; (3) induce a lower cytotoxicity than free PSs in some cases [120]; (4) lead to a better or different cellular uptake [125,129] even in spheroids [128]; (5) modify the intracellular distribution [127]; and (6) induce an acceleration of the PS diffusion into the biological media [127]. Similar results are also obtained for non-porphyrinoid PSs. 

Moreover, some CD–PS conjugates and nanoassemblies have proven their usefulness to encapsulate (1) chemotherapeutic drugs allowing both PDT and chemotherapy with a synergistic effect [140,141,144,167,171,183], or (2) NO photodonor, enabling an amplified PDT effect due to the simultaneous photorelease of two cytotoxic species (^1^O_2_ and NO^•^) [145,177].

In addition, the combination of CD–PS systems with NPs leads to passive-targeting PDT treatment, thanks to the EPR effect of NPs. NIR excitation-type NPs (UCNPs) allow also to reach deeper tumors due to their better penetration into biological tissues [73,192,193,194], and AuNPs enable doing PTT and PDT simultaneously [197].

With the aim of improving the selectivity of all of these CD–PS systems, we believe that in the near future, new CD–PS systems combined to active targeting agents will emerge. In this regard, few articles already describe the grafting of active targeting agents on CD–PS systems, i.e., grafting of cell penetrating peptide R_6_H_4_ [224], membrane-permeable and mitochondria-targeted peptide RLA [225], CD44-targeted hyaluronic acid [226], folate-targeted folic acid [227], and asialoglycoprotein-targeted sugars (galactose [228] and lactobionic acid [229]). 

## Figures and Tables

**Table 1:** Estimated cancer deaths worldwide and top five killer cancers for both sexes combined, 2012 (International Agency for Research on Cancer (IARC) data) and 2016 (Institute for Health Metrics and Evaluation (IHME) data).

Year	Global Cancer Death	Top Five Killer Cancers				
		Lung ^1^	Liver	Stomach	Colorectal	Breast
2012	8.2 million	1.59 million	745,517	723,027	693,881	521,817
2016	8.9 million	1.71 million	828,945	834,171	829,558	545,590
Rates of change ^2^	+8.6%	+7.6%	+11.2%	+15.4%	+19.6%	+4.6%

^1^ Included tracheal, bronchus, and lung cancer. ^2^ Cancer mortality rates from 2012 to 2016.

**Table 2:** Overview of clinically approved PSs for PDT as cancer treatment.

PS	Trademark	Approved	λ_exc._ (nm)	Application
Porfimer sodium	Photofrin	WW ^9^	632	Bladder, esophogeal, lung, cervical, endobronchial, and gastric cancers
5-ALA ^1^	Levulan/Ameluz	WW ^9^	635	Actinic keratosis
MAL ^2^	Metvix/Metvixia	WW ^9^	570–670	Actinic keratosis and basal cell carcinoma
HAL ^3^	Cysview/Hexvix	USA	635	Colon and bladder diagnosis cancer
mTHPC ^4^	Foscan	EU ^10^	652	Head and neck cancer
NPe6 ^5^	Laserphyrin	Japan	664	Lung cancer
Synthetic hypericin	SGX301 ^8^	EU ^10^/USA	570–650	Cutaneous T-cell lymphoma
LUZ11	Redaporfin ^8^	EU ^10^	749	Biliary tract cancer
AlPcS4 ^6^	Photosens	Russia	675	Stomach, skin, lips, oral cavity, tongue, and breast cancers
Pd-Bpheid ^7^	Tookad	EU ^10^	538/762	Prostate cancer

^1^ 5-Aminolevulinic acid. ^2^ Methyl aminolevulinate. ^3^ Hexyl aminolevulinate. ^4^ Meta-tetra(hydroxyphenyl)chlorin. ^5^ *N*-aspartyl chlorin e6. ^6^ Aluminium phthalocyanine tetrasulfonate. ^7^ Pd-bacteriopheophorbide. ^8^ Orphan status. ^9^ Worldwide. ^10^ European Union.

**Table 3:** Overview of natural and derived cyclodextrins (CDs).

Type of CD	Water Solubility (mg/mL)	Molecular Weight (Da)
***Natural CDs***		
α-CD	145	972
β-CD	18.5	1135
γ-CD	232	1297
***Chemically modified CDs***		
HP-β-CD ^1^	≥600	1400
SBE-β-CD ^2^	≥500	2163
RM-β-CD ^3^	≥500	1312
HP-γ-CD ^4^	≥500	1576
***Polymerized CDs***		
ECH-β-CD ^5^	>500	1.12 × 10^5^
ECH-CM-β-CD ^6^	>250	2 × 10^6^–15 × 10^6^

^1^ Hydroxypropyl-β-cyclodextrin. ^2^ Sulfobutyl ether-β-cyclodextrin. ^3^ Randomly methylated-β-cyclodextrin. ^4^ Hydroxypropyl-γ-cyclodextrin. ^5^ Epichlorohydrin cross-linked β-cyclodextrin polymers. ^6^ Epichlorohydrin cross-linked carboxymethyl-β-cyclodextrin polymers.

**Table 4:** IC_50_ value comparison of all conjugates against human colon adenocarcinoma (HT29) and human hepatocarcinoma (HepG2) cells.

Compound	IC_50_ (µm) HT29	IC_50_ (µm) HepG2
PMe-β-CD-hexyl-Si^IV^Pc	0.04	0.05
PMe-β-CD-DEG-Si^IV^Pc	0.16	0.17
PMe-β-CD-ethyl-Si^IV^Pc	0.91	1.32
PMe-β-CD-TEG-Si^IV^Pc	0.14	0.15

**Table 5:** IC_50_ value comparison of PMe-β-CD-Si^IV^Pc conjugates **1**–**4** against HT29 and HepG2 cells.

PMe-β-CD-Si^IV^Pc Conjugates	IC_50_ (nm) HT29	IC_50_ (nm) HepG2
**1**	150	190
**2**	21	26
**3**	23	35
**4**	28	94

**Table 6:** Percent survival of Hela cells at different light doses (irradiance of 5 mW/cm^2^) of Cor(β-CD)_1_, Cor(β-CD)_2_, and Cor at a concentration of 10^−5^ M (*n* = 3).

Compound	Surviving Hela Cells Fraction (% ± SD) at Light Dose		
	6 J/cm^2^	9 J/cm^2^	12 J/cm^2^
Cor(β-CD)_1_	95.6 ± 0.6	81.6 ±3.2	81.6 ± 3.1
Cor(β-CD)_2_	120.7 ± 3.5	110.8 ± 0.5	110.4 ± 3.9
Cor	80.9 ± 3.0	44.5 ± 2.3	38.5 ± 1.4

**Table 7:** Cytotoxicity of the compounds toward A549R cells ^1^.

	IC_50_ (µM)		
	Cisplatin	Pt^IV^(β-CD)_2_/TPyP-(Ada)_4_	Pt^IV^(β-CD)_2_
Dark	23.2	4.6	> 20
Light ^2^	22.8	1.5	> 20

^1^ The IC_50_ values were calculated based on Pt^IV^ concentration. ^2^ Light irradiation (λ = 430 nm, 10 mW/cm^2^ for 2 min).
